# GPR37-enhanced ubiquitination of ATP1A1 inhibits tumor progression and radiation resistance in esophageal squamous cell carcinoma

**DOI:** 10.1038/s41419-024-07240-1

**Published:** 2024-12-27

**Authors:** Jiaru Hu, Fang Meng, Lei Lv, Fu Hong, Qing He, Qi Zhu, Tian Tian, Na Chang, Shiqiang Zhang, Qiyi Yi, Liting Qian

**Affiliations:** 1 Department of Radiation Oncology, Anhui Provincial Cancer Hospital, the First Affiliated Hospital of USTC, Division of Life Sciences and Medicine, University of Science and Technology of China, Hefei, 230001 China; 2 Department of Oncology & Hematology, Xishan People’s Hospital of Wuxi City, Wuxi, 214105 China; 3 Department of Cancer Epigenetics Program, The First Affiliated Hospital of USTC, Division of Life Sciences and Medicine, University of Science and Technology of China, Anhui Provincial Cancer Hospital, Hefei, 230031 China; 4 Department of Radiation Oncology, The First Affiliated Hospital of USTC, Division of Life Sciences and Medicine, University of Science and Technology of China, Anhui Provincial Cancer Hospital, Hefei, 230031 China; 5 Department of Respiratory Oncology, The First Affiliated Hospital of USTC, Division of Life Sciences and Medicine, University of Science and Technology of China, Anhui Provincial Cancer Hospital, Hefei, 230001 China; 6 Institute of Radiation Medicine, School of Basic Medical Sciences, Anhui Medical University, Hefei, 230032 Anhui China

**Keywords:** Cancer therapeutic resistance, Radiotherapy

## Abstract

Radiotherapy resistance is one of the main reasons for the dismal clinical outcome of patients with esophageal squamous cell carcinoma (ESCC). Therefore, clarifying the targets and molecular mechanisms of radiotherapy resistance in ESCC is of great theoretical and clinical significance to enhance the efficacy of radiotherapy. In this study, GPR37 was identified as a key factor facilitating ESCC radiosensitization. We found that GPR37 is lowly expressed in ESCC, especially in radioresistant ESCC tumors. And its insufficiency is related to the malignant characteristics and unfavorable prognosis in ESCC. Further investigation revealed that GPR37 level is inversely regulated by promoter methylation but positively regulated by ZNF750. Functionally, GPR37 could not only overcome radioresistance of ESCC, but also inhibit proliferation, migration, and invasion. Mechanistically, GPR37 interacts with the ATP1A1 protein, effectively promoting its ubiquitination-induced degradation, thereby limiting the activation of the AKT/mTOR signaling pathway. Additionally, GPR37 can be transported to recipient cells via exosomes and inhibit the malignant behavior of recipient cells. Overall, these findings suggest that GPR37-ATP1A1 axis holds potential as a therapeutic target for the management of ESCC, especially for overcoming radiation resistance.

## Introduction

Esophageal cancer ranks sixth in terms of global cancer mortality and is among the most common types of gastrointestinal cancers worldwide [1–4]. It is mainly primarily categorized into two types: esophageal adenocarcinoma (EAC) and esophageal squamous cell carcinoma (ESCC), of which the latter constitutes approximately 90% of all cases [5]. Typically, locally advanced ESCC is treated with radiotherapy (RT), chemoradiation therapy (CRT) or neoadjuvant CRT followed by surgery [3, 6]. Thus, radiotherapy plays a central role in the treatment plan, especially for ESCC patients who are either unable or unwilling to proceed with surgical intervention. However, up to 80% of patients treated with radiotherapy experience treatment failure due to tumor recurrence or distant metastasis within the radiation field [7]. This failure can be attributed in part to the presence of radiotherapy-resistant cells, whether intrinsic or induced by radiation [2, 8]. Thus, the efficacy of radiotherapy in ESCC patients is crucial for their prognosis, highlighting the necessity for a more profound insight into the molecular underpinnings and targets involved in ESCC development and radioresistance.

GPR37 belongs to the G protein-coupled receptors (GPCRs) family, also known as parkin-associated endothelin-like receptor (Pael-R) [9]. It is an orphan receptor that lacks an endogenous ligand for triggering GPCR signaling, which hinders the study of its function. Notably, GPR37 is a substrate for the E3 ubiquitin-protein ligase parkin and a component of the proteasome pathway [10]. Therefore, proteins that directly interact with it may be affected by proteasomal degradation. GPR37 is highly expressed within the central nervous system, with the majority of research to date focusing on its association with neurological disorders, including Parkinson’s disease, inflammation, pain, autism, and brain tumors [11–16]. In the field of oncology, evidence has emerged demonstrating that GPR37 serves a critical function in the malignant advancement of certain types of tumors. Notably, it has been observed to enhance the proliferation and metastasis of lung and colon cancers [17–21]. Conversely, GPR37 exhibits reduced expression in hepatocellular carcinoma and multiple myeloma cell adhesion models, and its downregulation has been linked to tumor progression and an unfavorable prognosis of these tumors [22, 23]. Thus, the precise function of GPR37 contingent upon the tumor type. Notably, in one of our previous studies, we found that GPR37 was the most significantly down-regulated gene expressed in radiation resistance-derived ESCC cell lines [24]. However, the expression of GPR37, along with its regulatory mechanisms, the exact biological function, and especially the role and mechanism of radiotherapy resistance in ESCC is still unclear.

Exosomes are small vesicles with a diameter of 30-200 nm [25]. They contain a diverse array of molecules, such as proteins, nucleic acids, and lipids [25, 26]. These nanovesicles serve as critical mediators in intercellular communication by shuttling their cargo to recipient cells, thereby influencing a wide spectrum of physiological and pathological processes [25, 27, 28]. Notably, there is compelling evidence linking exosomes to tumor progression in ESCC [29, 30]. Purbasha Bhattacharya et al. found that exosomes could exert their effects through GPR37 [13]. And Xingyu Du et al. showed that exosomes could confer radioresistance to ESCC [31]. However, the underlying mechanisms by which exosomes modulate ESCC radioresistance are still not fully understood.

In this study, we elucidated GPR37 as a critical radiosensitive gene in ESCC. Our findings revealed that GPR37 is downregulated in ESCC and is inversely correlated with unfavorable clinical outcomes. Furthermore, we demonstrated that GPR37 impedes tumor progression and enhances sensitivity to radiation in ESCC. We further explored the regulatory mechanisms underlying GPR37 expression, focusing on the roles of DNA methylation and ZNF750. Our research established that GPR37 interacts with ATP1A1 to promote its ubiquitinated degradation, which in turn inhibits AKT phosphorylation and then suppresses ESCC proliferation, migration, and DNA damage repair processes. Furthermore, we identified the presence of GPR37 protein within exosomes, which has the potential to modulate the development and radioresistance of recipient tumor cells. These discoveries underscore the promise of GPR37 as a new therapeutic and radiotherapeutic target in ESCC, providing a possible treatment strategy and offering new insights for the clinical management of ESCC.

## Materials and methods

### Tissue specimen collection from patients

The samples of 97 ESCC patients included in this study were from patients who underwent surgery in Anhui Provincial Hospital from 2011 to 2013. This study also included 5 pairs of fresh ESCC tissue and corresponding adjacent normal tissue from 5 patients who underwent ESCC surgery in 2022. All study protocols were approved by the Medical Research Ethics Committee of Anhui Provincial Hospital (Approval No. 2021KY-140), and written informed consent was obtained from all patients.

### Immunohistochemistry

Perform immunohistochemical staining as described in the article [32]. Sections were stained with anti-GPR37 (Invitrogen cat. #PA5-13412) primary antibody. Immunohistochemistry slides are evaluated and grouped by two pathologists who are blinded to clinical information.

### Data from TCGA and GEO

The clinicopathological data, DNA methylation, and mRNA expression data from TCGA were accessed and downloaded via the UCSC Xena platform (https://xenabrowser.net/datapages/). Specifically, the “DNA methylation—Methylation450k” and “IlluminaHiSeq pancan normalized gene expression” datasets were employed in our analysis. Only samples with complete information were included in our analysis.

To further investigate the correlation between GPR37 expression and the prognosis of esophageal squamous cell carcinoma (ESCC) patients, we also incorporated two ESCC datasets from the GEO database (GSE53622 and GSE53624 [33]). The mRNA sequencing data and corresponding patient survival details from these datasets were obtained directly from the GEO platform. And the analysis was confined to samples that possessed both comprehensive mRNA sequencing data and complete patient survival details.

### Gene set enrichment analysis

The correlation coefficients between GPR37 level and all other genes in the Cancer Genome Atlas (TCGA) ESCC were calculated by Spearman’s correlation analysis, and the pre-ranked list was imported into the Gene Set Enrichment Analysis (GSEA) software (version 4.3.2) for “h.all.v2023.2.Hs.symbols.gmt” analysis.

### Cell lines and cell culture

The ESCC cell lines (KYSE30, KYSE150, KYSE410, KYSE450, KYSE51 and TE-1) were obtained from the China Cell Resource Center (Shanghai, China), and the human embryonic kidney cell line 293 T (293 T) originated from the American Type Culture Collection (Rockville, MD, USA). All cell lines used were tested to be free of mycoplasma infection. All the ESCC cells were cultured at 37 °C, 5% CO_2_ in modified RPMI medium (Cytiva cat. #SH30809.01, US) supplemented with 1% (v/v) penicillin‒streptomycin (Beyotime cat. #C0222, Shanghai, China) and 10% (v/v) heat-inactivated fetal bovine serum (Gibco, MA, US). 293 T cells are cultured in DMEM (Cytiva, cat. #SH30022.01) with other conditions unchanged.

### Gene overexpression and knockdown experiments

GPR37 overexpressed lentivirus and negative control (vector NC) as well as lentivirus encoding shRNA targeting GPR37 were purchased from HanBio (Shanghai, China). The shRNA sequence of GPR37 is as follows: 5‘-GCTTCTCTGGGAGTCACCACTTTCAcgaaTGAAAGTGGTGACTCCCAGAGAAGC-3’ and 5’-CAACAACTGCCAAACTTGCTGTTATcgaaATAACAGCAAGTTTGGCAGTTGTTG-3’. PCR amplification of the full-length cDNA of ZNF750 and ATP1A1 was performed, followed by cloning into pSIN-3xFlag for lentivirus production. ZNF750 shRNA was donated by Mian Wu’s lab at the University of Science and Technology of China and purchased from the Sigma‒Aldrich shRNA library in accordance with the following sequence: 5’-CCGGGAATGACTCTAAGACTGATATCTCGAGATATCAGTCTTAGAGTCATTCTTTTTTG-3’. pSIN-3xFlag-ZNF750, pSIN-3xFlag-ATP1A1 or Plko.1-ZNF750-shRNA was transfected along with psPAX.2 and pMD2. G into 293 T cells, Attractene Transfection Reagent (Qiagen, Dusseldorf, GER) was used. After transfection, the supernatant containing the lentivirus was collected after 48 hours by filtration. The mixture of lentivirus was subsequently used to infect cells in the presence of polybrene (8 μg/ml). Following infection, cells were selected using puromycin (2 μg/ml) in regular culture media to obtain stably transfected cell lines after two weeks.

Parkin’s siRNAs were purchased from RiboBio (Guangzhou, China) with the following sequences: 5’-GTTTGTTCACGACCCTCAA-3’. Transfection was carried out using the riboFECT^TM^CP Transfection Kit (RiboBio, Guangzhou, China), and the transfection efficiency was detected after 48 hours.

### Western blot

The assay was performed as previously described [34]. The antibodies used were as follows: GPR37 (Invitrogen cat. #PA5-13412, MA, US), ZNF750 (Proteintech cat.# 21752-1-AP, Wuhan, China), FLAG (Proteintech cat.# 66008-4-IG), HA (Proteintech cat.# HRP-66006), N-cadherin (Proteintech cat.# 66219-1-Ig), E-cadherin (Proteintech cat.# 20874-1-AP), P-AKT (Proteintech cat.# 28731-1-AP), AKT (Proteintech cat.# 10176-2-AP), mTOR (Proteintech cat.# 28273-1-AP), RAD51 (Proteintech cat.# 14961-1-AP), Slug (Proteintech cat.# 12129-1-AP), Bcl-2 (Abmart cat.# T40056), ATP1A1 (Proteintech cat.# 14418-1-AP), CD63 (Cell Signaling cat.# 52090), tsg101 (Cell Signaling cat.# 72312), and GAPDH (Proteintech cat.# 60004-1-Ig). The secondary antibodies used were as follows: anti-rabbit IgG (Proteintech cat. #SA00001-2) and anti-mouse IgG (Proteintech cat. #SA00001-1). The primary antibodies are diluted 1:1000 and incubated overnight at 4 °C. And the secondary antibodies were diluted 1:5000 and incubated for 2 hours at room temperature. The blots were visualized using an enhanced chemiluminescence (ECL) hypersensitive chemiluminescent substrate (Biosharp, Beijing, China) and imaged using a Tanon 4600 automated chemiluminescence imaging system (Tanon, Shanghai, China).

### qRT‒PCR

The assay was performed as previously described [34]. The sequences of primers were as follows: GPR37 forward, GGTCAGGAGCCTTCTGAAACT, and reverse, CATTGGCCGTCTTGGACAG; ZNF750 forward, AGTCTCCTCAAAGAGCGGAAG, and reverse, GCAAGTAAAGGGACATTGGAAAC; Parkin forward, GTGTTTGTCAGGTTCAACTCCA, and reverse, GAAAATCACACGCAACTGGTC; and GAPDH forward, GGAGCGAGATCCCTCCAAAAT, and reverse, GGCTGTTGTCATACTTCTCATGG.

### Proliferation assay

To observe the cell growth rate, cells were seeded at 2000/well in 96-well plates, and when the cells were attached, CCK-8 solution was added to the cells at 0, 24, 48, 72, and 96 hours, incubated for 2 hours, and then absorbance at 450 nm was measured using an enzyme-linked immunosorbent assay reader (BIO-TEK, US) to obtain optical density (OD).

For colony formation assays, cells are seeded in 6-well plates at densities of 300, 500, 1000, 2000, and 4000 cells per well. After 12 hours, the cells were exposed to X-rays at doses of 0, 2, 4, 6, or 8 Gy, respectively. After 14 days of normal culture, the clones formed were observed after fixation with methanol and staining with crystal violet.

### Wound healing assay

Cells were seeded at a density of 1.5×10^5^ in a 24-well plate. When the cell confluence reached approximately 90%, a scratch was made using the tip of a 200 μL pipette. After injury, the cells were washed with PBS to remove debris and then cultured in serum-free medium. Wound healing was imaged every 24 hours using an inverted microscope (Olympus, IX73) for a duration of 48 hours. Image analysis was performed using ImageJ software (V1.8.0.112) for statistical analysis.

### Cell migration and invasion assays

For the cell migration assay, 5×10^4^ cells were seeded in transwell membrane inserts (BD Falcon cat. #353097, NJ, US) without Matrigel (Corning, NYC, US). For the cell invasion assay, 1×10^5^ cells were added to transwell inserts coated with Matrigel. After 24 hours of normal culture, the cells were stained with crystal violet, and images were captured for cell counting.

### Immunofluorescence assay

The assay was conducted following a previously described protocol [35]. DNA damage and repair were assessed using an anti-γ-H2AX (Ser139) antibody (Cell Signaling Cat. # 2577S). The localization of the GPR37 and ATP1A1 proteins in cells overexpressing GPR37 was detected using Flag and ATP1A1 antibodies. Immunofluorescence was performed using Goat anti-Rabbit IgG-AF594 Antibody (Absin cat. #abs20145) and Goat anti-Mouse IgG-AF488 Antibody (Absin cat. #abs20013) secondary antibodies, along with DAPI (Beyotime). Images were captured using a laser confocal microscopy (ZEISS LSM980).

### Flow cytometry

The cells in the experimental group were treated with 8 Gy X-ray radiation, and ESCC cells not subjected to radiation treatment were used as a negative control. After 72 hours, cells were collected and treated by annexin V-FITC/PI Apoptosis Kit (Cat. # AP101C-100; Muiti Sciences). Signal was detected using a Navios flow cytometer (Beckman).

### Detection of DNA methylation levels by bisulfite sequencing PCR

The methylation level of the GPR37 promoter in ESCC cell lines was detected using bisulfite sequencing PCR (BSP). Total genomic DNA was extracted from six ESCC cell lines using a DNAzol reagent kit (Invitrogen, CA, USA) and subsequently identified and quantified using a NanoPhotometer (IMPLEN). Subsequently, the genomic DNA was subjected to bisulfite conversion using the EZ DNA Methylation-Gold Kit (cat. #D5006, ZYMO Research). The following primer sequences were used to amplify the upstream CpG island of GPR37: forward, 5’-GAAGGGTGGTGTTTGGAAT-3’, and reverse, 5’-AATAATAAACCACCAAAAAATCC-3’. The amplification products were cloned and inserted into the T vector, followed by sequencing.

### Exosome extraction

Cells were cultured to 80% confluence in conventional medium, then switched to RPMI-1640 medium without FBS, and after 48 hours, the cell supernatant was collected. Centrifuge the cells at 3000 × g for 30 min at 4 °C. Transfer the supernatant to an ultracentrifuge tube and centrifuge at 16,000 × g for 1 h using an L-100xp ultracentrifuge (Beckman, US) equipped with a model 70 rotor. The supernatant was then centrifuged at 100,000 × g for 1 h, the supernatant was discarded, and the pellet was resuspended in PBS. The resuspended sample was filtered into a 1.5 mL ultracentrifuge tube with a 0.2 µm membrane. Subsequently, the sample was centrifuged at 100,000 × g for 1 hour using an Optima MAX-XP ultracentrifuge (Beckman, USA) equipped with a TLA55 rotor. The supernatant was discarded, and the pellet was resuspended in 20 µL of PBS to obtain the extracellular vesicle sample. The protein concentration of the exosome was determined using the BCA Protein Concentration Assay Kit (Beyotime). The extracellular vesicle preparation can be stored at -80 °C for up to 30 days. The morphology of the exosome was observed using transmission electron microscopy (Tecnai G2 F20). The size distribution of the exosome was measured using a nanoparticle tracking analyzer (PMX220, Particle Metrix, Germany). The expression levels of representative extracellular vesicle markers, such as TSG101 and CD63, were detected via Western blotting.

### In vivo experiments

The mice used in the study were all 4-week-old female BALB/c nude mice purchased from Hangzhou Resources Laboratory Animal Technology Co., Ltd.

For the subcutaneous tumor model, the cells were injected subcutaneously with Matrigel. To investigate the effect of GPR37 on radiosensitivity, vector- or GPR37-overexpressing KYSE150 cells (5×10^6^) were injected subcutaneously in the left hind limb of nude mice (n = 5 per group). Tumor volume is measured using calipers every four days. When tumors reach about 5 mm in diameter, nude mice are randomly grouped (vector, GPR37, vector+8 Gy, and GPR37 + 8 Gy) for X-ray irradiation of 8 Gy (2 Gy/day × 4 days, total 8 Gy, localized irradiation) [36]. After 32 days, the mice were euthanized, and tumor samples were collected for follow-up experiments.

To analyze the effect of Exo-GPR37 on ESCC tumorigenesis, KYSE150 cells (5×10^6^) were subcutaneously injected in the left hind limb of nude mice (n = 3 per group). When tumors reach about 5 mm in diameter, nude mice are randomly grouped (PBS, Exo-vector, Exo-GPR37), and intratumoral injection of Exo or PBS (50 μg) was administered. After 32 days, the mice were euthanized.

For the lung metastasis model, 100 μL of vector- or GPR37-overexpressing KYSE150 cells (1×10^6^) were injected via the tail vein (n = 5 per group). After 9 weeks of injection, the mice were euthanized. The lungs were collected, then fixed in Bouin’s solution (cat. #HT10132, Sigma‒Aldrich) at room temperature for 24 hours. The metastatic lung surface colonies were counted, and H&E stained.

All animal experiments were approved by the Ethics Committee of the Animal Experiment Center of Anhui Provincial Hospital (Approval No: 2022-N(A)-96).

### Silver staining experiments

In the experiment, the proteins were subjected to electrophoresis on a 0.75 mm SDS‒PAGE gel. Then follow the instructions provided with the kit to perform experiments with the Rapid Silver Staining Kit (Beyotime) and take photos.

### Coimmunoprecipitation experiments

After following the instructions provided in the Flag Tag Protein Immunoprecipitation Kit (cat. #P2181S, Beyotime) and HA Tag Protein Immunoprecipitation Kit (cat. #P2185S, Beyotime), the final obtained samples were subjected to mass spectrometry analysis or Western blot detection.

### ChIP‒PCR

Perform experiments using the instructions for the BeyoChIP™ ChIP Assay Kit (Protein A/G Magnetic Beads) (Beyotime cat# P2080S). The primary antibody used was ZNF750. Finally, the extracted nucleic acids were subjected to PCR using the following primer sequences: forward, 5’- GAAGGGTGGTGTTTGGAAT-3’, and reverse, 5’- AATAATAAACCACCAAAAAATCC-3’. The PCR products were then subjected to nucleic acid electrophoresis and visualized using an GEL imager (Tanon 2500).

### Luciferase reporter assay

The wild-type (WT) or mutant (Mut) GPR37 vectors, synthesized by BiooGenetech, were cloned and inserted into the pmiR-RB-ReportTM vector. ZNF750-Vector or ZNF750, along with WT-GPR37 or Mut-GPR37, was cotransfected into cells. After 48 hours, the enzyme activities were analyzed using the Dual-Luciferase Reporter Assay System (Promega).

### Exo uptake experiment

The extracted exosomes (5 μg) were mixed with RPMI-1640 medium (serum-free) and 4 μL PKH26 (Sigma‒Aldrich), incubated for 2 h, and exosomes were re-extracted. Then, co-culture the stained exosomes with the recipient cells for about 30 min. Cells were stained with DAPI and observed by fluorescence microscopy (Olympus).

### Data analysis

All data are expressed as the mean ± standard deviation of at least three independent experiments. Student’s t test and two-tailed chi-squared (χ²) test methods were used to ascertain statistical significance. Spearman’s correlation was used for all correlation analyses. Each independent experiment was replicated at least three times to ensure reliability. All statistical evaluations were conducted utilizing GraphPad (v10) or R (v4.3.0). Statistical significance was inferred for differences where the *p* < 0.05.

## Results

### Low GPR37 expression is associated with worse malignancy, poorer prognosis and radiation resistance in ESCC

**Fig. 1:**
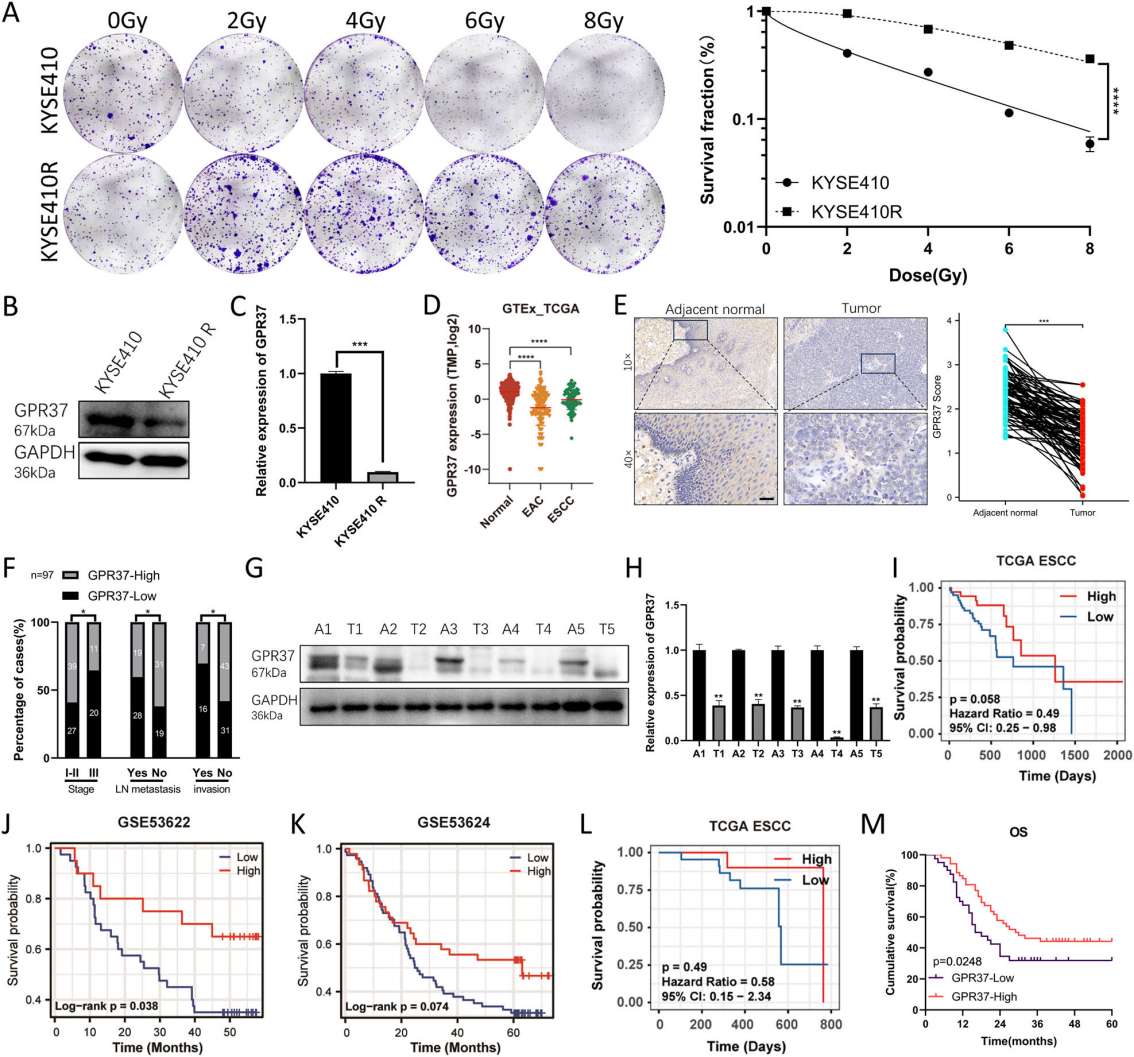
GPR37 is underexpressed in ESCC and is associated with poor prognosis. **A** Colony formation experiments were conducted to test the effectiveness of KYSE410R. The 0 Gy is used as the control group and a single-target multitarget model was used to fit the cell survival curve (*n* = 3). **B** Western blotting was used to detect GPR37 protein levels in KYSE410 and KYSE410R cells. **C** qRT‒PCR was used to detect GPR37 mRNA levels in KYSE410 and KYSE410R cells (*n* = 3). **D** GPR37 is low expressed in ESCC and EAC tissues. **E** Immunohistochemistry was used to analyze the expression level of GPR37 protein in ESCC tissues and adjacent normal tissues (magnification ×10 or ×40), and representative images were displayed; Scale bar: 100 μm. The right panel shows the immunohistochemical staining score for GPR37 in ESCC samples (*n* = 97). **F** Correlation between GPR37 protein levels and clinicopathological parameters of patients with ESCC (*n* = 97). Western blot (**G**) and qRT‒PCR (**H**) were used to detect the expression of GPR37 in fresh ESCC tissues and adjacent normal tissues. Kaplan–Meier analyzed the relationship between OS and GPR37 mRNA level of ESCC patients in TCGA ESCC (**I**), GSE53622 (**J**), and GSE5324 (**K**) datasets. **L** Kaplan–Meier analyzed the relationship between OS and GPR37 mRNA level in ESCC patients receiving radiotherapy in the TCGA database. **M** Kaplan–Meier analysis of the relationship between GPR37 protein expression and overall survival of patients with ESCC. *p < 0.05, **p < 0.01, ***p < 0.001, ****p < 0.0001.

In our previous study, we successfully established the radiation-resistant ESCC cell line KYSE410R from KYSE410 (Fig. 1A and Supplementary Table 1). Subsequent RNA sequencing and differential expression analysis revealed that GPR37 was the most downregulated gene in KYSE410R cells compared to KYSE410 [24]. The significant decrease in GPR37 expression was further confirmed through western blotting and qRT-PCR (Fig. 1B, C).

Furthermore, analyses of GPR37 mRNA expression from TCGA combining with GTEx database showed that GPR37 was significantly reduced in both ESCC and EAC tissues compared to normal esophageal tissues (Fig. 1D). Additionally, low GPR37 mRNA level was correlated with a more advanced T stage, N stage, and pathologic stage in TCGA ESCC (Supplementary Fig. 1). To further corroborate the expression levels of GPR37 in ESCC, immunohistochemistry (IHC) was conducted on ESCC tissues and their adjacent non-tumorous counterparts. The results indicated that GPR37 protein levels were also significantly reduced in ESCC (Fig. 1E). Univariate and multivariate analyses showed that low protein level of GPR37 was linked to tumor stage, lymph node metastasis, and vascular or nerve invasion (Fig. 1F), but not to patient sex, age, or tumor size index (Supplementary Table 2). In addition, western blot and qRT-PCR analyses on five pairs of ESCC and adjacent non-cancerous tissues confirmed that both protein and mRNA expression of GPR37 were significantly lower in cancer tissues (Fig. 1G, H).

Subsequently, an examination into the predictive value of GPR37 expression in ESCC was conducted. Utilizing the Kaplan–Meier Plotter, it was observed that a reduced GPR37 mRNA level correlated with suboptimal overall survival (OS) in patients with ESCC, as evidenced in the TCGA ESCC, GSE53622, and GSE5324 datasets (Fig. 1I–K). Moreover, its low level was linked to a poor OS of ESCC patients who underwent radiotherapy in TCGA ESCC, although this difference was not significant because of small numbers of sample (Fig. 1L). Furthermore, analysis in our own dataset revealed that the reduced protein expression of GPR37 was also associated with shorter OS (Fig. 1M).

These results collectively suggest that GPR37 may function as a putative tumor suppressor gene and could be involved in regulating radiosensitivity of ESCC cells.

### DNA methylation and ZNF750 jointly regulate the expression of GPR37 in ESCC

**Fig. 2:**
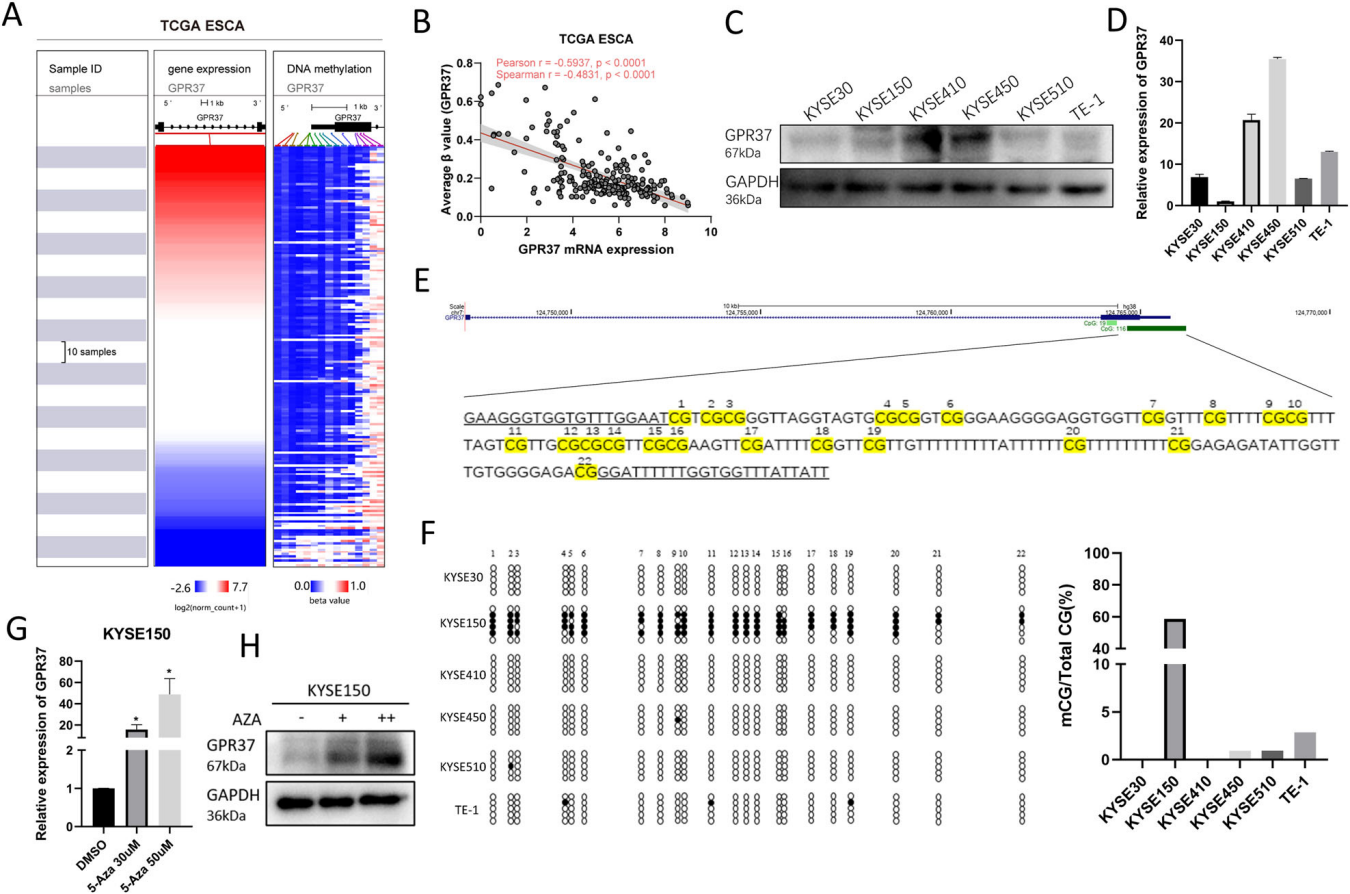
GPR37 expression is inversely regulated by its promoter methylation in ESCC. **A** Heatmap illustrating the mRNA expression levels of GPR37 and the β-values (indicating methylation levels) across 15 CpG sites within the GPR37 promoter region. A total of 194 esophageal cancer samples, which possessed both GPR37 expression data and methylation information, were selected and incorporated into this analysis. The samples are arranged in ascending order based on GPR37 mRNA expression, from the top to the bottom of the heatmap. Each horizontal row represents an individual sample. Blue: low level; Red: high level. **B** Correlation of mean methylation levels of all CpG sites in the GPR37 promoter region with GPR37 mRNA levels in TCGA ESCA. **C** Western blotting analysis was employed to ascertain the protein expression levels of GPR37 across six ESCC cell lines. **D** qRT-PCR was utilized to quantitatively measure the mRNA expression levels of GPR37 in these same six ESCC cell lines (*n* = 3). **E** The ESCC genome browser shows the location of the GPR37 gene and its upstream region. Twenty-two CpG sites within the CpG island in the promoter region of GPR37 were analyzed via bisulfite sequencing PCR (BSP). **F** The methylation status of CpG sites in six ESCC cell lines was detected by BSP. Methylated CpG sites are represented by packed circles, and unmethylated CpG sites are represented by hollow circles. Effect of 5-AZA treatment on the mRNA (**G**) and protein (**H**) levels of GPR37 in KYSE150 cells (*n* = 3). *p < 0.05.

Subsequently, we investigated the possible mechanisms underlying the reduced expression of GPR37 in ESCC. We and others have elucidated that promoter DNA hypermethylation constitutes a pivotal epigenetic mechanism involved in the suppression of gene expression [37, 38]. An integrative analysis of both transcriptional and methylation sequencing data from TCGA ESCA cohort revealed that the methylation levels of the predominant CpG sites (14/15) in the GPR37 promoter region, including cg17052813, cg26141626, cg07392724, cg16847696, cg17152484, cg14311320, cg01667837, cg23428445, cg09458673, cg22230167, cg02960853, cg27533119, cg23799901 and cg26278103, was significantly inversely correlated with the corresponding mRNA levels of GPR37 (Fig. 2A and Supplementary Fig. 2). Moreover, the mean methylation levels across all these 15 CpG sites exhibited a significant negative correlation with GPR37 expression (Pearson *r* = −0.5937, *p* < 0.001, Fig. 2B). To corroborate the insights gleaned from our bioinformatic analysis, we conducted experimental validations to assess the expression level of GPR37 and methylation level of its promoter across six ESCC cell lines. The protein and mRNA expression of GPR37 was high in KYSE450 and KYSE410 cells, in contrast to its minimal expression in KYSE150 cells (Fig. 2C, D). While BSP-PCR analysis revealed that GPR37 promoter was hypomethylated in KYSE450 and KYSE410 cells, in contrast to the hypermethylated state observed in KYSE150 cells (Fig. 2E, F). Furthermore, treatment of KYSE150 cells with the DNA methylation inhibitor 5-Aza led to a dose-dependent increase in both the RNA and protein expression of GPR37 (Fig. 2G, H). These experimental findings collectively support the hypothesis that DNA methylation serves as a negative regulator of GPR37 expression in ESCC.

**Fig. 3:**
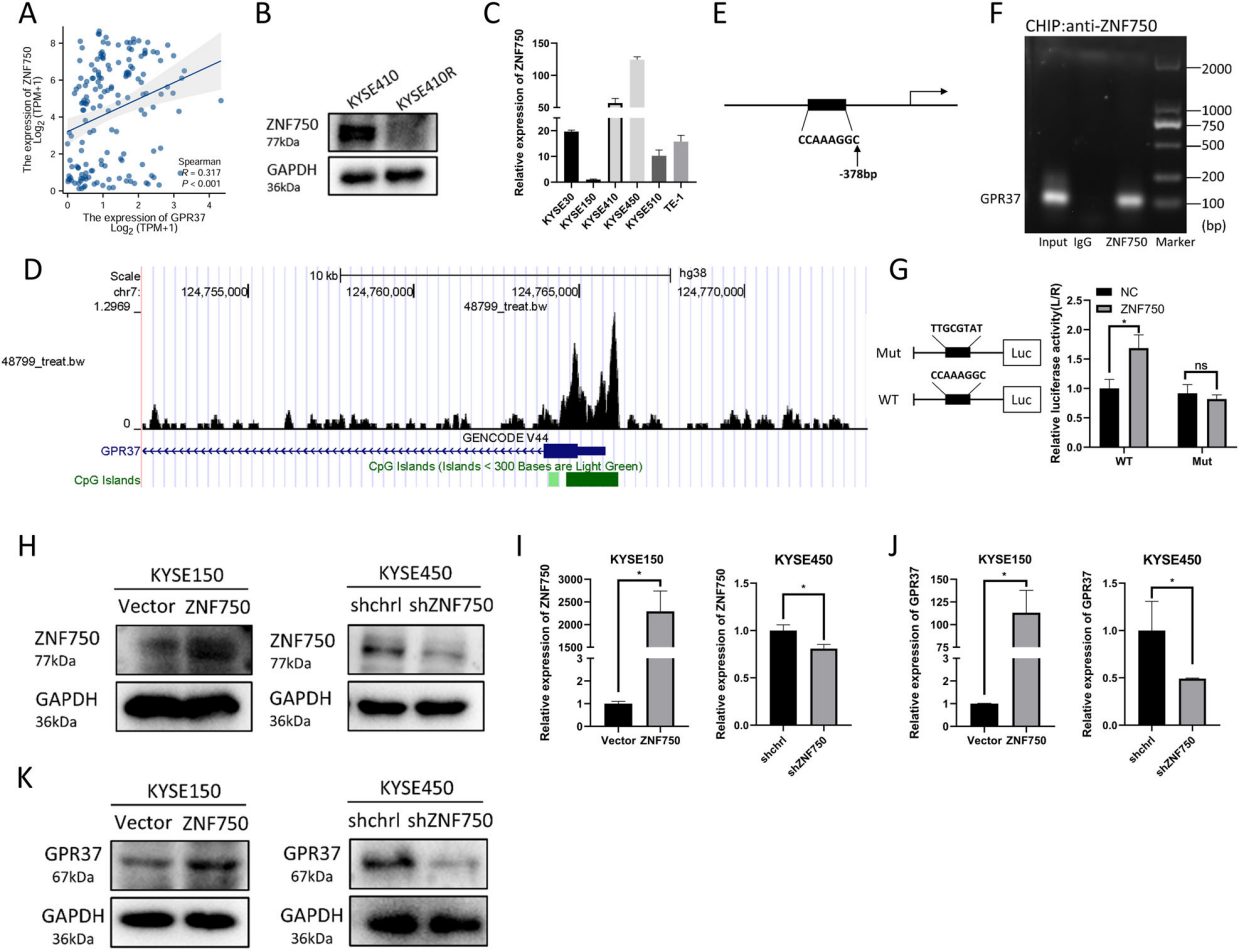
GPR37 transcription is regulated by ZNF750 in ESCC. **A** Correlation between ZNF750 expression and GPR37 expression in ESCA. **B** Western blot analysis of ZNF750 levels in KYSE410 and KYSE410R cells. **C** qRT‒PCR was used to detect the expression of ZNF750 in six ESCC cell lines (*n* = 3). **D** ZNF750 interacts with GPR37 promoter region. **E** Location of the binding site between ZNF750 and GPR37 DNA. **F** ChIP experiments showed that ZNF750 was enriched in GPR37. **G** Luciferase reporter gene detection suggested that GPR37 interacts with ZNF750. Western blotting (**H**) was used to detect the protein expression level of ZNF750, and qRT‒PCR (**I**) was used to detect the mRNA expression level of ZNF750 (*n* = 3). **J** qRT‒PCR was used to detect the expression level of the GPR37 mRNA (*n* = 3). **K** Western blotting was used to detect the protein expression level of GPR37 in cells after ZNF750 overexpression. ns not significant, *p < 0.05.

Our previous study showed that the transcription factor ZNF750 was also significantly decreased in esophageal cancer [38]. Correlation analysis conducted on TCGA ESCA dataset revealed a significant positive correlation between ZNF750 and GPR37 expression levels (Fig. 3A). Furthermore, analysis of the protein expression in the KYSE410R cell line revealed that ZNF750 levels were reduced compared to those in the wild-type cells. (Fig. 3B), which was in line with the trend of expression changes of GPR37 (Fig. 3B). Therefore, we hypothesized that ZNF750 might regulate GPR37 expression. In consonance with our hypothesis, the high and low order of ZNF750 expression in the six esophageal cancer cell lines was consistent with GPR37 (Figs. 2D and 3C). To delve deeper into the molecular interaction, we conducted an additional analysis using ChIP-seq data of ZNF750 from a previous study [39], which revealed that ZNF750 bind to the GPR37 promoter (Fig. 3D). Notably, this binding region basically coincides with the CpG island region (Fig. 3D). Previous studies have identified CCNNAGGC sequence as a ZNF750 binding motif [39, 40]. Analysis of the GPR37 promoter sequence revealed the presence of such a motif, located 378 bp upstream of its transcription start site (TSS) (Fig. 3E). A subsequent ChIP experiment confirmed the specific binding of ZNF750 to this site (Fig. 3F). Additionally, dual luciferase reporter assays employing the GPR37 promoter with either a WT binding site or a mutant binding site showed that ZNF750 increased luciferase activity specifically in the context of the wild-type promoter, with no such effect observed in the mutant promoter (Fig. 3G). Moreover, ZNF750 overexpression significantly elevated the mRNA and protein levels of GPR37 in KYSE150 cells, while ZNF750 knockdown suppressed GRP37 expression in KYSE450 cells (Fig. 3H–K and Supplementary Fig. 3A–D).

Taken together, these results lead us to conclude that GPR37 expression in ESCC is negatively regulated by DNA methylation and positively regulated by the transcription factor ZNF750.

### GPR37 enhances the radiation sensitivity of ESCC cells

Next, we investigated the potential functions of GPR37 in the progression of ESCC. GSEA using the Hallmark gene sets indicated a substantial negative association between the GPR37 expression level and the “DNA Repair”, “Reactive oxygen species pathway”, as well as the “UV response UP” signatures in TCGA ESCC (Supplementary Fig. 4A–C). Combined with the result that GPR37 was significantly reduced in radiation-resistant cell lines (Fig. 1B), we hypothesized that GPR37 deficiency is likely to promote radiation tolerance in ESCC cells and investigated the influence of GPR37 on ESCC radiosensitivity.

**Fig. 4:**
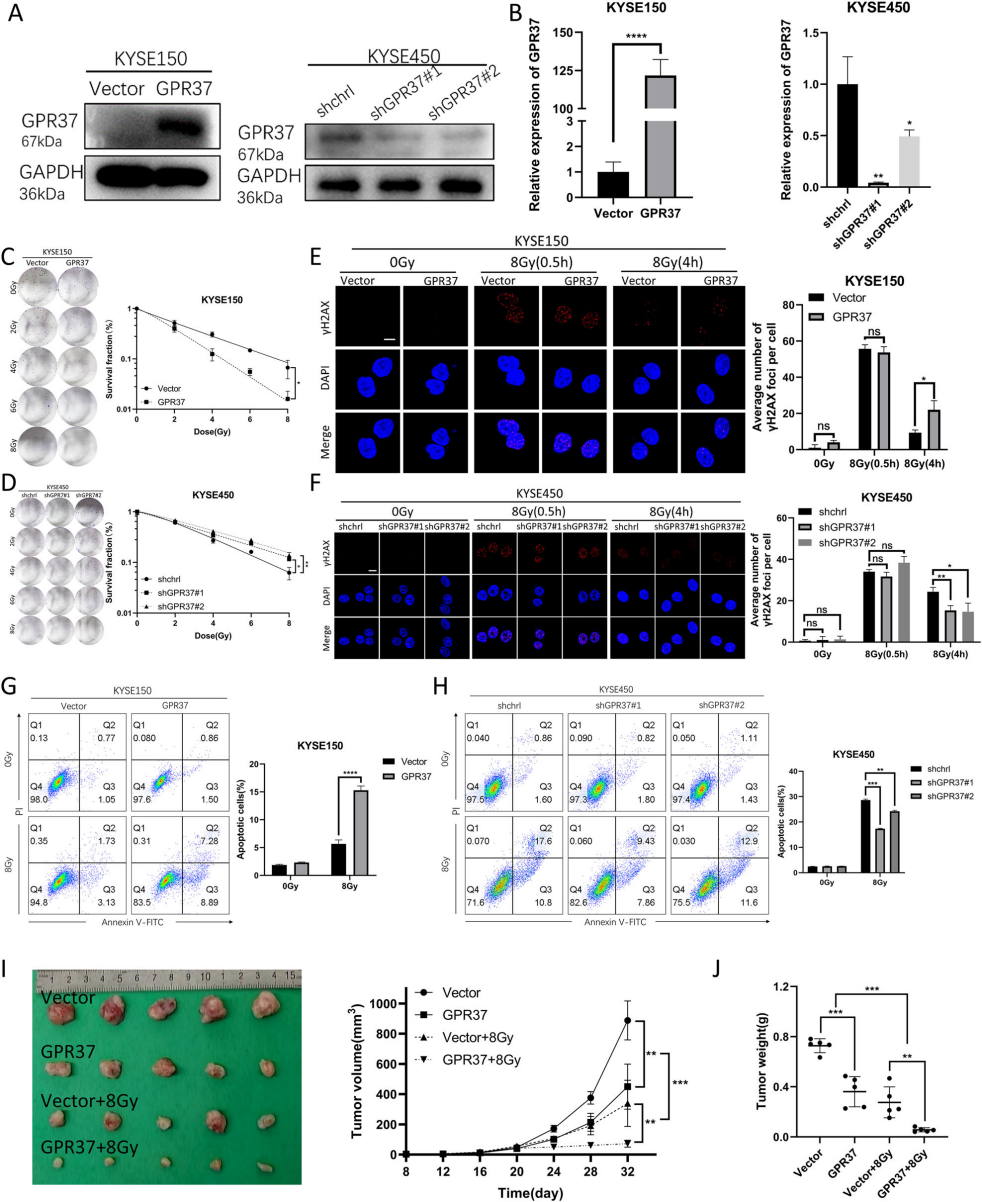
Effect of GPR37 overexpression and silencing on radioresistance of ESCC cells. **A** Western blotting was used to detect the protein expression of GPR37. **B** qRT‒PCR was used to detect the mRNA expression level of the genes (*n* = 3). KYSE150 (**C**) and KYSE450 (**D**) cells were exposed to different radiation doses (0, 2, 4, 6, and 8 Gy, 2 Gy/min) and cultured for 14 days, after which colony formation was assessed to determine their radiosensitivity. The 0 Gy is used as the control group and a single-target multitarget model was used to fit the cell survival curve (*n* = 3). After 8 Gy X-ray treatment, immunofluorescence was used to detect the lesion density of γ-H2AX in KYSE150 (**E**) and KYSE450 (**F**) cells at 0.5 h and 4 h, and the 0 Gy group is used as the control group (*n* = 3). Scale bar: 10 µm. Apoptosis in KYSE150 (**G**) and KYSE450 (**H**) cells after 8 Gy irradiation was assessed by flow cytometry (*n* = 3). **I**, **J** KYSE150 cells overexpressing GPR37 were injected subcutaneously into the hind limbs of BALB/c mice (*n* = 5 per group). **I** Transplanted tumors (tumor volume measured every 4 days) and (**J**) tumor weight. ns not significant, *p < 0.05, **p < 0.01, ***p < 0.001, ****p < 0.0001.

We then established KYSE150 cell lines with-overexpression of GPR37 and a KYSE450 cell line with GPR37 knockdown, confirmed through western blotting (Fig. 4A and Supplementary Fig. 4D) and qRT‒PCR (Fig. 4B and Supplementary Fig. 4E). Colony formation assays demonstrated that GPR37 overexpression markedly reduced the survival fraction of KYSE150 cells, while GPR37 knockdown elevated the survival rate of KYSE450 cells after radiation exposure (Fig. 4C, D and Supplementary Fig. 4F). The Sensitization Enhancement Ratios (SER) for GPR37-overexpressing KYSE150 cell was 1.855 (Supplementary Table 3), while for the KYSE450 cells with shGPR37#1 and shGPR37#2 constructs, the SERs were determined to be 0.645 and 0.656, respectively (Supplementary Table 4). We then used immunofluorescence staining to assess γ-H2AX foci formation, a sensitive marker of DNA damage, following 8 Gy radiation. The γ-H2AX foci formed promptly and culminated at 30 min after radiation treatment (RT), with a subsequent decline observed at 4 hours in wild-type KYSE150 cells. However, in GPR37-overexpressing KYSE150 cells, the γ-H2AX foci remained prominent at 4 hours, indicating prolonged DNA damage (Fig. 4E and Supplementary Fig. 4G). Consistently, γ-H2AX fluorescence intensity was markedly reduced compared to that of the control cells at the 4-hour point post-irradiation (Fig. 4F). This suggested that GPR37 delayed DNA repair processes and thereby enhanced radiosensitivity of ESCC cells. Additionally, GPR37 overexpression in KYSE150 cells increased apoptosis rates following radiation (Fig. 4G and Supplementary Fig. 4H), while GPR37 knockdown in KYSE450 cells decreased apoptosis rates post-radiation, as evidenced by flow cytometry (Fig. 4H). These data indicate that GPR37 increases the radiosensitivity of ESCC cells in vitro. To explore the potential of GPR37 to modulate radiosensitivity of ESCC in vivo, KYSE150-derived xenograft models in nude mice were created. The tumors in the group with GPR37 overexpression exhibited significantly reduced volumes and weights compared to the control mice, particularly after exposure to radiation (Fig. 4I, J), indicating that GPR37 also significantly elevates the radiosensitivity of ESCC in vivo.

### GPR37 suppresses the proliferative, migratory, and invasive capabilities of ESCC cells

**Fig. 5:**
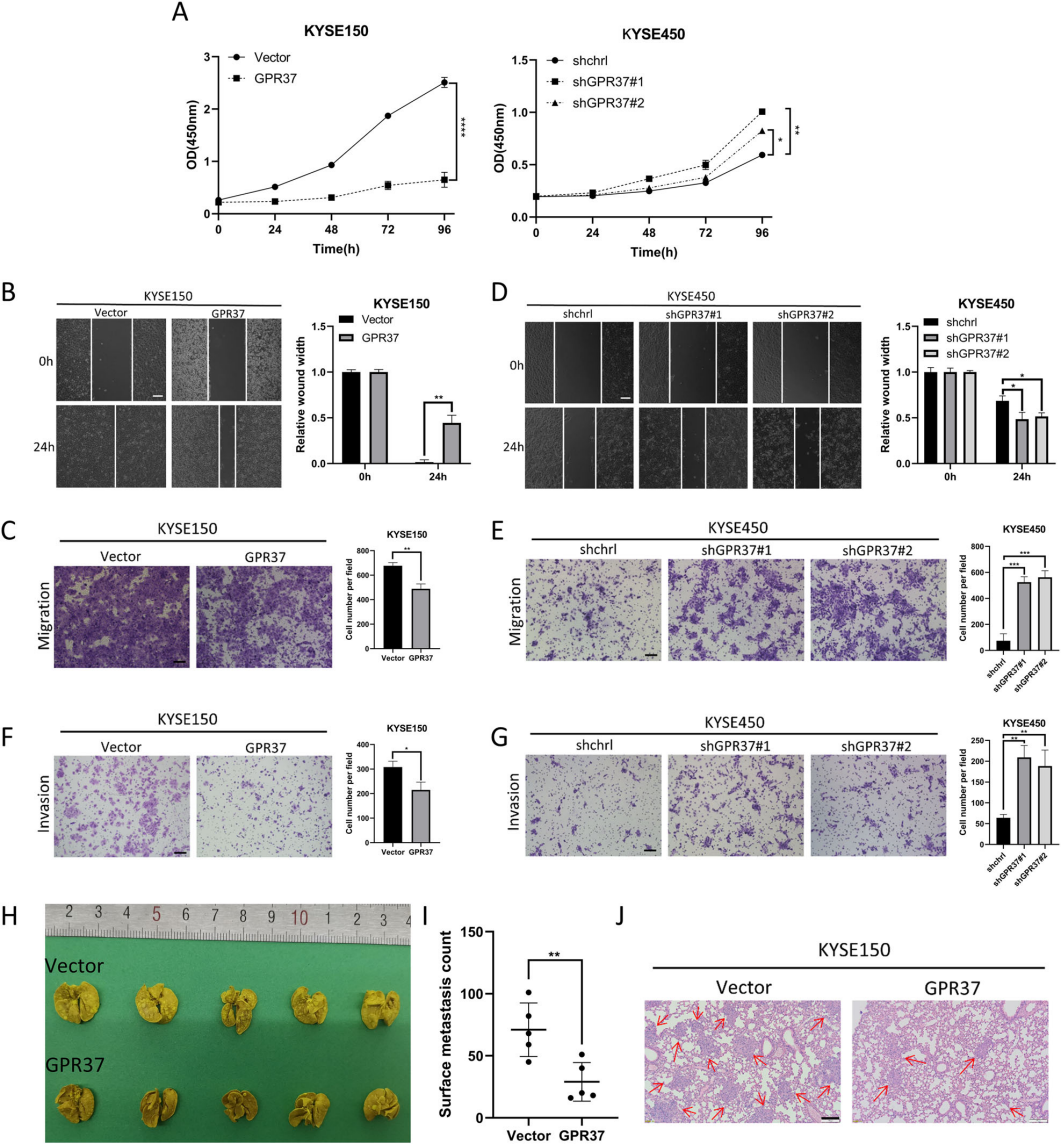
Effects of GPR37 overexpression and knockdown on ESCC proliferation, migration, and invasion. **A** A CCK8 assay was used to evaluate the proliferation of GPR37-overexpressing cells and GPR37-knockdown cells (*n* = 3). A wound healing assay (**B**) and a transwell assay without Matrigel (**C**) were used to detect the migration of ESCC cells overexpressing GPR37, respectively (*n* = 3). The wound width at 0 h was normalized. Scale bar: 100 μm. A wound healing assay (**D**) and a transwell assay without Matrigel (**E**) were used to detect the migration of ESCC cells lacking GPR37, respectively (*n* = 3). The wound width at 0 h was normalized. Scale bar: 100 μm. Determination of invasion of ESCC cells overexpressing (**F**) and lacking (**G**) GPR37 by the Matrigel-containing transwell method. (*n* = 3). Scale bar: 100 μm. **H**, **I** KYSE150 cells overexpressing KYSE150 or GPR37 were injected into the tail vein of BALB/c mice (*n* = 5 per group). **H** Representative images of the lungs were taken 9 weeks after injection. **I** Quantitative analysis of lymph node metastases on the lung surface (*n* = 5). **J** Representative photograph of a lung section showing H&E staining. Scale bar: 100 µm. *p < 0.05, **p < 0.01, ***p < 0.001, ****p < 0.0001.

GSEA in TCGA ESCC also revealed a significant inverse correlation between GPR37 expression levels and key signatures associated with “G2M checkpoint”, “Glycolysis”, “Epithelial mesenchymal transition”, and “Angiogenesis” (Supplementary Fig. 5A–D). These findings imply that ESCC tissues expressing lower levels of GPR37 may experience increased cell proliferation, invasion, and metastasis. Functionally, CCK-8 assays demonstrated that KYSE150 cells with GPR37 overexpression exhibited a notable reduction in proliferation, whereas KYSE450 cells with GPR37 knockdown showed an increase in proliferative activity (Fig. 5A and Supplementary Fig. 5E). Wound-healing assays showed that the rate of wound healing was slower in GRP37-overexpressing KYSE150 cells than in control cells (Fig. 5B and Supplementary Fig. 5F). In accordance with this result, the transwell migration assay revealed decreased cell migration when GPR37 was overexpressed (Fig. 5C and Supplementary Fig. 5G). In contrast, knockdown of GPR37 enhanced the migratory capacity of KYSE450 cells (Fig. 5D, E). Moreover, GPR37 also significantly reduced the invasion abilities of ESCC cells, which was demonstrated by transwell invasion assays (Fig. 5F, G and Supplementary Fig. 5H). Furthermore, our observations also pointed to a notable upsurge in the protein expression of E-cadherin, concurrent with a downturn in the expression of N-cadherin, upon GPR37 overexpression. In addition, GPR37 also significantly suppressed Slug expression, a key transcriptional factor that drives EMT (Supplementary Fig. 5I). This indicates that GPR37 could inhibit EMT, subsequently inhibiting cell migration and invasion of ESCC.

Subsequently, to assess the in vivo impact of GPR37 on the metastasis of ESCC, we employed a lung metastasis model. We randomly allocated 10 Balb/c nude mice into two groups, with each group receiving either KYSE150 cells or KYSE150 cells with GPR37 overexpression via tail vein injections, with five mice per group. After nine weeks, we counted and analyzed the visible lung metastatic nodules. The data presented in Fig. 5H, I revealed a marked reduction in the number of metastatic nodes in the lungs of mice that received the GPR37-overexpressing KYSE150 cells. Hematoxylin and eosin (H&E) staining of the lung metastatic lesions provided further confirmation of these findings (Fig. 5J).

Collectively, the cumulative evidence suggests that GPR37 serves as a suppressor of ESCC cell progression.

### GPR37 functions by inhibiting the AKT/mTOR signaling pathway

**Fig. 6:**
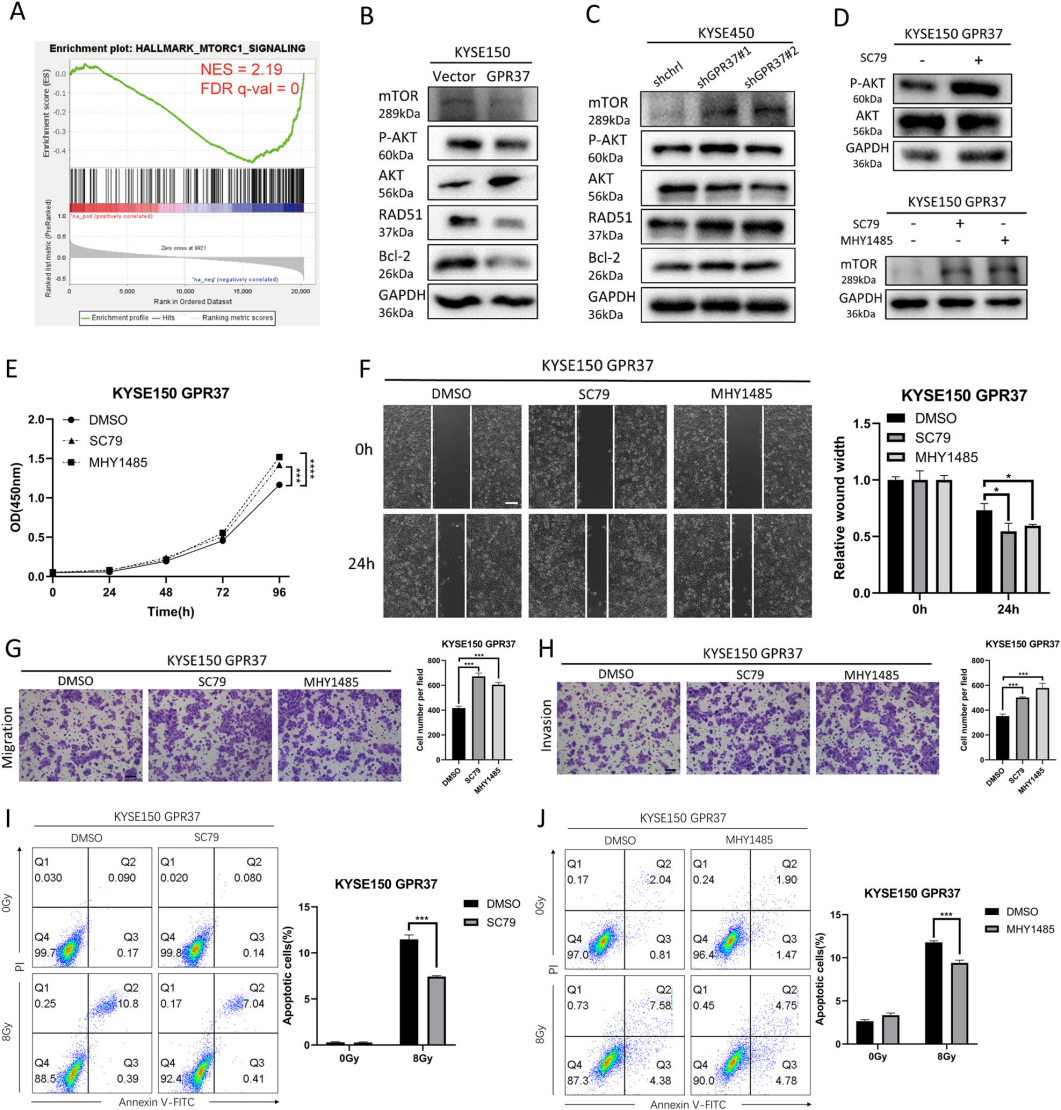
GPR37 affects ESCC function and radiation tolerance by affecting the AKT/mTOR signaling pathway. **A** The GSEA results showed that GPR37 was negatively correlated with the mTOR signaling pathway. Western blotting was used to measure the levels of AKT/mTOR-related proteins in KYSE150 (**B**) and KYSE450 (**C**) cells. **D** Western blotting was used to detect changes in the KYSE150 cell protein after drug treatment. **E** A CCK8 assay was used to evaluate the proliferation of KYSE150 cells after drug treatment (*n* = 3). **F** Cell migration was measured after drug treatment of KYSE150 cells using a wound healing assay (*n* = 3). The wound width at 0 h was normalized. Scale bar: 100 μm. **G** Cell migration was measured after drug treatment of KYSE150 cells using a transwell assay without Matrigel (*n* = 3). Scale bar: 100 μm. **H** Transwell assays with Matrigel were used to measure the invasion of KYSE150 cells after drug treatment (*n* = 3). Scale bar: 100 μm. Flow cytometry was used to evaluate the apoptosis of SC79 (**I**)- and MHY1485 (**J**)-treated KYSE150 cells after 8 Gy irradiation (*n* = 3). ns not significant. *p < 0.05, ***p < 0.001, ****p < 0.0001.

Our subsequent efforts were aimed at delving deeper into the molecular mechanisms that GPR37 engages with to modulate the progression and radiosensitivity of esophageal cancer. GSEA analysis of TCGA ESCA data suggested that GPR37 expression significantly negatively correlated with the mTOR signaling pathway (Fig. 6A). GPR37 overexpression in KYSE150 led to decreased levels of mTOR and P-AKT, which is an upstream activator of mTOR (Fig. 6B and Supplementary Fig. 6A). While GPR37 knockdown in KYSE450 resulted in increased levels of mTOR and P-AKT (Fig. 6C). To validate that GPR37 indeed functions through AKT/mTOR pathway, we treated GPR37-overexpressing KYSE150 cells with the AKT activator SC79 and the mTOR activator MHY1485 (Fig. 6D and Supplementary Fig. 6B). Both SC79 and MHY1485 enhanced the proliferation, wound healing, migration, and invasion of KYSE150 and KYSE510 cells (Fig. 6E–H and Supplementary Fig. 6C–F).

Furthermore, both activators reduced the proportion of radiation-induced apoptotic cells in GPR37-overexpressing KYSE150 cells (Fig. 6I, J and Supplementary Fig. 6G, H). Additionally, we treated GPR37-knockdown KYSE450 cells using the AKT inhibitor MK2206 and the mTOR inhibitor TORIN1 (Supplementary Fig. 7A) and found that both these inhibitors impaired cell proliferation, wound healing, migration, invasion, and increased the proportion of radiation-induced apoptotic cells in GPR37-knockdown KYSE450 cells (Supplementary Fig. 7B–F). Collectively, the evidence gathered supports the notion that GPR37 modulates the progression and radiosensitivity of ESCC by targeting the AKT/mTOR signaling pathway.

### GPR37 suppresses the AKT/mTOR signaling pathway by promoting ubiquitination of ATP1A1

**Fig. 7:**
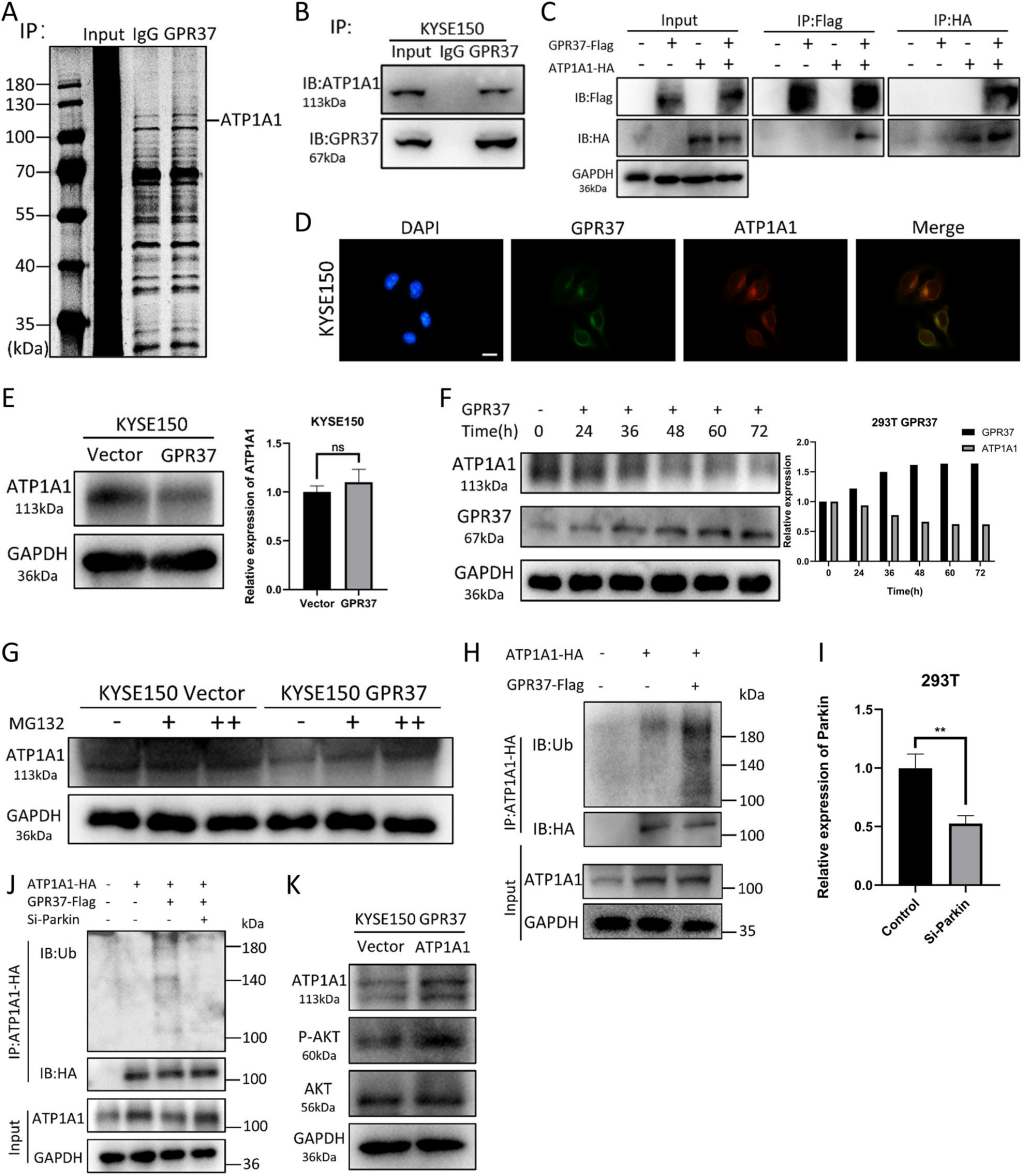
GPR37 promotes ubiquitinated degradation of ATP1A1. **A** Silver staining showing a sample of GPR37 co-IP. **B** Western blot analysis of endogenous Co-IP samples. **C** Western blot analysis of exogenous Co-IP samples. **D** Immunofluorescence detection of the location of GPR37 and ATP1A1 in KYSE150 cells. Scale bar: 10 μm. **E** Western blotting and qRT-PCR were used to detect the expression level of ATP1A1 in KYSE150 cells (*n* = 3). **F** Western blotting was used to detect the changes in the levels of GPR37 and ATP1A1 protein in 293 T cells after transfection with GPR37 overexpression plasmid, and the relative protein expression levels were statistically analyzed compared with the control group (0 h). **G** Western blotting was used to detect the ATP1A1 protein level after MG132 treatment. **H** The ubiquitination level of ATP1A1 in cells with different GPR37 expression levels was detected. **I** qRT-PCR was used to detect the mRNA expression level of Parkin (*n* = 3). **J** Western blotting was used to detect the ubiquitination level of ATP1A1 in cells after co-transfection of different plasmids. **K** Western blotting was used to detect the protein levels in KYSE150 cells overexpressing ATP1A1. **p < 0.01.

In our quest to decipher the molecular underpinnings by which GPR37 represses the AKT/mTOR signaling pathway, we engaged in a search for potential interacting proteins of GPR37 in ESCC cells. Immunoprecipitation coupled with mass spectrometry (IP/MS) assays using an anti-GPR37 antibody in GPR37-overexpressing KYSE150 cell lines were employed (Supplementary Table 5). It identified ATP1A1 as a putative protein that may interact with GPR37 (Fig. 7A). Subsequent validation via western blotting confirmed this interaction (Fig. 7B and Supplementary Fig. 8A). To further substantiate this finding, 293 T cells were cotransfected with GPR37-Flag and ATP1A1-HA plasmids, followed by a Co-IP assay using anti-Flag or anti-HA antibodies, which corroborated the exogenous interaction between GPR37 and ATP1A1 (Fig. 7C). Additionally, immunofluorescence staining revealed the colocalization of GPR37 and ATP1A1 in cells (Fig. 7D and Supplementary Fig. 8B), providing further evidence of their interaction.

Next, we observed that the overexpression of GPR37 led to a reduction in the protein levels of ATP1A1, while leaving its mRNA expression unaffected (Fig. 7E and Supplementary Fig. 8C). The GPR37 overexpression plasmid was transiently transfected into 293 T cells, and it was found that the level of ATP1A1 protein decreased gradually with the increase of GPR37 expression (Fig. 7F).

To ascertain whether the reduction in ATP1A1 protein levels was related to proteasomal degradation, we treated KYSE150 control cells and those overexpressing GPR37 with the proteasome inhibitor MG132. This treatment effectively restored ATP1A1 protein levels in both cell types (Fig. 7G and Supplementary Fig. 8D), suggesting that the proteasome pathway plays a role in the regulation of ATP1A1 protein stability. Furthermore, the ubiquitination level of ATP1A1 was detected, and it was found that the ubiquitination level of ATP1A1 increased with the increase of GPR37 expression (Fig. 7H). These results suggest that GPR37 binds ATP1A1 and facilitates its degradation.

Previous studies have established that GRP37 can interact directly with the E3 ubiquitin-protein ligase parkin [9]. Therefore, we hypothesized that GRP37 could promote ubiquitination as well as degradation of the adjacent ATP1A1 protein by recruiting parkin. To test this hypothesis, we performed the following experiments. We first transfected 293 T cells with GPR37-Flag, ATP1A1-HA, and siRNA knockdown of Parkin, and then detected the changes in the expression and ubiquitination levels of ATP1A1 proteins by Co-IP assay and western blotting. The results showed that the protein level of ATP1A1 decreased and its ubiquitination level increased after the increase of GPR37 expression (Fig. 7H–J). In contrast, Parkin knockdown in cells with increased GPR37 expression reversed this effect (Fig. 7I, J). These results validate our hypothesis and reveal a mechanism of ubiquitination degradation regulation of ATP1A1 by GPR37 through recruitment of Parkin.

ATP1A1 is a functional subunit of Na+/K+ ATPase. Previous research has established that activating Na+/K+ ATPase can inhibit the accumulation of ROS, and the activation of Na+/K+ ATPase or ATP1A1 overexpression can activate Akt/mTOR signaling pathways [41, 42]. Actually, we found that ATP1A1 overexpression resulted in increased levels of phosphorylated AKT in ESCC cells (Fig. 7K and Supplementary Fig. 8E). Collectively, these findings indicate that GPR37 could inhibit the activation of the AKT/mTOR signaling pathway by interacting with Parkin to promote ubiquitination of ATP1A1.

### Exo-GPR37 can influence the behavior and radiosensitivity of ESCC cells

Considerable research has highlighted the pivotal role of exosomes in the pathogenesis of ESCC [29, 43]. Moreover, it has recently been reported that exosomes can modulate inflammatory responses through the GPR37 pathway [13]. Prompted by these findings, we endeavored to explore the possible involvement of GPR37 in the exosomal pathway and its possible role in mediating exosome-mediated effects in ESCC.

**Fig. 8:**
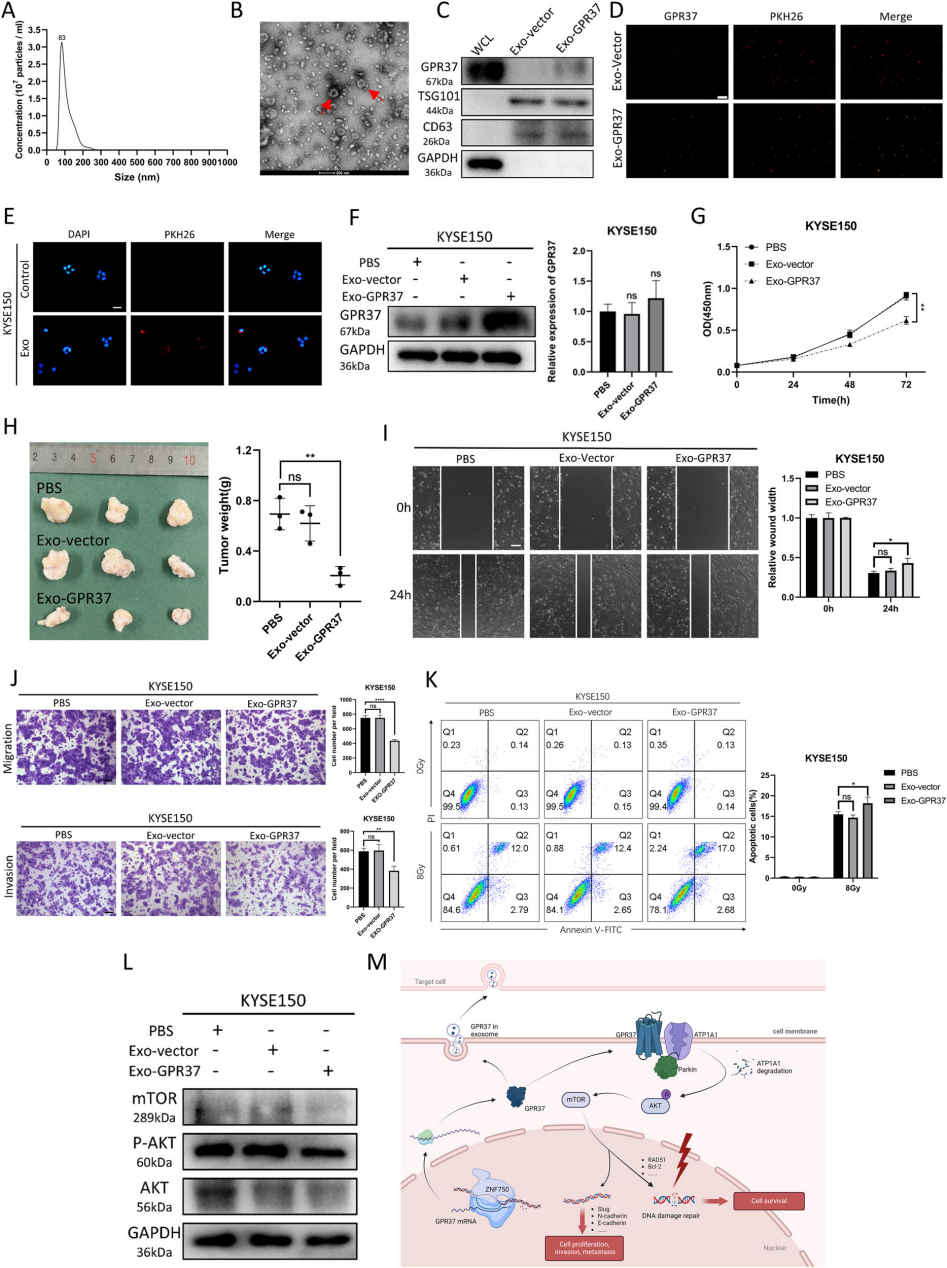
Effect of exosome delivery of GPR37 on ESCC cell function and the radiation response. **A** Nanoparticle tracking analysis was used to determine the size of the exosomes. **B** TEM image showing the morphology of the exosomes. Scale bar, 200 nm. **C** Western blotting showed that GPR37 and the exosomal protein markers CD63 and TSG101 were enriched in E-cell exosomes secreted by KYSE150 cells. **D** Immunofluorescence showed that GPR37 protein was carried by exosomes. Scale bar: 100 μm. **E** An exosome uptake assay showed that PKH26-labeled exos were taken up by KYSE150 cells. Scale bar: 100 μm. **F** Western blotting (left) and qRT-PCR (right) were used to detect the GPR37 expression level in KYSE150 cells after coincubation with Exo-vector or Exo-GPR37 (*n* = 3). **G** The proliferation of KYSE150 cells coincubated with Exo-vector or Exo-GPR37 was determined via the CCK-8 method (*n* = 3). **H** Representative images of tumors from BALB/c mice (left) and tumor masses (right). (*n* = 3 per group). Wound healing assays (**I**) and transwell assays (**J**) were used to detect the migration and invasion of KYSE150 cells after coincubation with Exo-vector and Exo-GPR37 (*n* = 3). The wound width at 0 h was normalized. Scale bar: 100 μm. **K** Flow cytometry was used to evaluate the radiosensitivity of KYSE150 cells after coincubation with the EV and Exo-GPR37 (*n* = 3). **L** Western blotting was used to detect the expression level of AKT/mTOR-related proteins in KYSE150 cells after coincubation with Exo-vector or Exo-GPR37. **M** Schematic diagram of this study. ns: not significant. ns not significant, *p < 0.05, **p < 0.01, ***p < 0.001, ****p < 0.0001.

Exosomes were separated from the culture medium of ESCC cells through differential centrifugation, followed by their characterization using Nanoparticle tracking analysis (NTA) and transmission electron microscopy (TEM). These analyses revealed that the exosomes exhibited a typical spherical shape, with an average size of ~100 nm in diameter (Fig. 8A, B and Supplementary Fig. 9A, B). And, western blotting confirmed the presence of CD63 and TSG101, two positive markers of exosomes, in the isolated exosomes (Fig. 8C and Supplementary Fig. 9C), thereby verifying the successful collection of exosomes. Western blot results showed that exosomes derived from cells with high GPR37 expression also had higher levels of GPR37 (Fig. 8C and Supplementary Fig. 9C). Further, GPR37 was observed to be carried in large quantities by Exo-GPR37 by immunofluorescence experiments (Fig. 8D). To ascertain if the exosomes could be absorbed by ESCC cells, the exosomes were first tagged with the red fluorescent dye PKH26, followed by co-culture with the ESCC cells. Subsequent fluorescence microscopy observations validated that the KYSE150 cells had indeed ingested and internalized the exosomes (Fig. 8E and Supplementary Fig. 9D). Furthermore, co-culture with Exo-GPR37 led to elevated levels of GPR37 in the recipient esophageal cancer cells, with no corresponding change in RNA levels (Fig. 8F and Supplementary Fig. 9E).

The co-culture with Exo-GPR37 inhibited the proliferation rate of ESCC cells (Fig. 8G and Supplementary Fig. 9F). Xenograft tumor assay demonstrated that Exo-GPR37 could also inhibit ESCC proliferation in vivo (Fig. 8H). The wound healing (Fig. 8I and Supplementary Fig. 9G), transwell (Fig. 8J and Supplementary Fig. 9H), and apoptosis (Fig. 8K and Supplementary Fig. 9I) experiments indicated that Exo-GPR37 suppressed the migration, invasion of ESCC cells and enhanced their radiosensitivity, respectively. Western blot results showed that Exo-GPR37 exerted its effect on ESCC cells through the AKT/mTOR signaling pathway (Fig. 8L and Supplementary Fig. 9J). Furthermore, we conducted a co-culture experiment where exosomes labeled with PKH26 were incubated with HEEC cells. Our findings indicated that the internalization efficiency of exosomes by HEEC cells was substantially lower compared to that observed in ESCC cells (Supplementary Fig. 9K). We also assessed the proportion of HEEC apoptosis following radiotherapy and discovered that Exo-GPR37 had a negligible impact on HEEC, with no statistically significant effect (Supplementary Fig. 9L). These findings implicate GPR37 in the regulation of protein levels within recipient cells via exosomal mediation, which in turn has the potential to modulate the tumor phenotype. This insight suggests that augmenting the therapeutic potential of exosomes could represent a viable strategy to enhance the efficacy of treatments for ESCC.

## Discussion

Comprehensive treatment for ESCC patients typically includes surgical resection, chemotherapy, and radiotherapy [3]. Radiotherapy is among the most effective treatment modalities [44, 45]. In recent studies, various targeted drugs and neoadjuvant chemoradiotherapy approaches have been used for ESCC treatment [46, 47]. However, ESCC patients have low survival rates and high recurrence rates, and one of the main reasons is radiation tolerance [48]. Therefore, it is crucial to discover new potential molecular targets to enhance radiosensitivity and improve the therapeutic efficacy of treatment for ESCC. In this study, we identified GPR37 as an important radiosensitive target.

GPR37 plays an important role in various diseases [20, 49]. Previous studies have shown that GPR37 can interact with CDK6, influence the tumor progression of lung adenocarcinoma [18], and promote metastasis in colorectal cancer through the Hippo pathway [19]. However, research on GPR37 in esophageal cancer is currently lacking. Therefore, we investigated the biological roles of GPR37 in esophageal cancer. Our findings revealed that GPR37 was downregulated in ESCC tissues when compared to adjacent normal tissues, and its reduced expression was correlated with an unfavorable prognosis. Multiple reports have shown that GPR37 can promote tumor development [17–19], while other studies have indicated its inhibitory effect on liver cancer [22]. These findings suggest that GPR37 may play different roles in different types of cancer. This differential gene function in various tumors may result from genetic heterogeneity, diverse epigenetic regulations, and distinct signaling pathway activations. Tumor-specific genetic backgrounds, epigenetic modifications (e.g., DNA methylation, histone modifications, non-coding RNA expression), and environmental influences contribute to the varied roles of a gene across tumor types. In our research, we were the first to showcase the anticancer properties of GPR37 in ESCC. Our data indicated that elevated GPR37 expression suppressed esophageal cancer cell proliferation, invasion, and migration, whereas decreased GPR37 expression conversely enhanced these cellular processes. Previous reports have shown that GPR37 is associated with resistance to certain drugs, such as cisplatin [17], suggesting that GPR37 may affect DNA damage repair. In our research, we provided the first evidence that GPR37 enhances the radiosensitivity of ESCC through experiments involving colony formation assays, γ-H2AX detection, and apoptosis analysis.

To determine the reasons for the changes in GPR37 expression levels, we conducted extensive research and found that, in the study by Jianhong Chen et al., the gene expression of GPR37 in breast cancer was negatively correlated with DNA methylation levels [50]. In the study by M Toyota et al., GPR37 exhibited high methylation in acute myeloid leukemia [51]. The data imply that the reduced expression of GPR37 in tumors might be associated with DNA methylation. To explore this possibility, we analyzed the methylation status of GPR37 in six different ESCC cell lines. Our analysis revealed that KYSE150 cells, which characteristically display low GPR37 expression, had significantly higher levels of DNA methylation compared to the other cell lines examined. In a study by Pengzhou Kong et al., ZNF750 was identified as a transcription factor that inhibits tumor growth in ESCC [40]. Additionally, there is ample evidence indicating that ZNF750 knockdown promotes proliferation, invasion, migration, and angiogenesis in ESCC [40, 52]. Furthermore, in a study by Ryota Otsuka et al., high expression of ZNF750 was shown to enhance the radiosensitivity of ESCC [53]. Therefore, we suspect that ZNF750 may be a transcription factor for GPR37. After comparing the sequences, we predicted the binding site of ZNF750 at position GPR37-378 and confirmed the accuracy of this site through ChIP and luciferase reporter experiments. These findings indicate that GPR37 is regulated by both DNA methylation and the transcription factor ZNF750.

To investigate the mechanism of action of GPR37 in ESCC, we initially screened the TCGA dataset and found that GPR37 may be negatively correlated with the mTOR signaling pathway. However, current research suggests that GPR37 can promote the mTOR signaling pathway [17, 54]. We examined the expression levels of proteins related to the mTOR signaling pathway and found that, in cells overexpressing GPR37, the protein expression of mTOR decreased, as did the phosphorylation level of AKT. These findings indicate that GPR37 inhibits the activation of the AKT/mTOR signaling pathway in ESCC cells. Next, we treated different ESCC cell lines with activators and inhibitors of AKT and mTOR and evaluated their behavioral characteristics and apoptosis after radiation. These findings confirmed that GPR37 inhibits the development and radioresistance of ESCC through the AKT/mTOR signaling pathway. Using IP-MS, we screened for proteins directly associated with GPR37. Among the top-ranked proteins, ATP1A1 attracted our attention. There is ample evidence suggesting that ATP1A1 may be involved in the activation of AKT and mTOR across various cell type [55–59]. In our research, we observed a decrease in the protein expression of ATP1A1 in cells where GPR37 was overexpressed. Therefore, we can speculate that GPR37 restricts the activation of the AKT/mTOR signaling pathway by facilitating the degradation of ATP1A1. Initially, we confirmed the precision of the Co-IP findings by co-transfecting cells with GPR37-Flag and ATP1A1-HA plasmids, thereby verifying the interaction between the two proteins. Since ATP1A1 is downregulated in cells overexpressing GPR37 but directly interacts with these two proteins, GPR37 is unlikely to regulate transcription and translation to suppress ATP1A1 expression. Therefore, we speculate that GPR37 may promote the degradation of ATP1A1. We treated cells with the proteasome inhibitor MG132, observing a subsequent recovery in the protein level of ATP1A1 in GPR37-overexpressing cells. Furthermore, after transfecting cells with plasmids that overexpress ATP1A1, we noticed an increase in AKT phosphorylation and mTOR protein levels in cells that overexpress GPR37. These findings support the hypothesis that GPR37 restricts the activation of the AKT/mTOR signaling pathway by inducing ATP1A1 degradation in ESCC.

To identify effective ways to increase GPR37 levels in esophageal cancer, we investigated exosomes. Exosomes are small vesicles secreted by cells that encapsulate proteins, nucleic acids, and other substances [25]. Furthermore, research has shown that exosomes can impact cellular functions through the GPR37 signaling pathway [13], and it has also been suggested that exosomes can enhance the radiosensitivity of ESCC [31]. Therefore, we propose a reasonable hypothesis: whether exosomal GPR37 protein levels in ESCC can be increased through exosomes. We extracted exosomes from the conditioned media of wild-type and GPR37-overexpressing ESCC cells and were found that the exosomes carried the GPR37 protein. Moreover, exosomes extracted from the conditioned media of cells with high GPR37 levels exhibited even greater GPR37 protein levels. In our experiments, after isolating exosomes, we reintroduced them into the culture medium of ESCC cells. Subsequently, we observed that the cells internalized the exosomes, which in turn led to alterations in the levels of the GPR37 protein. This also led to alterations in the tumor phenotype and radiosensitivity of the recipient cells. This discovery suggested that it may be possible to use exosomes to target GPR37 for the clinical treatment of ESCC. These findings provide a rationale for the suboptimal radiotherapy response in ESCC patients with low GPR37 expression, suggesting potential strategies to enhance radiotherapy sensitivity. In the future studies, we aim to amass a larger collection of post-radiotherapy human ESCC tissue samples, fabricate organoids, and generate genetically modified mouse models. This will provide a robust foundation for translating our laboratory findings into clinical research. Moreover, we propose to explore the encapsulation and modification of exosomes to enrich GPR37 protein content, target ESCC cells specifically, deliver chemotherapeutic agents, and mitigate side effects. Post-surgical immunohistochemical assessment of GPR37 expression in resected cancer tissue will enable the stratification of ESCC patients. Those with low GPR37 expression will be allocated to a group where we will investigate the efficacy of combining exosomes with radiotherapy, thereby seeking an innovative approach to augment therapeutic outcomes.

## Conclusions

This study demonstrated that GPR37 inhibits the proliferation, metastasis, and radioresistance of ESCC cells. The expression level of GPR37 is regulated by DNA methylation and ZNF750. It may exert its effects by inhibiting the activation of the AKT/mTOR signaling pathway by promoting ubiquitination of ATP1A1, and it can also mediate intercellular transfer through Exo-GPR37. In summary, GPR37 level could act as a biological indicator for the proliferation and metastatic behavior of ESCC, as well as a pivotal gene contributing to radioresistance. It is a promising candidate for a significant molecular target in the clinical management of ESCC.

## Supplementary information

supplementary file

WB raw data

## Data Availability

The data generated or analyzed during this study are accessible upon reasonable request from the corresponding author.
