# Supplementary material for: GPR37-enhanced ubiquitination of ATP1A1 inhibits tumor progression and radiation resistance in esophageal squamous cell carcinoma
Source: Cell Death Dis. 2024 Dec 27;15(12):933. doi: 10.1038/s41419-024-07240-1 (PMC11681203; doi:10.1038/s41419-024-07240-1)
Supplement: Supplementary file 1 — supplementary file [file 41419_2024_7240_MOESM1_ESM.docx]

**Supplementary table and figure legends**

**Supplementary Figure 1: Correlation between GPR37 mRNA levels and clinical stage in ESCC.**

(A-C) Low GPR37 mRNA level was associated with advanced T stage(A), N stage(B), and pathologic stage(C) in TCGA ESCC. **p<0.01.

**Supplementary Figure 2: The methylation degree of CpG locus in the promoter region of GPR37 was negatively correlated with the mRNA expression of GPR37 in ESCA.**

(A) The average methylation degree of GPR37 promoter region was negatively correlated with GPR37 mRNA expression. (B-P) The degree of methylation of the CpG locus in the promoter region of GPR37 correlates with the mRNA level of GPR37, and the CpG locus are cg17052813(B)、cg26141626(C)、cg07392724(D)、cg16847696(E)、cg17152484(F)、cg14311320(G)、cg01667837(H)、cg23428445(I)、cg09458673(J)、cg22230167(K)、cg02960853(L)、cg27533119(M)、cg23799901(N)、cg07376282(O) and cg26278103(P), respectively.

**Supplementary Figure 3: ZNF750 regulates GPR37 expression in KYSE510 cells.**

(A) Western blotting was used to detect the ZNF750 protein level in KYSE510 cells. (B) The mRNA levels of ZNF750 in KYSE510-overexpressing cells were detected via qRT‒PCR (n=3). (C) The expression level of GPR37 in KYSE510 cells overexpressing ZNF750 was measured by qRT‒PCR (n=3). (D) Western blotting was used to detect the GPR37 protein level in KYSE510 cells. ***p<0.001, ****p<0.0001.

**Supplementary Figure 4: Effect of GPR37 overexpression on radioresistance in KYSE510 cells.**

(A-C) GSEA analysis showed the correlation between GPR37 expression level and “DNA Repair” (A), “Reactive oxygen species pathway” (B), and “UV response UP” (C) pathway. (D) Western blotting was used to measure the protein expression level of GPR37 in KYSE510 cells. (E) qRT‒PCR was used to measure the mRNA expression level of GPR37 in KYSE510 cells (n=3). (F) Colonies of KYSE510 cells (after 14 days of culture) after exposure to different doses of X-rays (n=3). The 0 Gy group is used as the control group and a single-target multitarget model was used to fit the cell survival curve. (G) After 8 Gy of X-ray treatment, immunofluorescence was used to detect the levels of γ-H2AX in KYSE510 cells at 0.5 h and 4 h, and the 0 Gy group is used as the control group (n=3). Scale bar: 10 µm. (H) Flow cytometry was used to detect apoptosis in KYSE510 cells after 8 Gy irradiation (n=3). *p<0.05, **p<0.01, ***p<0.001.

**Supplementary Figure 5: Changes in the proliferation, migration and invasion of GPR37-overexpressing KYSE510 cells.**

(A-D) GSEA analysis in TCGA ESCC showed the correlation between GPR37 expression level and “G2M checkpoint” (A), “Glycolysis” (B), “Epithelial mesenchymal transition” (C) and “Angiogenesis” (D) pathway. (E) The proliferation of GPR37-overexpressing KYSE510 cells was determined via the CCK8 method (n=3). (F and G) A wound healing assay (F) and Matrigel-free transwell assay (G) were used to detect the migration of KYSE510 cells overexpressing GPR37 (n=3). Scale bar: 100 µm. (H) The invasion of KYSE510 cells overexpressing GPR37 was determined via the Matrigel-containing transwell method (n=3). Scale bar: 100 µm. (I) Western blotting was used to detect the protein level of EMT pathway. **p<0.01, ****p<0.0001.

**Supplementary figure 6: SC79 and MHY1485 treatments promote the proliferation, migration, invasion, and radiation tolerance of KYSE510 GPR37 cells.**

(A) Western blotting was used to measure the levels of AKT/mTOR-related proteins in KYSE510 cells. (B) Western blotting was used to detect changes in the KYSE510 cell protein after SC79 and MHY1485 treatment. (C) The CCK-8 method was used to determine the proliferation of KYSE510 cells after drug treatment (n=3). (D) Cell migration after drug treatment was measured in KYSE510 cells using a wound healing assay(n=3). The wound width at 0h was normalized. Scale bar: 100 μm. (E) Cell migration after drug treatment was measured in KYSE510 cells using a transwell assay without Matrigel (n=3). Scale bar: 100 μm. (F) Transwell assays with Matrigel were used to measure the invasion of KYSE510 cells after drug treatment (n=3). Scale bar: 100 μm. (G and H) Flow cytometry was used to evaluate the apoptosis of SC79 (G)- and MHY1485 (H)- treated KYSE510 cells after 8 Gy irradiation (n=3). *p<0.05, **p<0.01, ***p<0.001, ****p<0.0001.

**Supplementary figure 7: MK2206 and TORIN1 treatment inhibits the proliferation, migration, invasion, and radiation tolerance of KYSE450 shGPR37 cells.**

(A) Western blotting was used to detect changes in the protein expression of shGPR37 KYSE450 cells treated with MK2206 and TORIN1. (B) The proliferation of KYSE450 cells after drug treatment was determined by the CCK8 method (n=3). (C and D) Cell migration was measured in KYSE450 cells after drug treatment using a wound healing assay (C) and a transwell assay (D) without Matrigel (n=3). Scale bar: 100 μm. (E) Transwell assays with Matrigel were used to measure the invasion of KYSE450 cells after drug treatment (n=3). Scale bar: 100 μm. (F) Flow cytometry was used to evaluate the apoptosis of SC79- and MHY1485-treated KYSE450 cells after 8 Gy irradiation (n=3). *p<0.05, **p<0.01, ***p<0.001, ****p<0.0001.

**Supplementary figure 8: In KYSE510 cells, GPR37 promotes the degradation of ATP1A1 and inhibits the phosphorylation of AKT.**

(A) GPR37 co-IP samples from KYSE510 cells were analyzed via Western blotting. (B) Immunofluorescence detection of the location of GPR37 and ATP1A1 in KYSE510 cells. (C) Western blotting and qRT-PCR were used to detect the expression level of ATP1A1 in KYSE510 cells (n=3). (D) Western blotting was used to detect ATP1A1 protein levels in KYSE510 cells after MG132 treatment. (E) Western blotting was used to detect the protein levels in KYSE510 cells overexpressing ATP1A1.

**Supplementary figure 9: Effect of Exo-GPR37 on the function and radiosensitivity of KYSE510 cells.**

(A) Nanoparticle tracking analysis was used to determine the size of the exosomes. (B) TEM image showing the morphology of the exosomes. Scale bar, 200 nm. (C) Western blotting showed that GPR37 and the exosomal protein markers CD63 and TSG101 were enriched in E-cell exosomes secreted by KYSE510 cells. (D) An exosome uptake assay showed that PKH26-labeled Exos were taken up by KYSE510 cells. Scale bar, 100 μm. (E) Western blotting (left) and qRT-PCR (right) were used to detect the GPR37 expression level in KYSE510 cells after coincubation with Exo-vector or Exo-GPR37 (n=3). (F) The proliferation of KYSE510 cells coincubated with Exo-vector or Exo-GPR37 was determined via the CCK8 method (n=3). (G and H) Wound healing (G) and transwell (H) assays were used to detect the migration and invasion of KYSE510 cells after coincubation with Exo-vector and Exo-GPR37 (n=3). Scale bar: 100 µm. (I) Flow cytometry was used to assess the radiosensitivity of KYSE510 cells after coincubation with Exo-vector and Exo-GPR37 (n=3). (J) Western blotting was used to detect the expression level of AKT/mTOR-related proteins in KYSE510 cells after coincubation with Exo-vector or Exo-GPR37. (K) An exosome uptake assay showed that PKH26-labeled Exos were taken up by HEEC cells. Scale bar, 100 μm. (L) Flow cytometry was used to assess the radiosensitivity of HEEC cells after coincubation with Exo-vector and Exo-GPR37 (n=3). ns: not significant, *p<0.05, **p<0.01, ***p<0.001.

**Supplementary table 1:** **Radioresistance verification of KYSE410R**

**Supplementary table 2: Correlation between GPR37 expression and clinical case parameters in 97 ESCC patients**

**Supplementary table 3: Upregulation of GPR37 increases the radiosensitivity of ESCC cells**

**Supplementary table 4: Knockdown of GPR37 reduces the radiosensitivity of KYSE450 cells**

**Supplementary table 5: Top 10 of the MS results of the IP samples of GPR37**

**Supplementary figure 1**


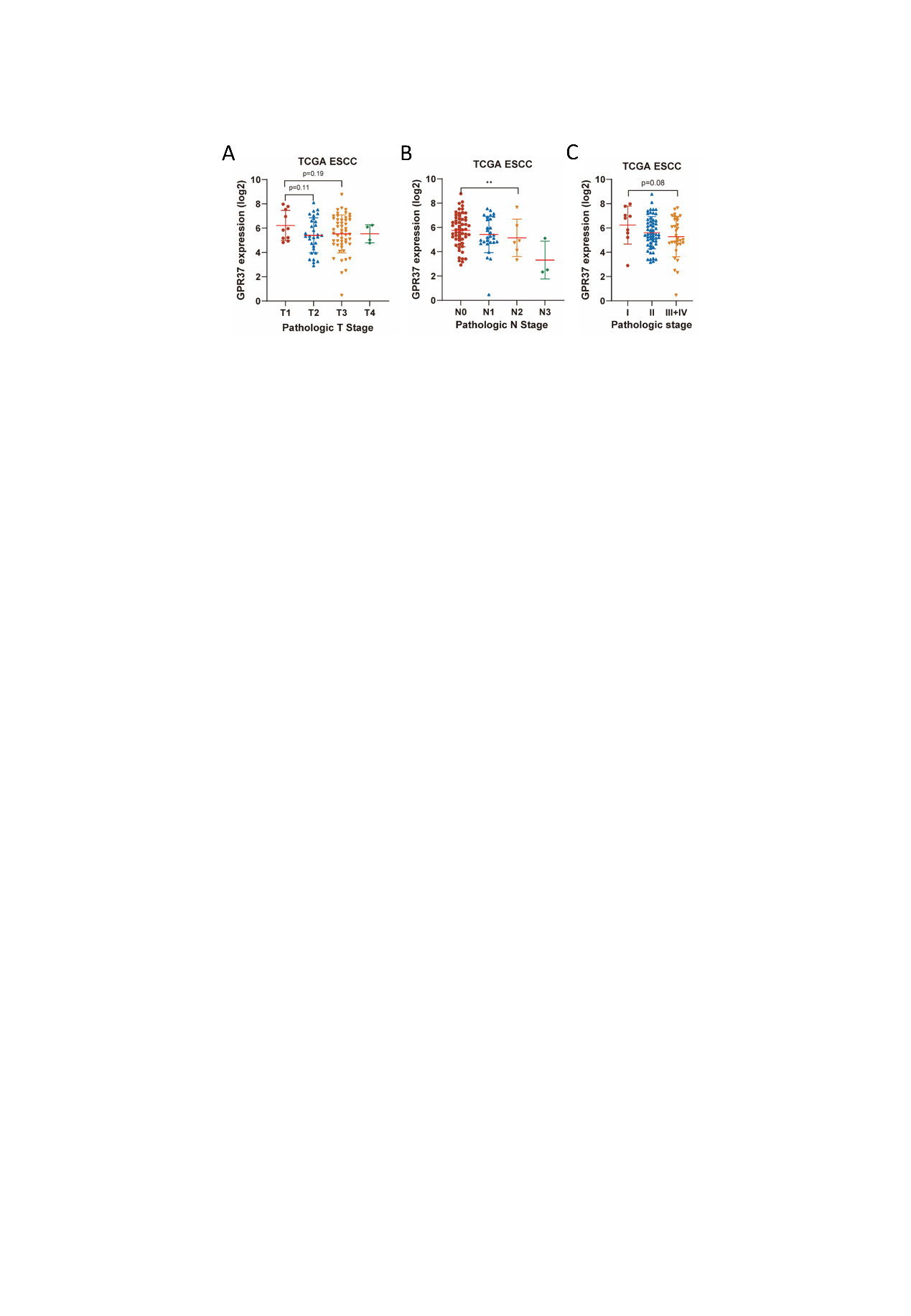


**Supplementary figure 2**


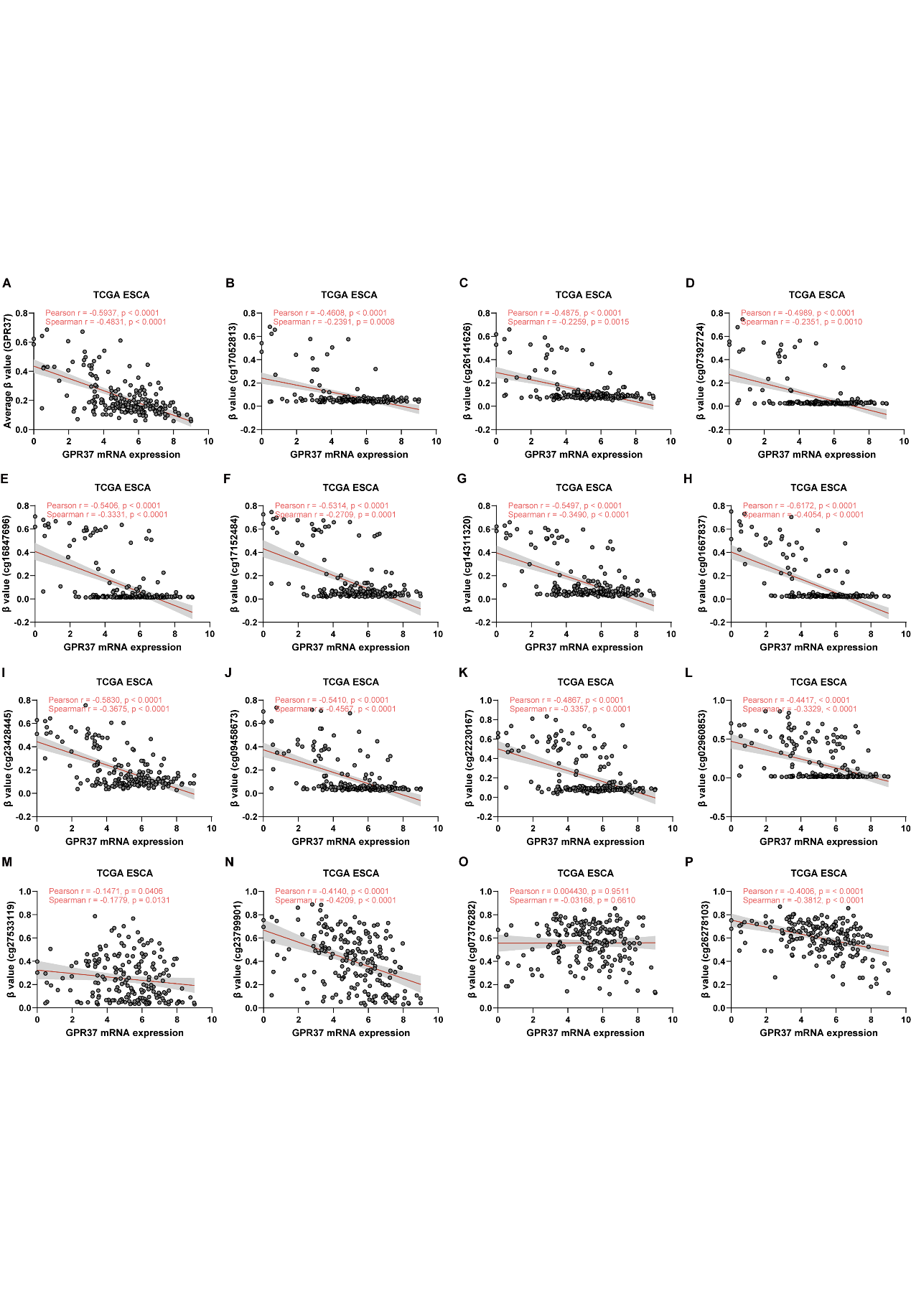


**Supplementary figure 3**


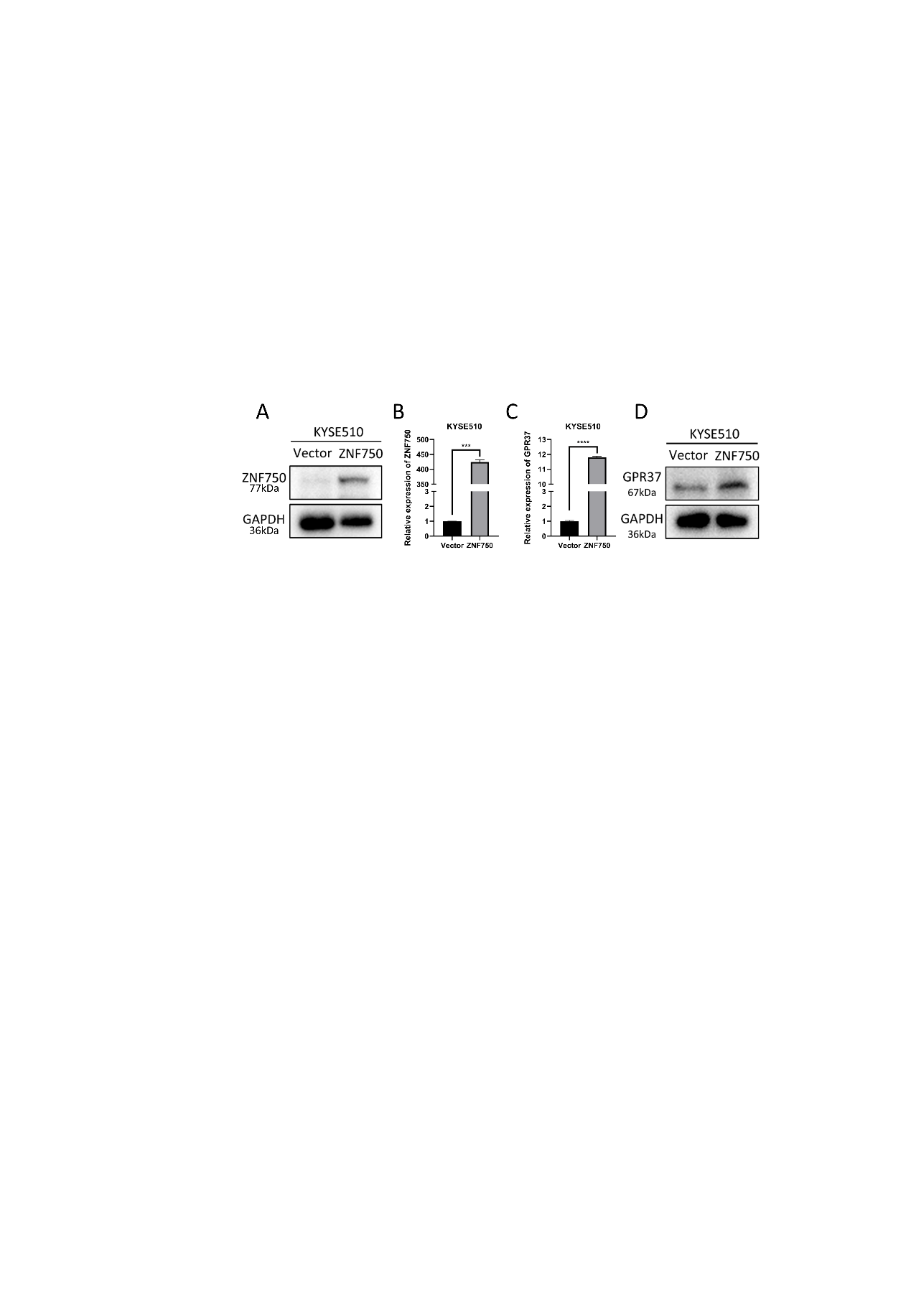


**Supplementary figure 4**


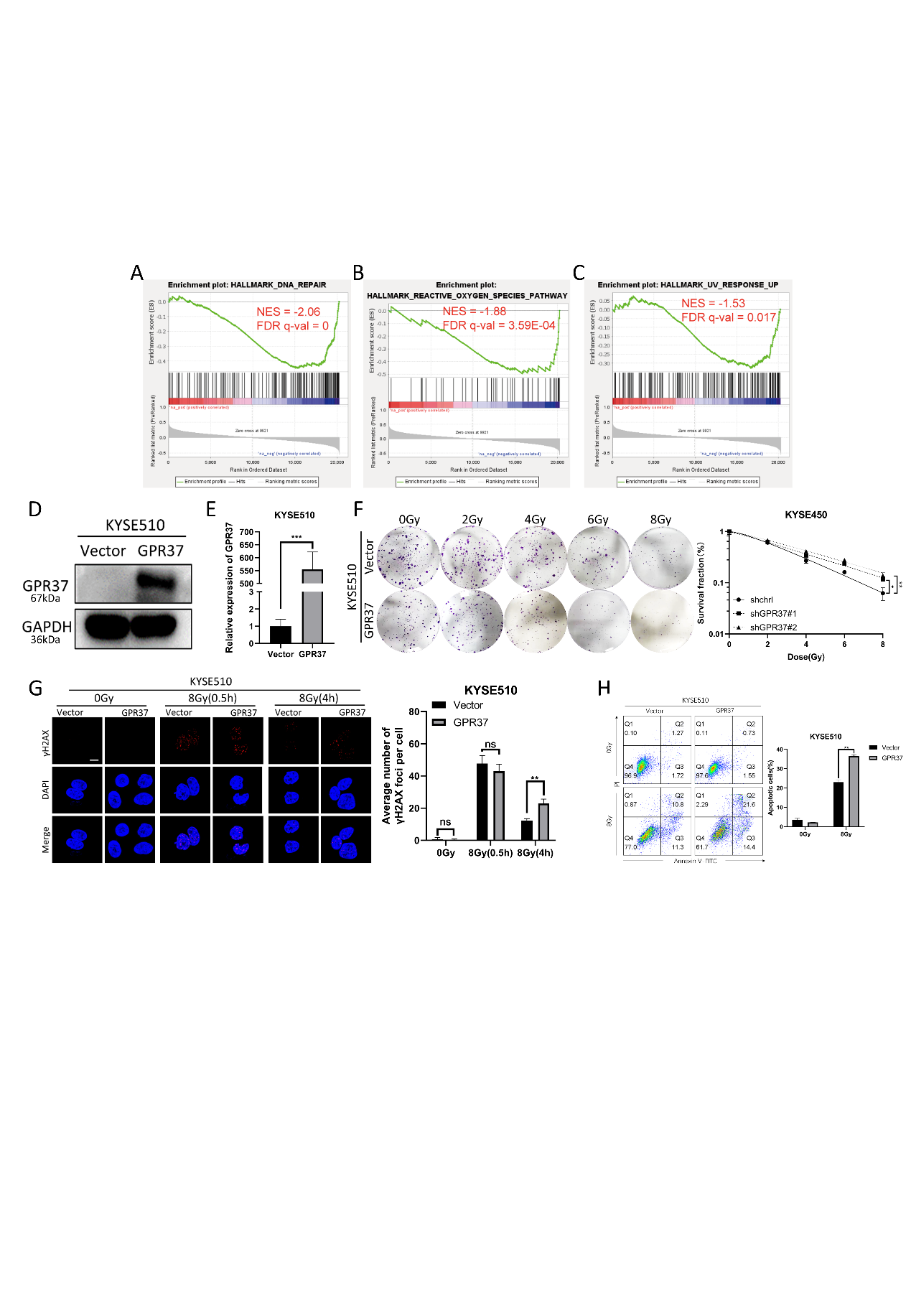


**Supplementary figure 5**


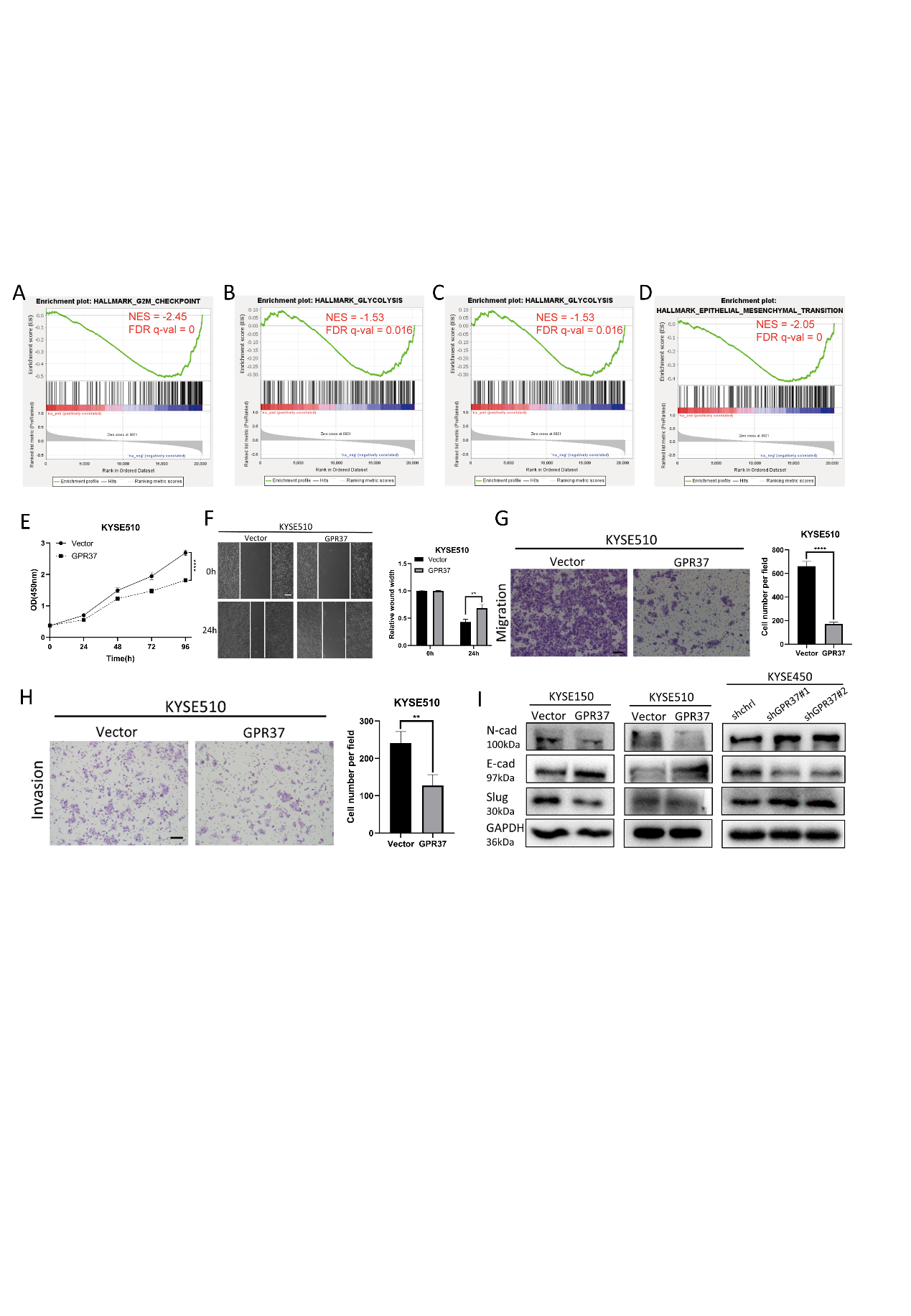


**Supplementary figure 6**


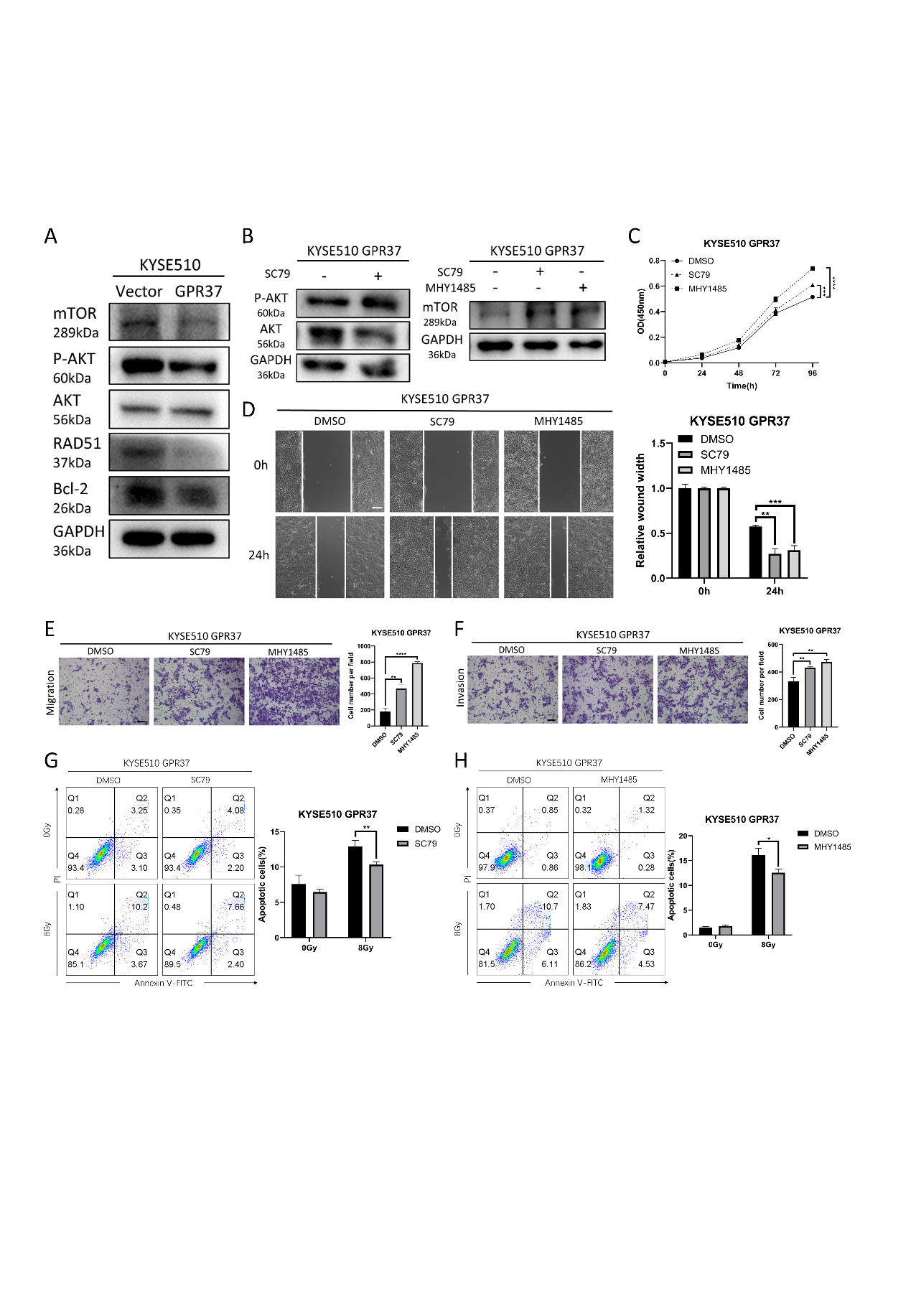


**Supplementary figure 7**


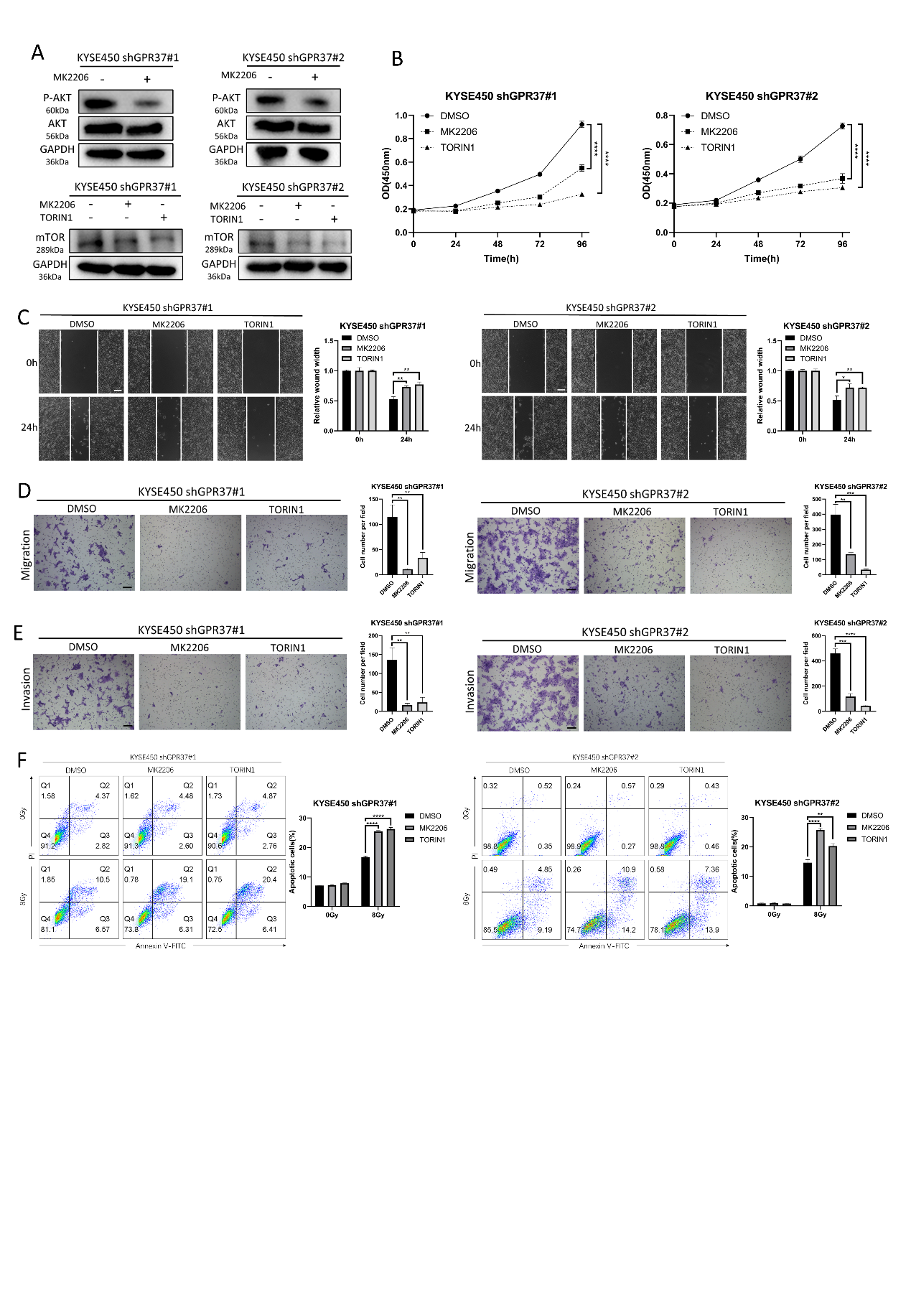


**Supplementary figure 8**


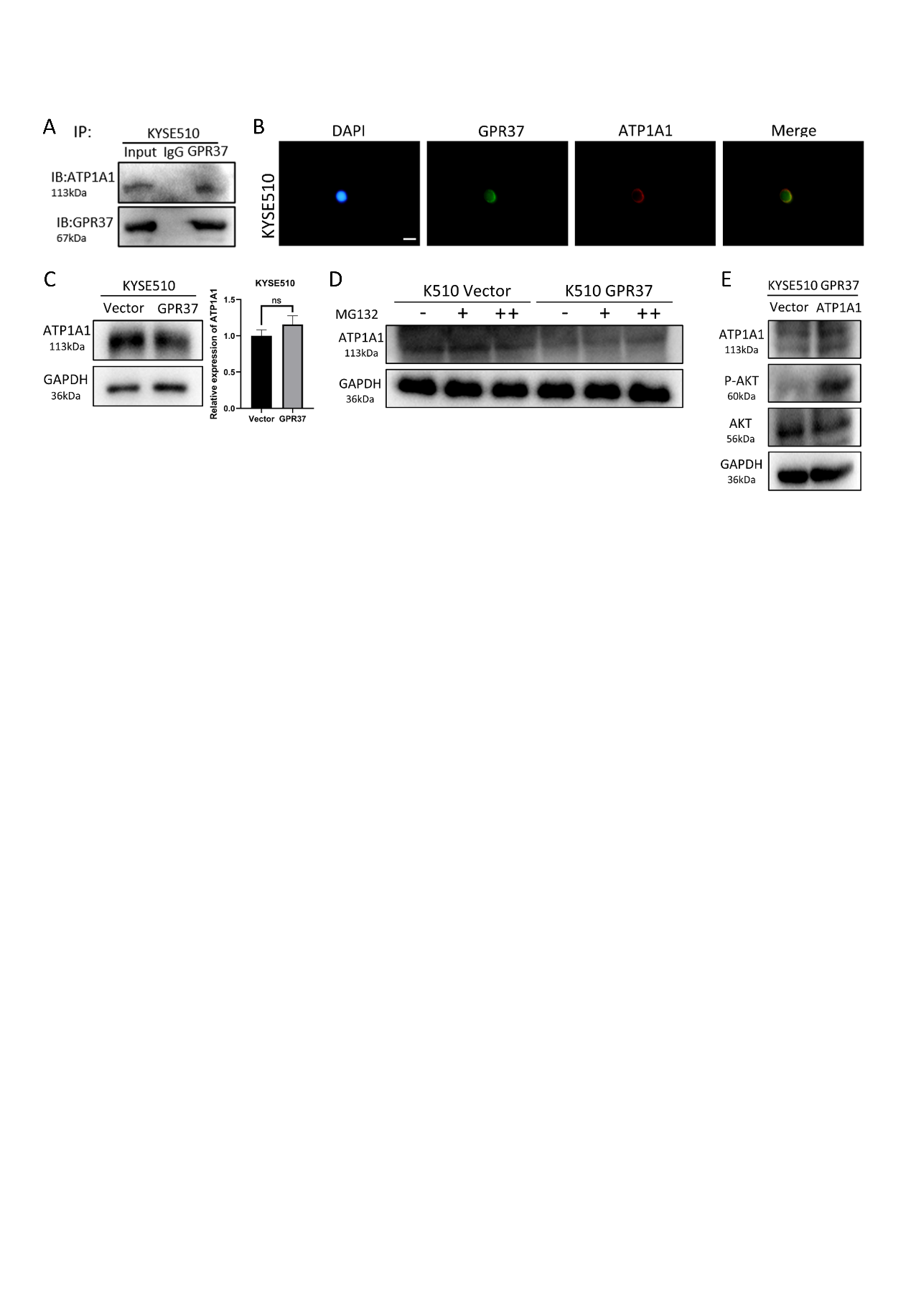


**Supplementary figure 9**


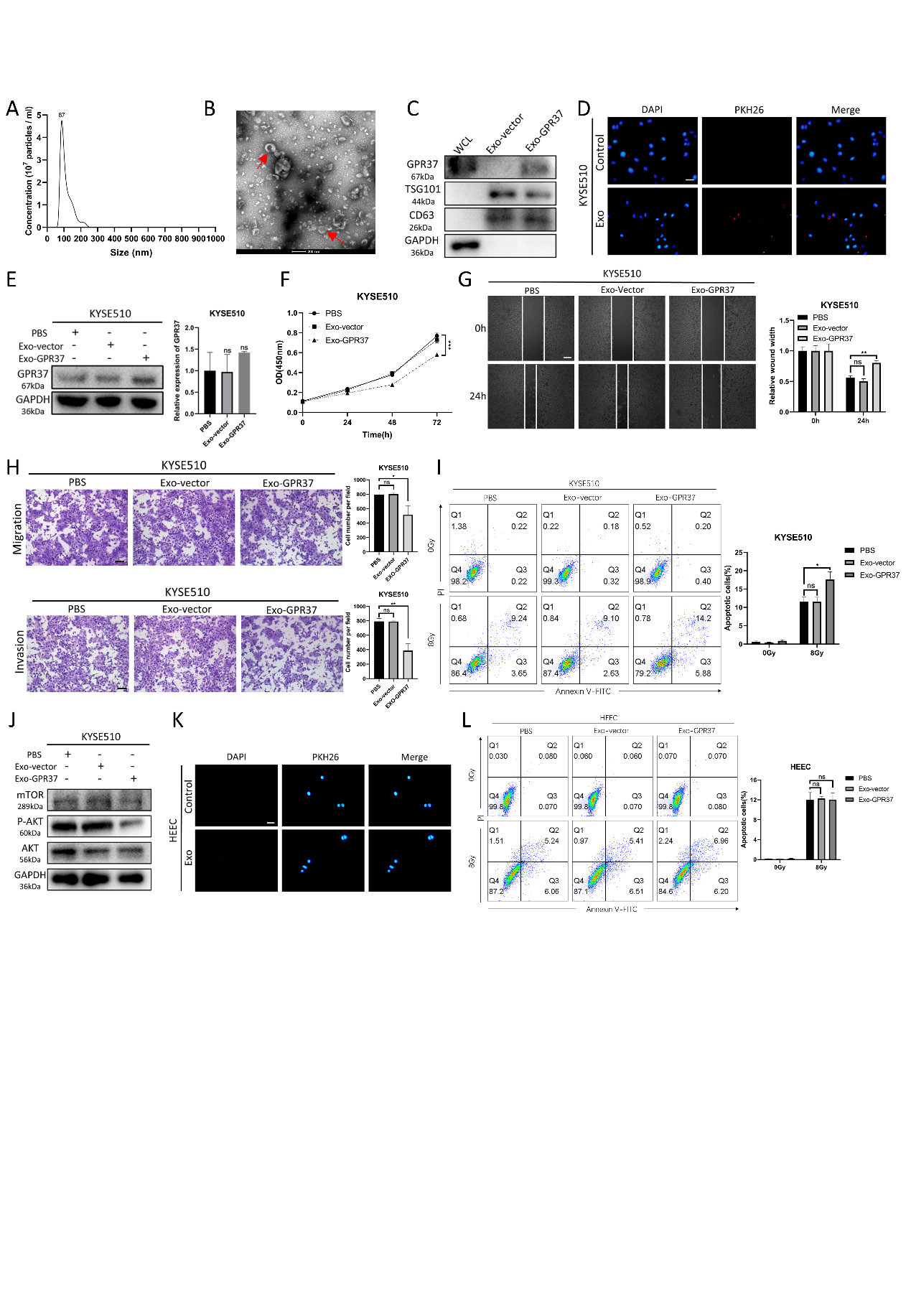


**Supplementary Table. 1 Radioresistance verification of KYSE410R**

| **Group** | **D_0_** | **Dq** | **SF_2_** | **SER** |
| --- | --- | --- | --- | --- |
| **KYSE410** | **3.792** | **0.822** | **0.408** |  |
| **KYSE410R** | **4.249** | **3.602** | **0.951** | **0.892** |

**Supplementary Table. 2 Correlation between GPR37 expression and clinical case parameters in 97 ESCC patients**

| **Characteristics** | **GPR37-Low** | | **GPR37-High** | **P value** | | |
| --- | --- | --- | --- | --- | --- | --- |
| **n** | **47** | | **50** |  | | |
| **Stage, n (%)** |  | |  | **0.023** | | |
| Stage I | 0 (0%) | | 4 (4.1%) |  | | |
| Stage II | 27 (27.8%) | | 35 (36.1%) |  | | |
| Stage III | 20 (20.6%) | | 11 (11.3%) |  | | |
| **Age, n (%)** |  |  | | | 0.380 |  |
| ≥65 | 25 (25.8%) | 31 (32%) | | |  |  |
| ＜65 | 22 (22.7%) | 19 (19.6%) | | |  |  |
| **Tumor size, n (%)** |  |  | | | 0.516 |  |
| ≥10 | 29 (29.9%) | 34 (35.1%) | | |  |  |
| ＜10 | 18 (18.6%) | 16 (16.5%) | | |  |  |
| **Sex, n (%)** |  | |  | 0.681 | | |
| Male | 39 (40.2%) | | 43 (44.3%) |  | | |
| Female | 8 (8.2%) | | 7 (7.2%) |  | | |
| **lymph node metastasis, n (%)** |  | |  | **0.034** | | |
| + | 28 (28.9%) | | 19 (19.6%) |  | | |
| - | 19 (19.6%) | | 31 (32%) |  | | |
| **vascular or nerve invasion, n (%)** |  | |  | **0.020** | | |
| + | 16 (16.5%) | | 7 (7.2%) |  | | |
| - | 31 (32%) | | 43 (44.3%) |  | | |

**Supplementary Table. 3 Upregulation of GPR37 increases the radiosensitivity of ESCC cells**

| **Group** | | **D_0_** | **Dq** | **SF_2_** | **SER** |
| --- | --- | --- | --- | --- | --- |
| **KYSE150** | **Vector** | **3.303** | **1.123** | **0.514** |  |
|  | **GPR37** | **1.781** | **0.741** | **0.398** | **1.855** |
| **KYSE510** | **Vector** | **3.612** | **1.076** | **0.513** |  |
|  | **GPR37** | **2.856** | **0.885** | **0.439** | **1.265** |

**Supplementary Table. 4** **Knockdown of GPR37 reduces the radiosensitivity of KYSE450 cells**

| **Group** | | **D_0_** | **Dq** | **SF_2_** | **SER** |
| --- | --- | --- | --- | --- | --- |
| **KYSE450** | **shchrl** | **2.526** | **1.406** | **0.608** |  |
|  | **shGPR37#1** | **3.916** | **1.427** | **0.622** | **0.645** |
|  | **shGPR37#2** | **3.849** | **1.692** | **0.679** | **0.656** |

**Supplementary Table. 5** **Top 10 of the MS results of the IP samples of GPR37**

| **Peptides** | **Gene Symbol** |
| --- | --- |
| **14** | **GPR37** |
| **13** | **TUBB4B** |
| **9** | **BCLAF1** |
| **5** | **ATP1A1** |
| **5** | **HSP90AB1** |
| **5** | **Ywhaz** |
| **4** | **CKAP4** |
| **4** | **SFXN1** |
| **4** | **KRT73** |
| **3** | **EEF1G** |
| **3** | **HADHA** |
