# Supplementary material for: GPR37-enhanced ubiquitination of ATP1A1 inhibits tumor progression and radiation resistance in esophageal squamous cell carcinoma
Source: Cell Death Dis. 2024 Dec 27;15(12):933. doi: 10.1038/s41419-024-07240-1 (PMC11681203; doi:10.1038/s41419-024-07240-1)
Supplement: Supplementary file 2 — WB raw data [file 41419_2024_7240_MOESM2_ESM.docx]

GPR37:







| lane | 1 | 2 | 3 | 4 | 5 |
| --- | --- | --- | --- | --- | --- |
| sample | marker | KYSE410 | KYSE410R | TE-1 | TE-1R |

GAPDH:





| lane | 1 | 2 | 3 | 4 | 5 | 6 | 7-15 |
| --- | --- | --- | --- | --- | --- | --- | --- |
| sample | marker | KYSE410 | KYSE410R | TE-1 | TE-1R | marker | others |

Corresponds to Fig 1.B in the article:


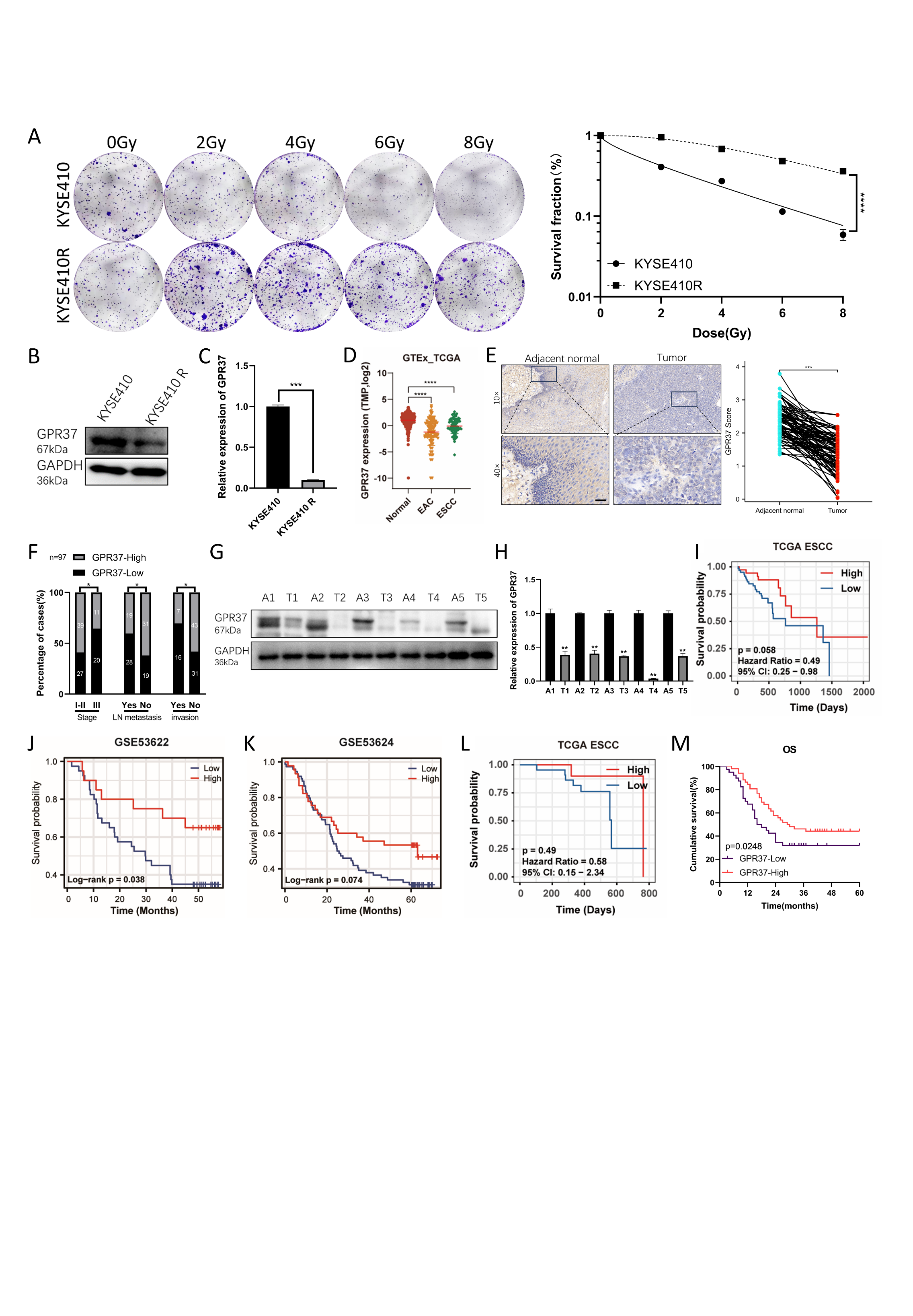


GPR37:


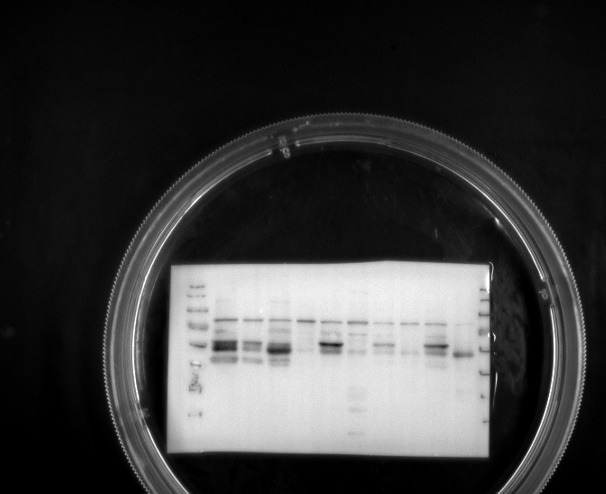




| lane | 1 | 2 | 3 | 4 | 5 | 6 | 7 | 8 | 9 | 10 | 11 | 12 |
| --- | --- | --- | --- | --- | --- | --- | --- | --- | --- | --- | --- | --- |
| sample | marker | A1 | T1 | A2 | T2 | A3 | T3 | A4 | T4 | A5 | T5 | marker |

GAPDH:


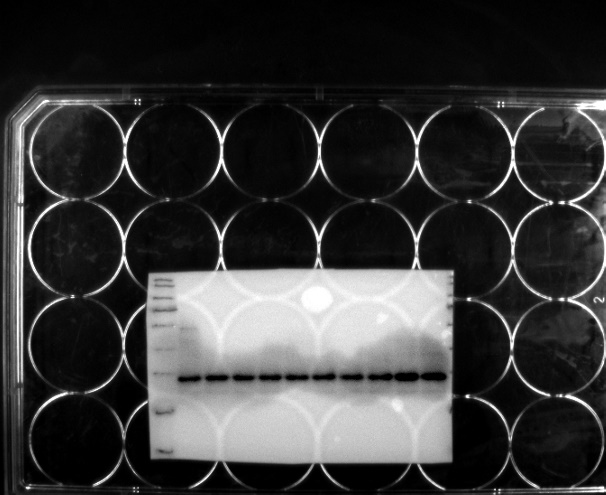




| lane | 1 | 2 | 3 | 4 | 5 | 6 | 7 | 8 | 9 | 10 | 11 | 12 |
| --- | --- | --- | --- | --- | --- | --- | --- | --- | --- | --- | --- | --- |
| sample | marker | A1 | T1 | A2 | T2 | A3 | T3 | A4 | T4 | A5 | T5 | marker |

Corresponds to Fig 1.G in the article:


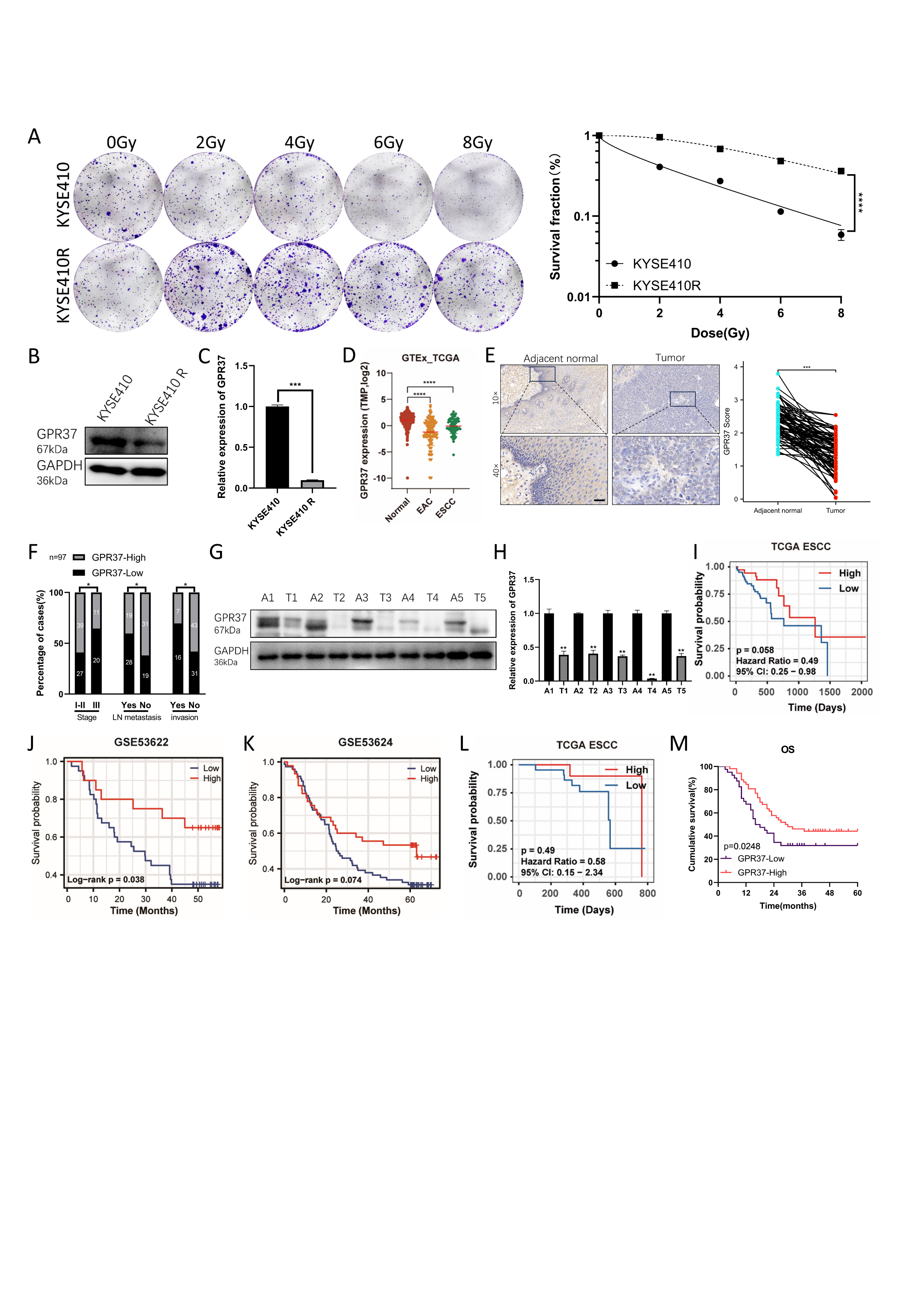


GPR37:


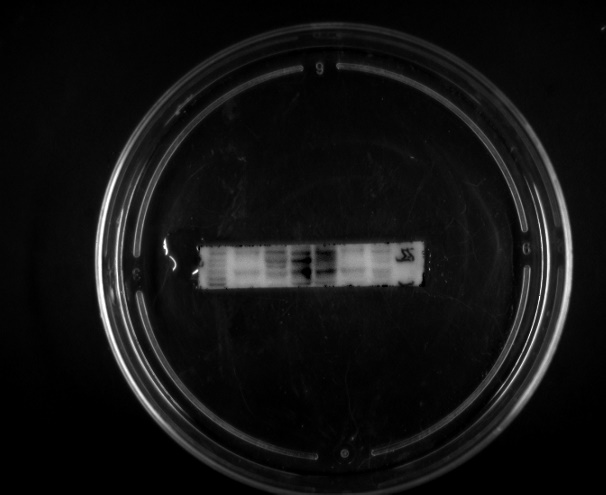




| lane | 1 | 2 | 3 | 4 | 5 | 6 | 7 | 8 |
| --- | --- | --- | --- | --- | --- | --- | --- | --- |
| sample | HEEC | KYSE30 | KYSE150 | KYSE410 | KYSE450 | KYSE510 | TE-1 | marker |

GAPDH:


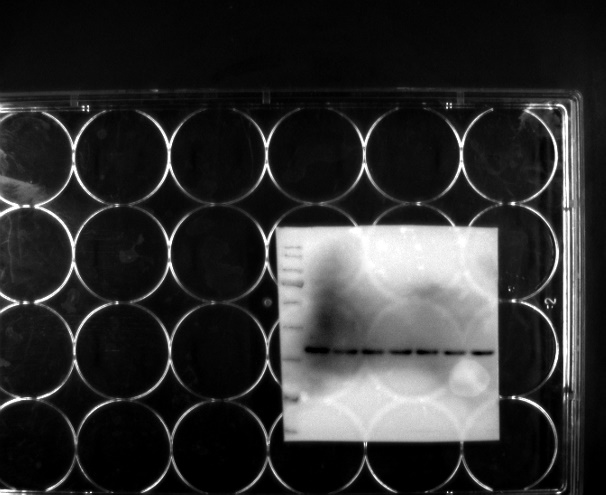




| lane | 1 | 2 | 3 | 4 | 5 | 6 | 7 | 8 |
| --- | --- | --- | --- | --- | --- | --- | --- | --- |
| sample | marker | HEEC | KYSE30 | KYSE150 | KYSE410 | KYSE450 | KYSE510 | TE-1 |

Corresponds to Fig 2.C in the article:


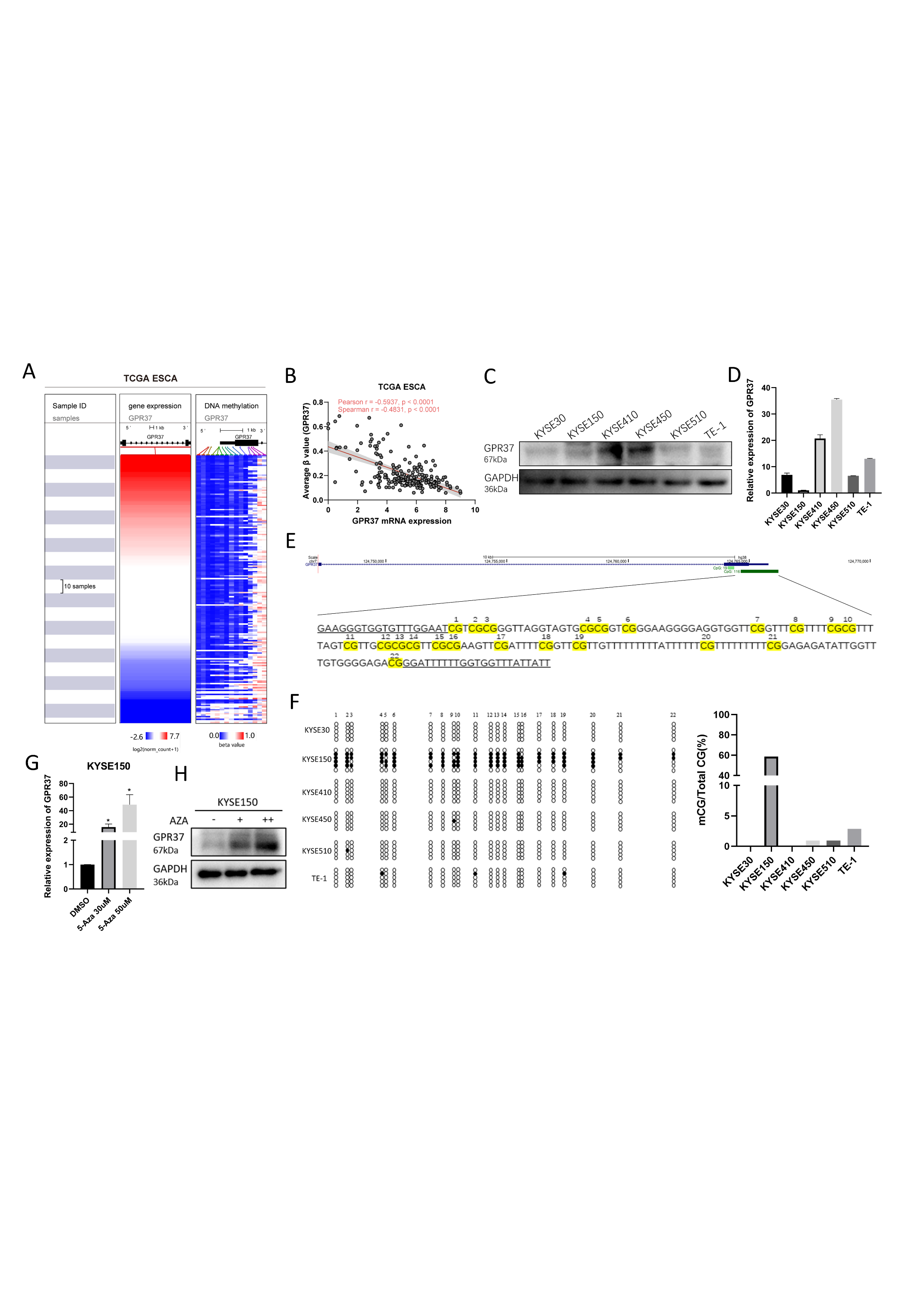


GPR37:


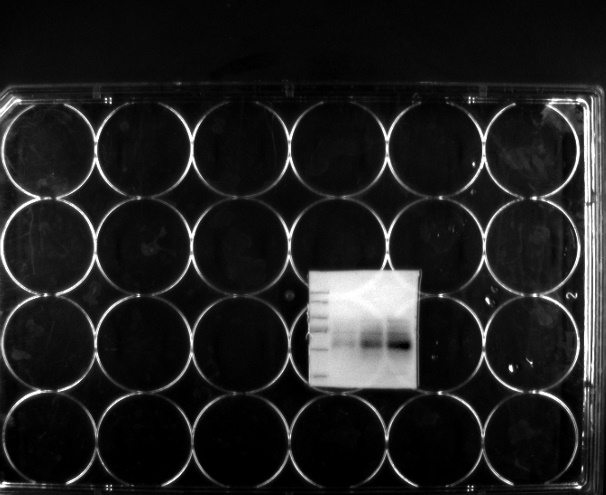




| lane | 1 | 2 | 3 | 4 |
| --- | --- | --- | --- | --- |
| sample | marker | KYSE150+PBS | KYSE150+AZA 30μM | KYSE150+AZA 50μM |

GAPDH:


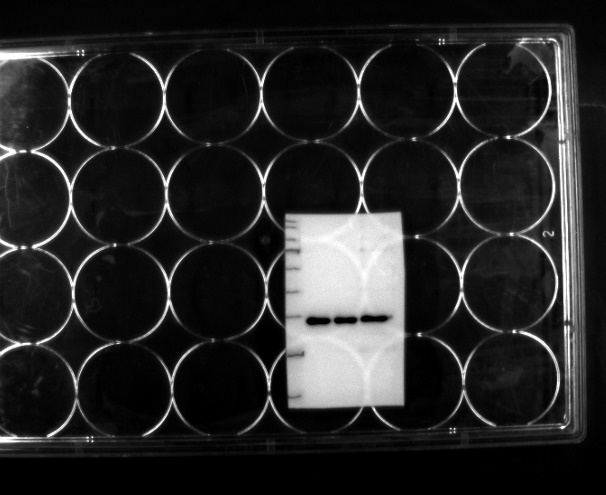




| lane | 1 | 2 | 3 | 4 |
| --- | --- | --- | --- | --- |
| sample | marker | KYSE150+PBS | KYSE150+AZA 30μM | KYSE150+AZA 50μM |

Corresponds to Fig 2.H in the article:


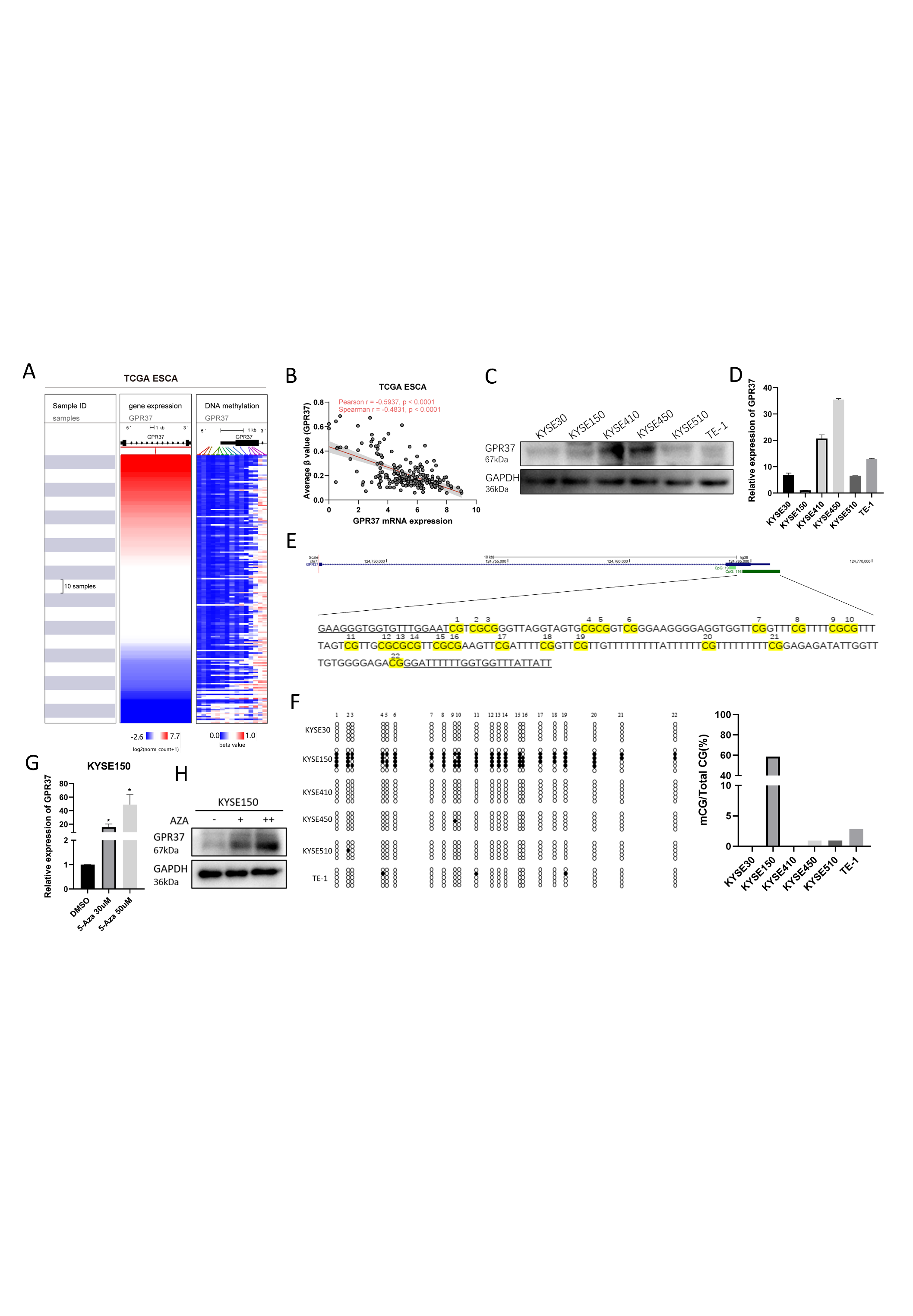


ZNF750:


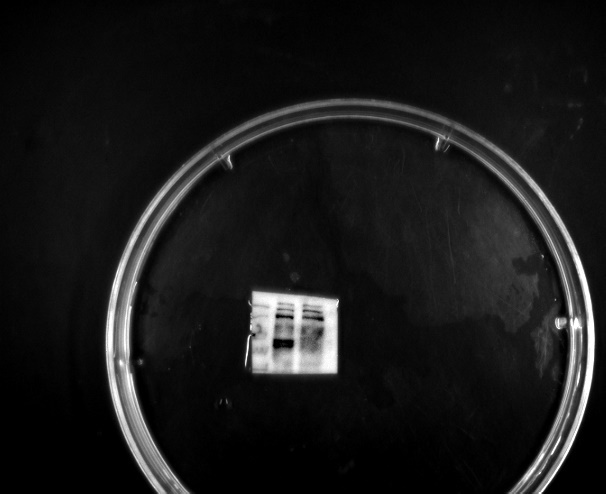




| lane | 1 | 2 | 3 |
| --- | --- | --- | --- |
| sample | marker | KYSE410 | KYSE410R |

GAPDH:


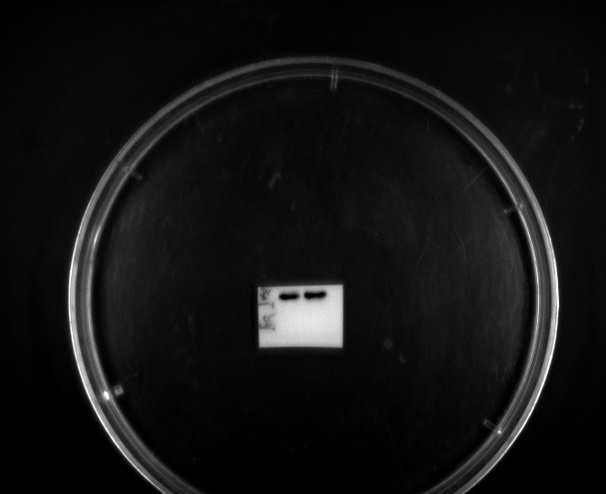




| lane | 1 | 2 | 3 |
| --- | --- | --- | --- |
| sample | marker | KYSE410 | KYSE410R |

Corresponds to Fig 3.B in the article:


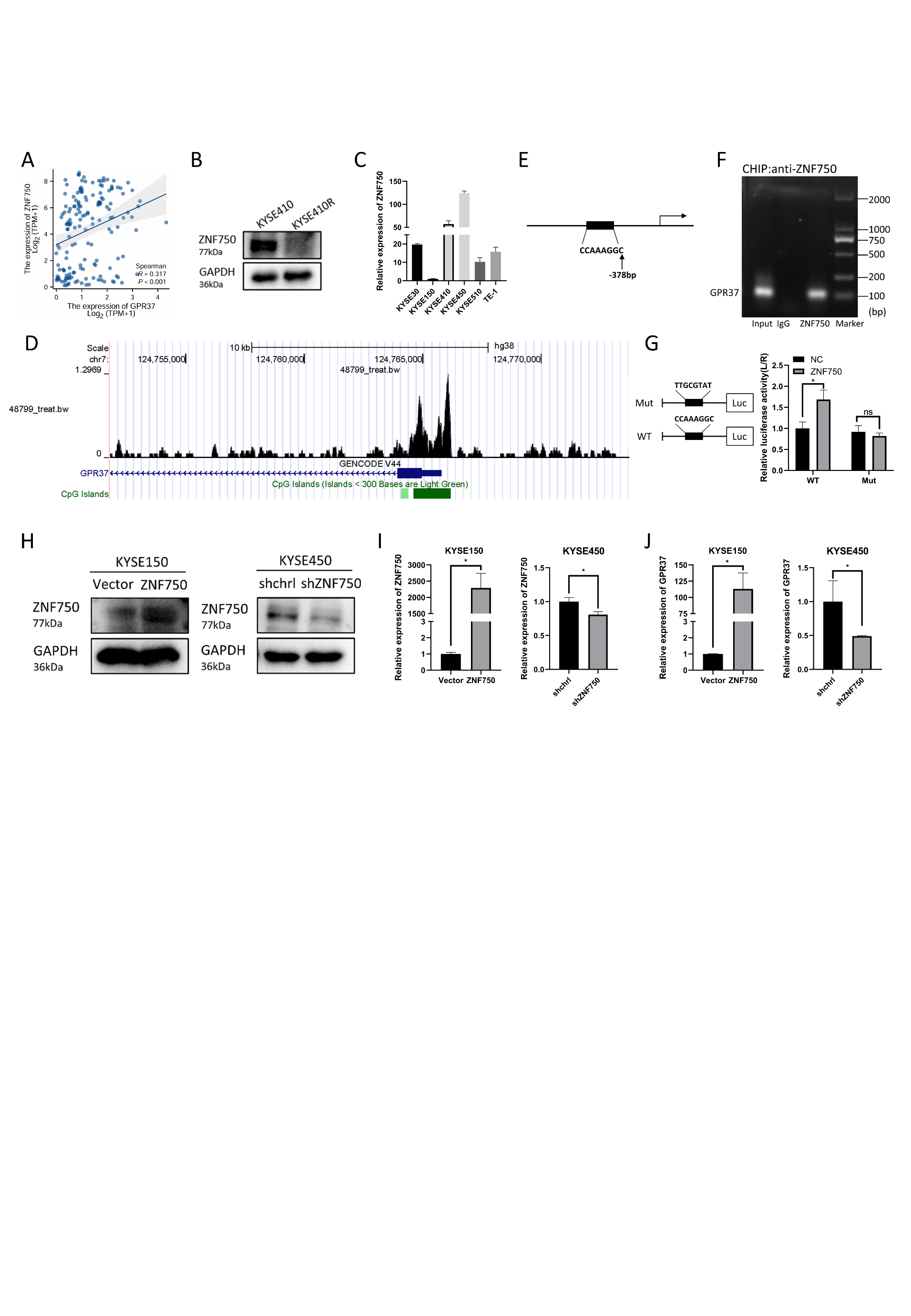


ZNF750:


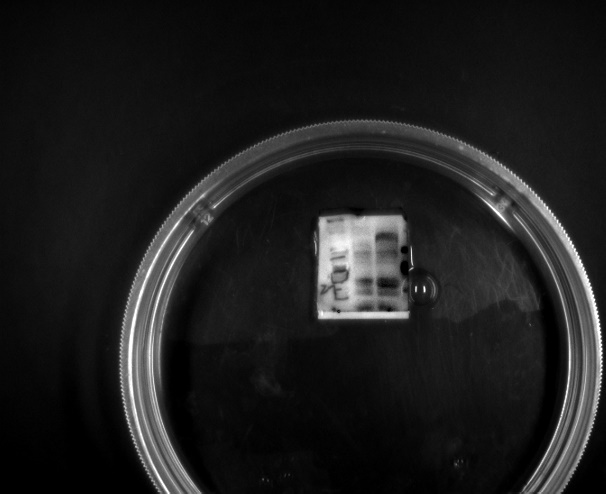




| lane | 1 | 2 | 3 |
| --- | --- | --- | --- |
| sample | marker | KYSE150  Vector | KYSE150  ZNF750 |

GAPDH:


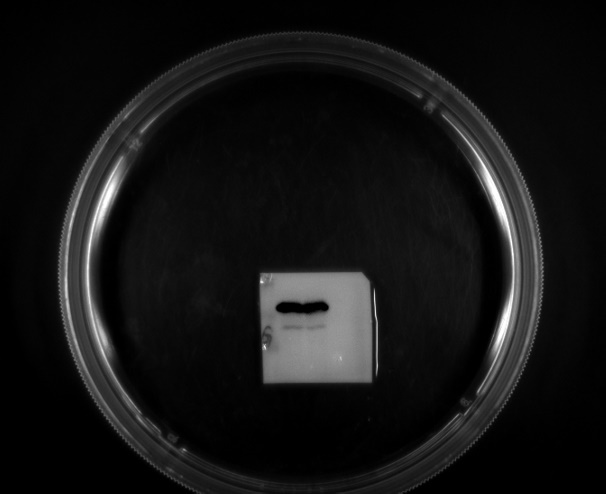




| lane | 1 | 2 | 3 |
| --- | --- | --- | --- |
| sample | marker | KYSE150  Vector | KYSE150  ZNF750 |

ZNF750:


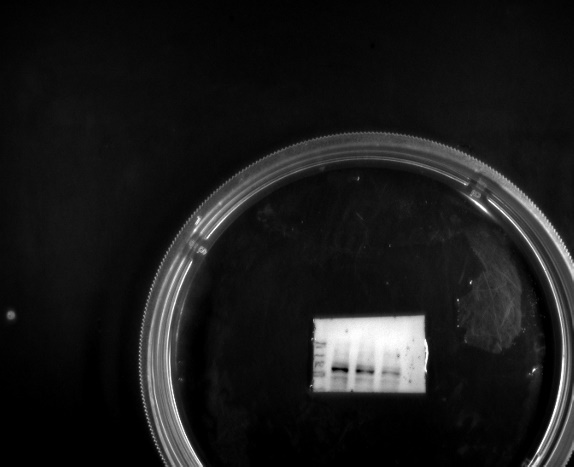




| lane | 1 | 2 | 3 | 4 |
| --- | --- | --- | --- | --- |
| sample | marker | KYSE450 | KYSE450  shchrl | KYSE450  ShZNF750 |

GAPDH:


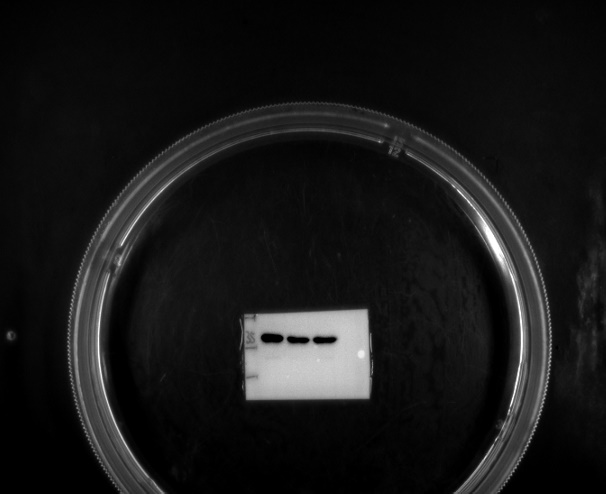




| lane | 1 | 2 | 3 | 4 |
| --- | --- | --- | --- | --- |
| sample | marker | KYSE450 | KYSE450shchrl | KYSE450 shZNF750 |

Corresponds to Fig 3.H in the article:


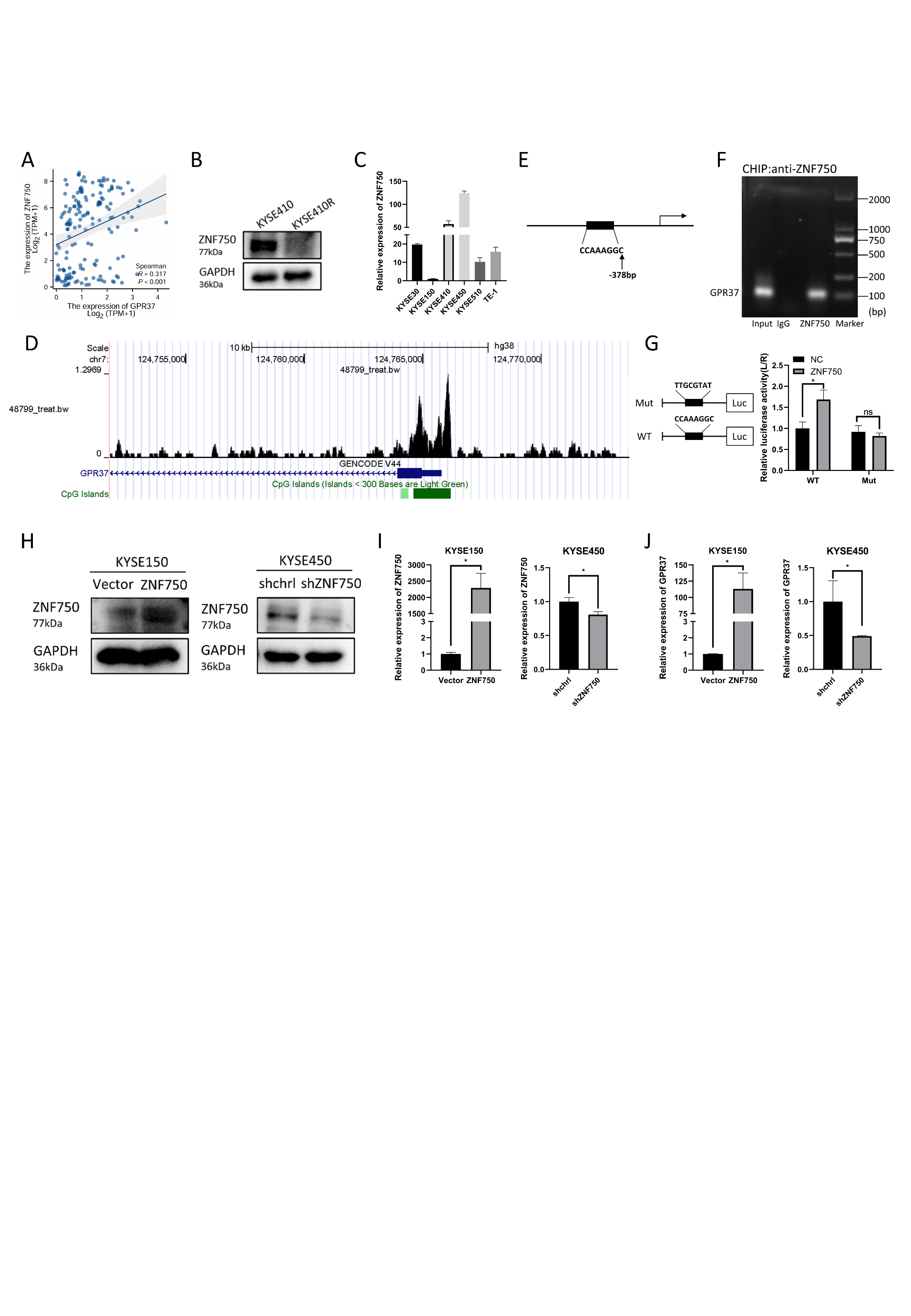


GPR37:


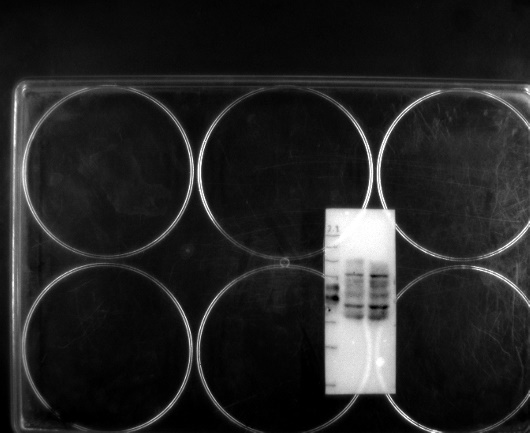




| lane | 1 | 2 | 3 |
| --- | --- | --- | --- |
| sample | marker | KYSE150  Vector | KYSE150  ZNF750 |

GAPDH:


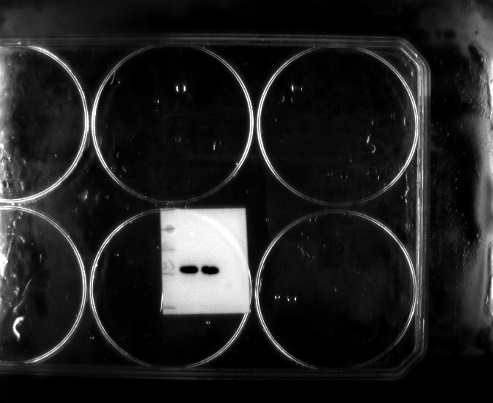




| lane | 1 | 2 | 3 |
| --- | --- | --- | --- |
| sample | marker | KYSE150  Vector | KYSE150  ZNF750 |

GPR37:


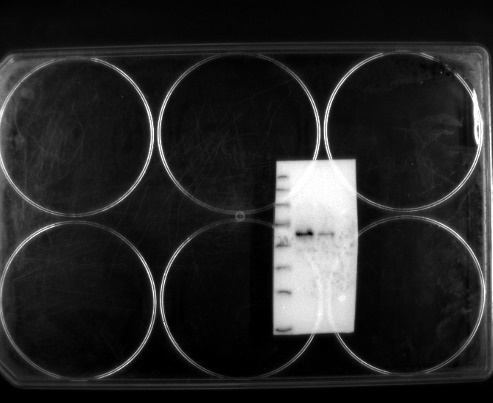




| lane | 1 | 2 | 3 |
| --- | --- | --- | --- |
| sample | marker | KYSE450  shchrl | KYSE450  ShZNF750 |

GAPDH:


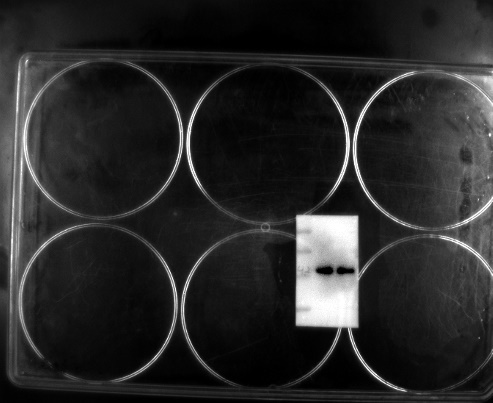




| lane | 1 | 2 | 3 |
| --- | --- | --- | --- |
| sample | marker | KYSE450  shchrl | KYSE450  ShZNF750 |

Corresponds to Fig 3.K in the article:


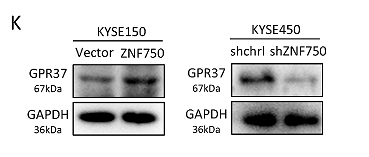


GPR37:


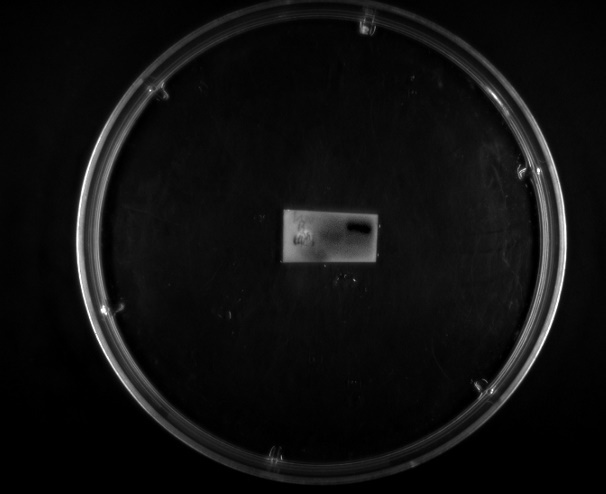




| lane | 1 | 2 | 3 |
| --- | --- | --- | --- |
| sample | marker | KYSE150 Vector | KYSE150GPR37 |

GAPDH:


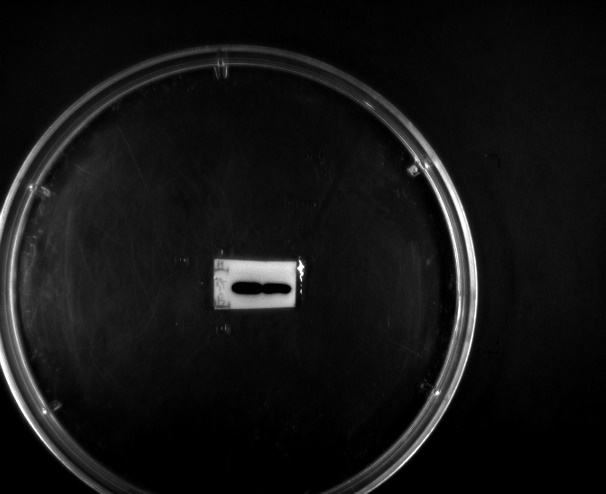




| lane | 1 | 2 | 3 |
| --- | --- | --- | --- |
| sample | marker | KYSE150 Vector | KYSE150GPR37 |

GPR37:


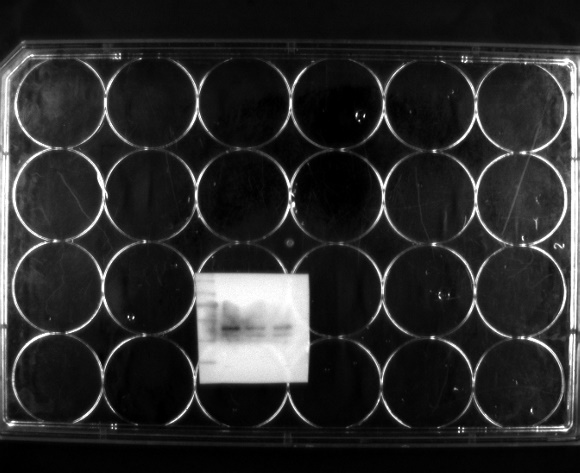




| lane | 1 | 2 | 3 | 4 |
| --- | --- | --- | --- | --- |
| sample | marker | KYSE450shchrl | KYSE450 shGPR37#1 | KYSE450 shGPR37#2 |

GAPDH:


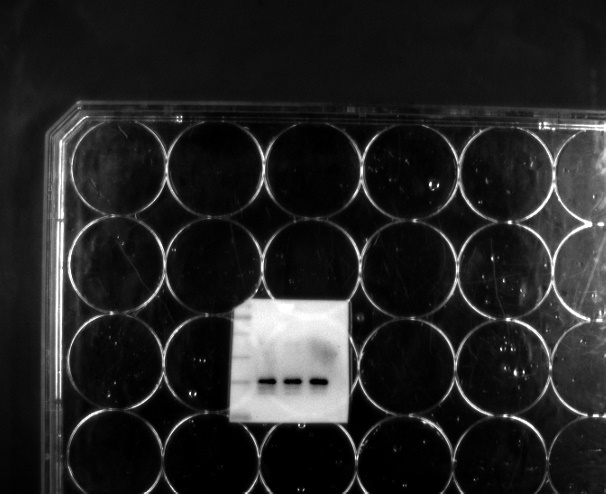




| lane | 1 | 2 | 3 | 4 |
| --- | --- | --- | --- | --- |
| sample | marker | KYSE450 | KYSE450shchrl | KYSE450 shZNF750 |

Corresponds to Fig 4.A in the article:


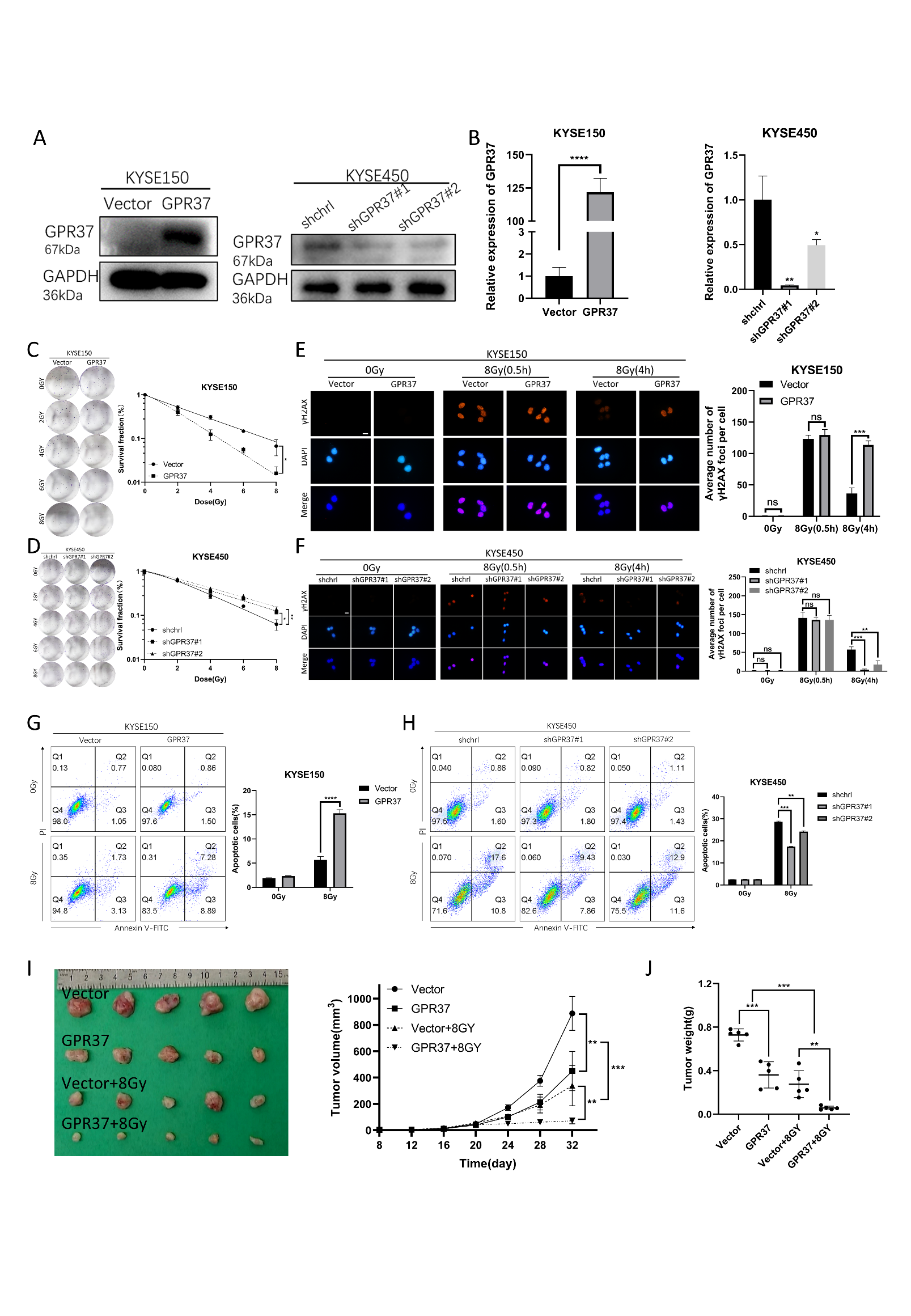


mTOR:


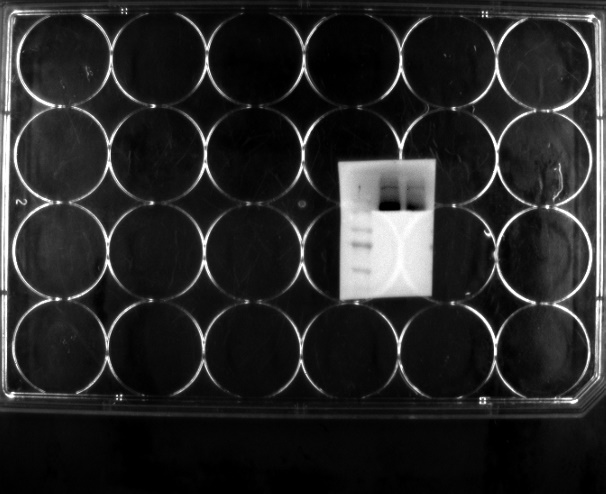




| lane | 1 | 2 | 3 |
| --- | --- | --- | --- |
| sample | marker | KYSE150Vector | KYSE150 GPR37 |

P-AKT:







| lane | 1 | 2 | 3 |
| --- | --- | --- | --- |
| sample | KYSE150 GPR37 | KYSE150Vector | marker |

AKT:


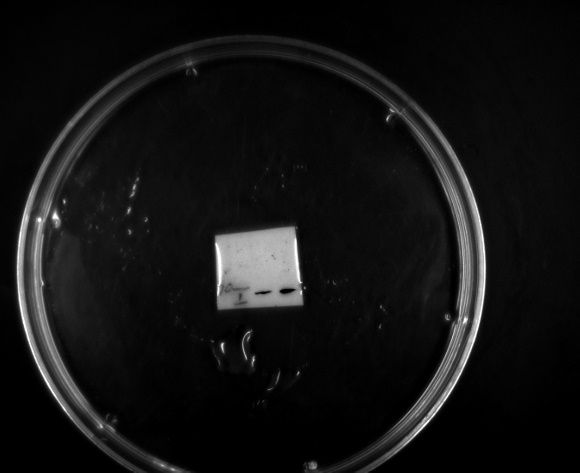




| lane | 1 | 2 | 3 |
| --- | --- | --- | --- |
| sample | marker | KYSE150Vector | KYSE150 GPR37 |

RAD51:







| lane | 1 | 2 | 3 | 4 | 5 | 6 |
| --- | --- | --- | --- | --- | --- | --- |
| sample | KYSE510 GPR37 | KYSE510Vector | marker | KYSE150 GPR37 | KYSE150Vector | marker |

Bcl-2:


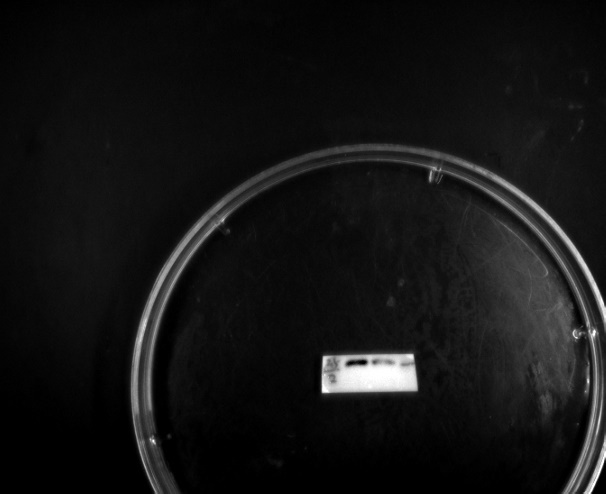




| lane | 1 | 2 | 3 | 4 |
| --- | --- | --- | --- | --- |
| sample | marker | KYSE150Vector | KYSE150 GPR37 | marker |

GAPDH:


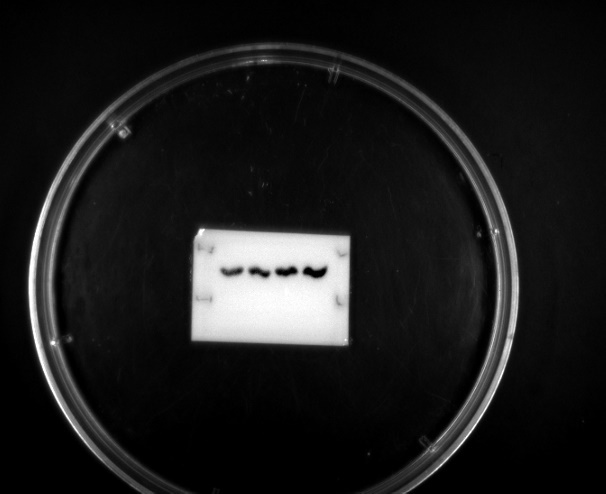




| lane | 1 | 2 | 3 | 4 | 5 | 6 |
| --- | --- | --- | --- | --- | --- | --- |
| sample | marker | KYSE510 GPR37 | KYSE510Vector | KYSE150 GPR37 | KYSE150Vector | marker |

Corresponds to Fig 6.B in the article:


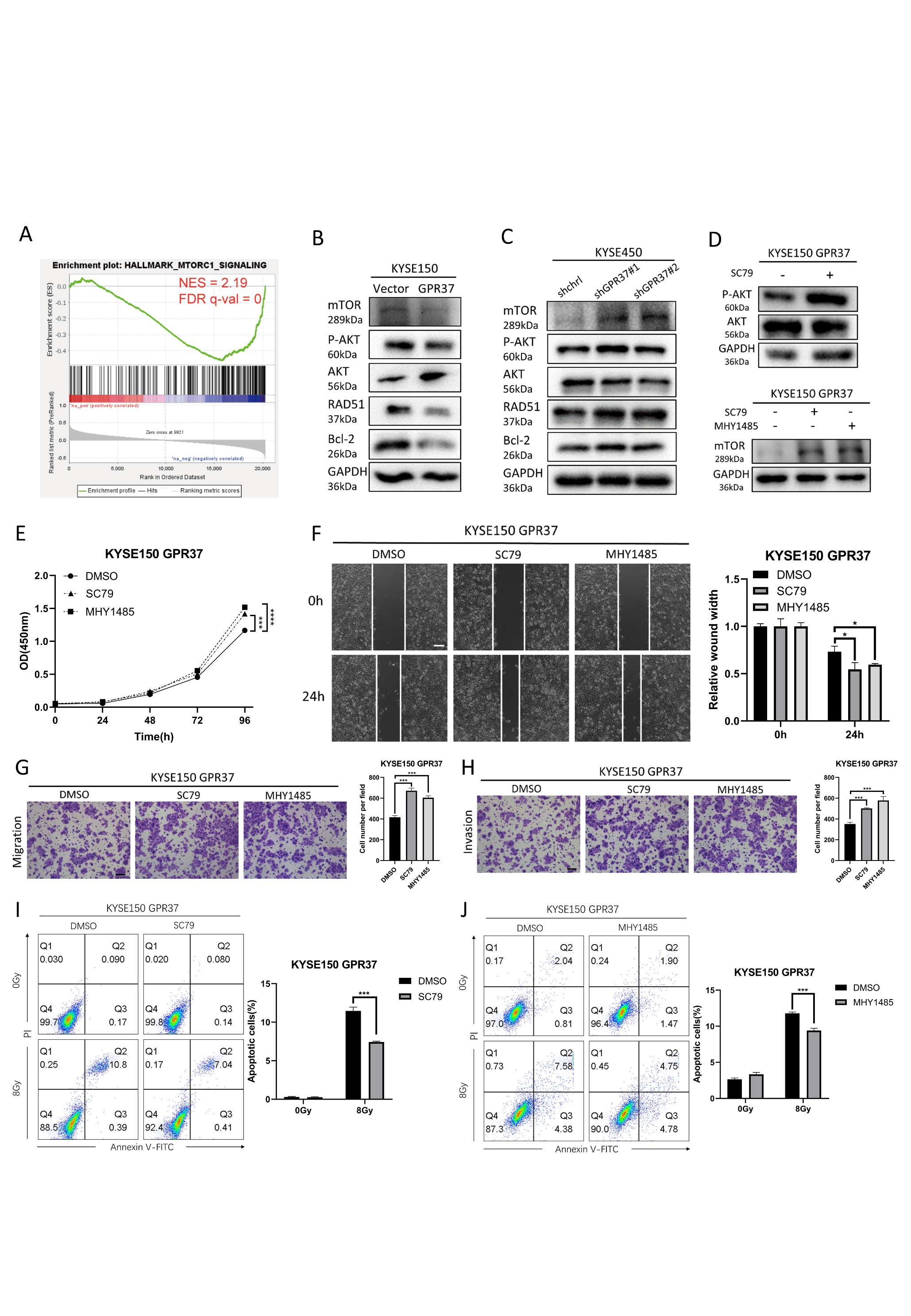


mTOR:


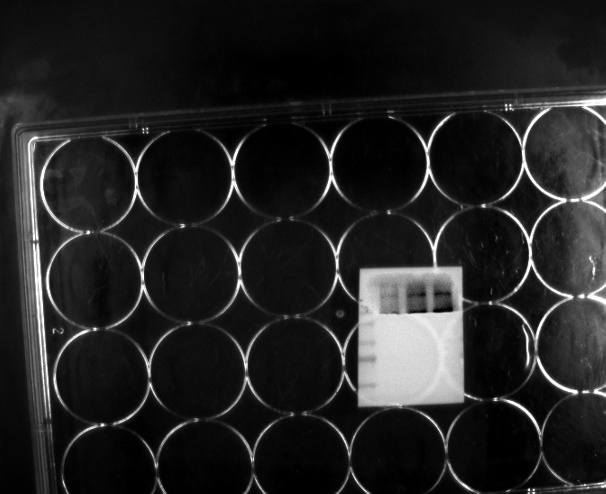




| lane | 1 | 2 | 3 | 4 |
| --- | --- | --- | --- | --- |
| sample | marker | KYSE450shchrl | KYSE450 shGPR37#1 | KYSE450 shGPR37#2 |

P-AKT:


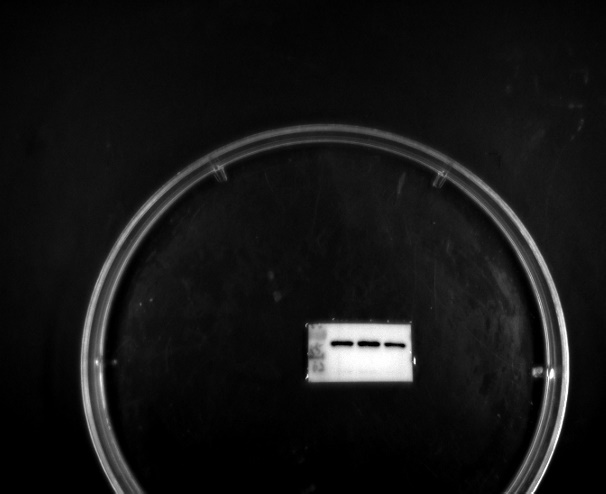




| lane | 1 | 2 | 3 | 4 |
| --- | --- | --- | --- | --- |
| sample | KYSE450 shGPR37#2 | KYSE450 shGPR37#1 | KYSE450 shchrl | marker |

AKT:


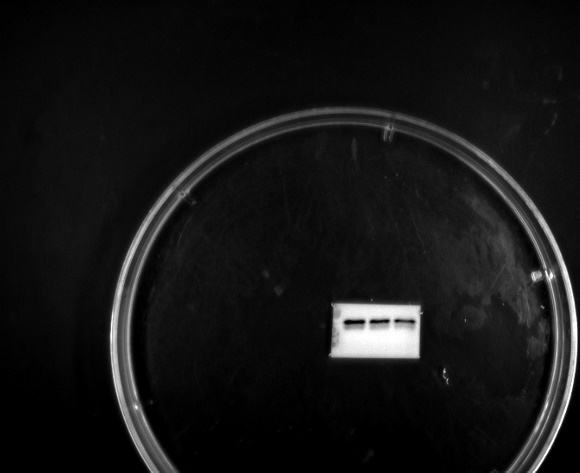




| lane | 1 | 2 | 3 | 4 |
| --- | --- | --- | --- | --- |
| sample | marker | KYSE450shchrl | KYSE450 shGPR37#1 | KYSE450 shGPR37#2 |

RAD51:







| lane | 1 | 2 | 3 | 4 |
| --- | --- | --- | --- | --- |
| sample | marker | KYSE450shchrl | KYSE450 shGPR37#1 | KYSE450 shGPR37#2 |

Bcl-2:


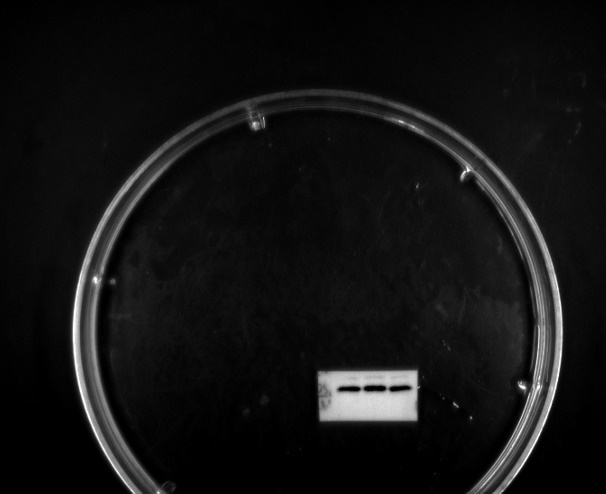




| lane | 1 | 2 | 3 | 4 |
| --- | --- | --- | --- | --- |
| sample | marker | KYSE450shchrl | KYSE450 shGPR37#1 | KYSE450 shGPR37#2 |

GAPDH:


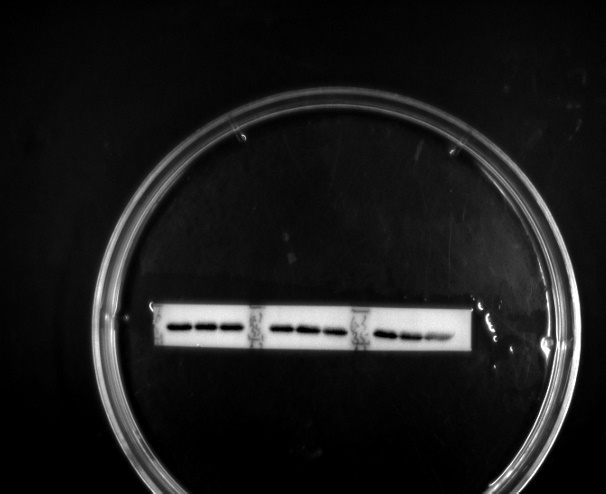




| lane | 1 | 2 | 3 | 4 | 5-12 |
| --- | --- | --- | --- | --- | --- |
| sample | marker | KYSE450shchrl | KYSE450 shGPR37#1 | KYSE450 shGPR37#2 | others |

Corresponds to Fig 6.C in the article:


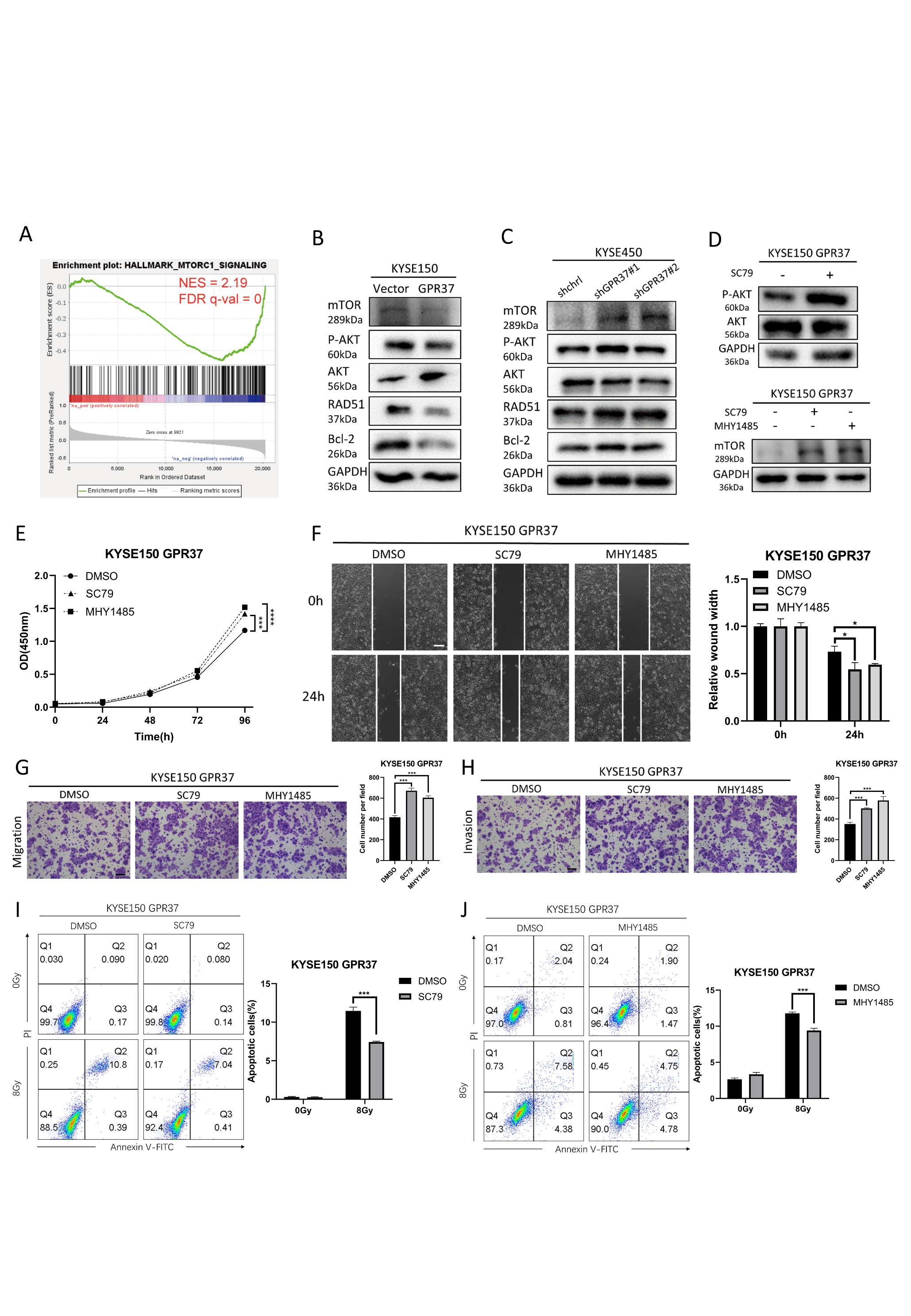


P-AKT:


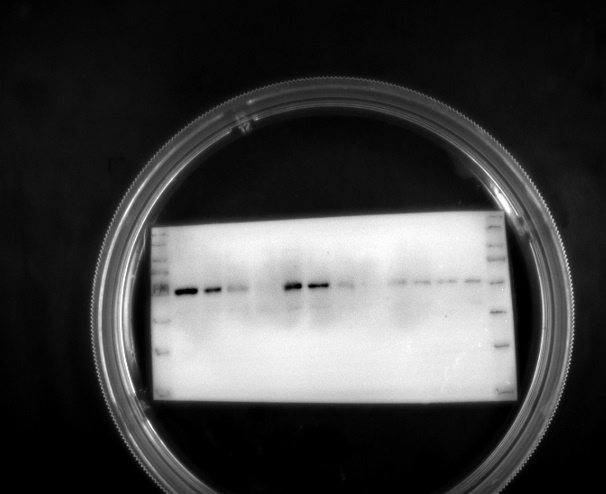




| lane | 1 | 2 | 3 | 4-13 | 14 |
| --- | --- | --- | --- | --- | --- |
| sample | marker | KYSE150GPR37+SC79 | KYSE150 GPR37+DMSO | others | marker |

AKT:


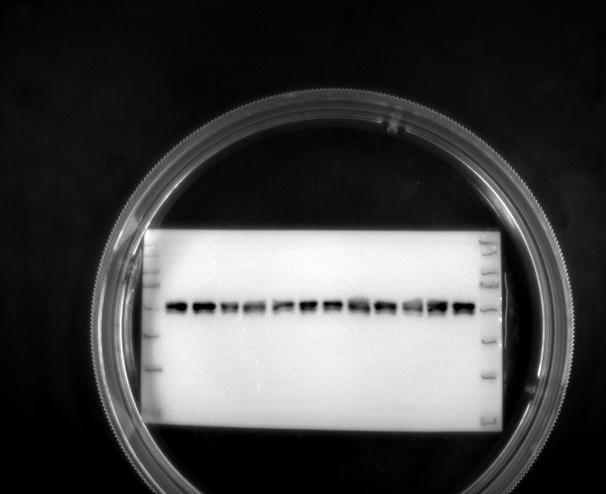




| lane | 1 | 2 | 3 | 4-13 | 14 |
| --- | --- | --- | --- | --- | --- |
| sample | marker | KYSE150GPR37+SC79 | KYSE150 GPR37+DMSO | others | marker |

GAPDH:


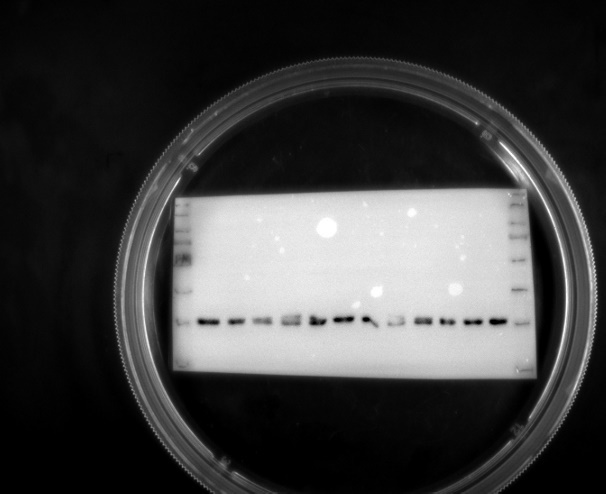




| lane | 1 | 2 | 3 | 4-13 | 14 |
| --- | --- | --- | --- | --- | --- |
| sample | marker | KYSE150GPR37+SC79 | KYSE150 GPR37+DMSO | others | marker |

mTOR:







| lane | 1 | 2 | 3 | 4 | 5 |
| --- | --- | --- | --- | --- | --- |
| sample | marker | KYSE150 GPR37+DMSO | KYSE150 GPR37+SC79 | KYSE150 GPR37+MHY1485 | KYSE150 |

GAPDH:


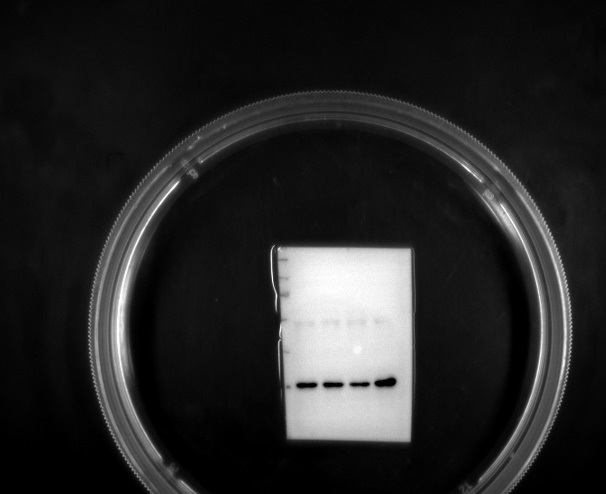




| lane | 1 | 2 | 3 | 4 | 5 |
| --- | --- | --- | --- | --- | --- |
| sample | marker | KYSE150 GPR37+DMSO | KYSE150 GPR37+SC79 | KYSE150 GPR37+MHY1485 | KYSE150 |

Corresponds to Fig 6.D in the article:


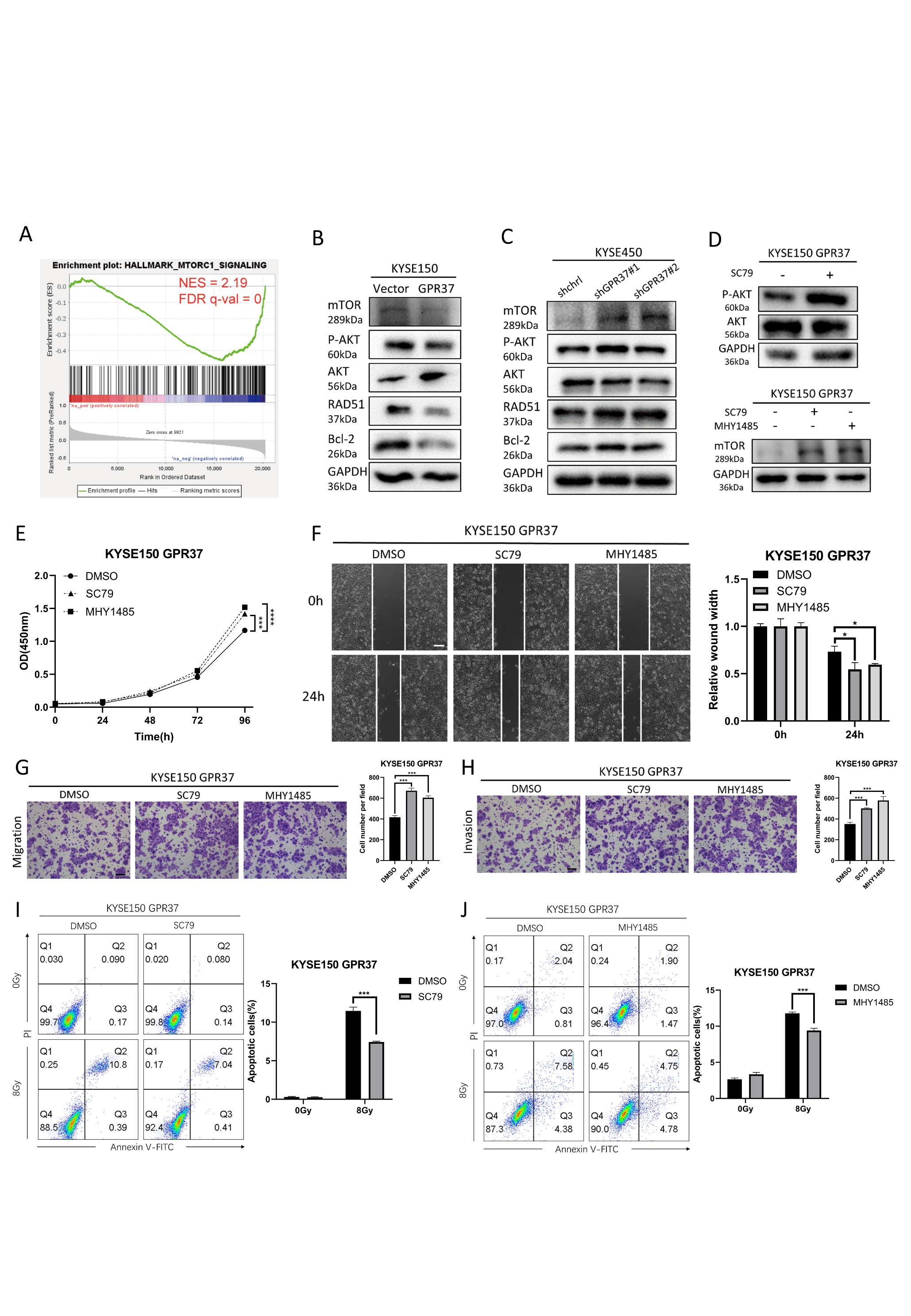


ATP1A1:


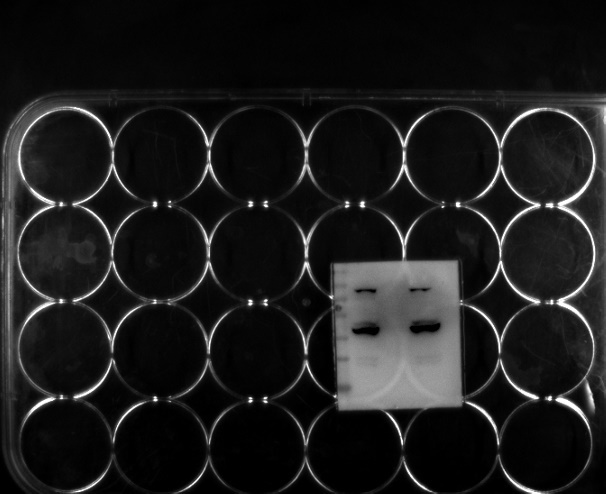




| lane | 1 | 2 | 3 | 4 |
| --- | --- | --- | --- | --- |
| sample | marker | input | Co-ip-IgG | Co-ip-GPR37 |

GPR37:


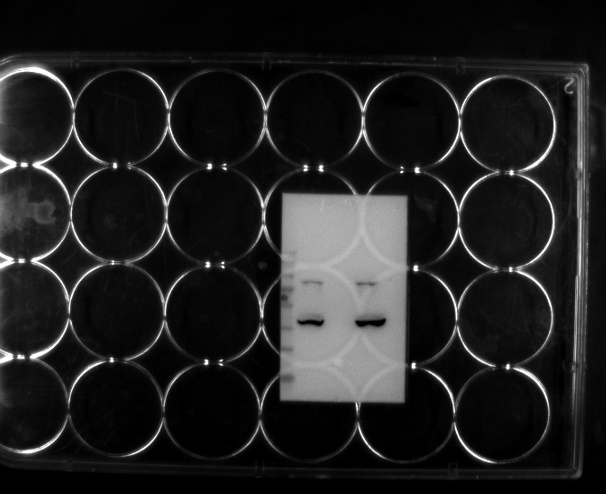




| lane | 1 | 2 | 3 | 4 |
| --- | --- | --- | --- | --- |
| sample | marker | input | Co-ip-IgG | Co-ip-GPR37 |

Corresponds to Fig 7.B in the article:


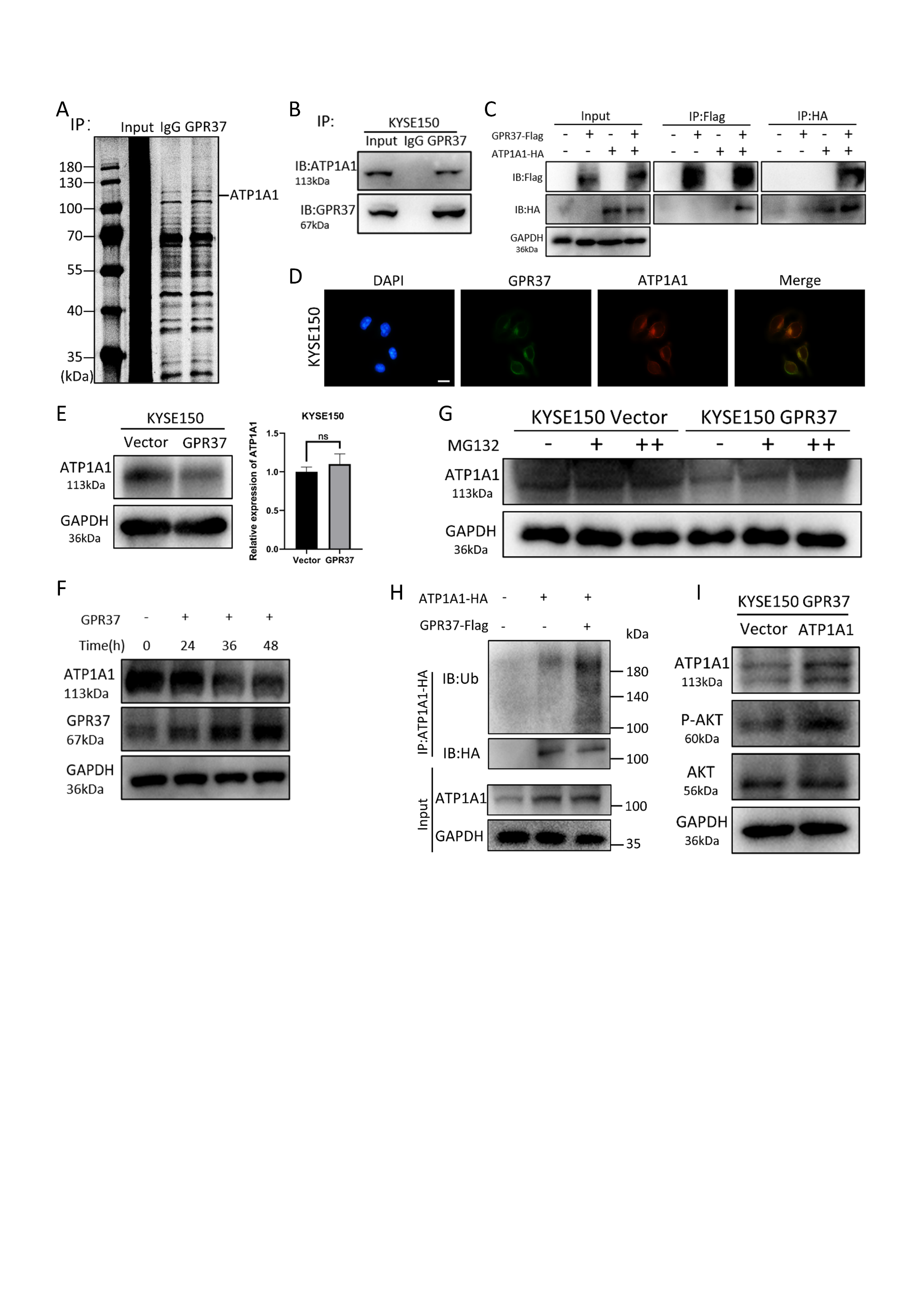


Flag:


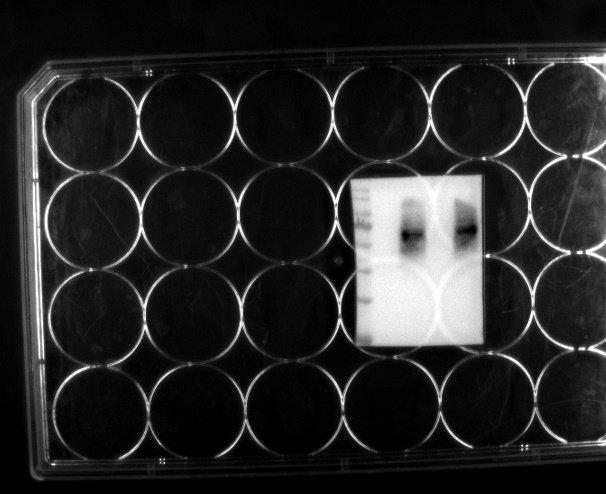




| lane | 1 | 2 | 3 | 4 | 5 |
| --- | --- | --- | --- | --- | --- |
| sample | marker | input | GPR37-Flag input | ATP1A1-HA input | GPR37-Flag+ATP1A1-HA input |

HA:


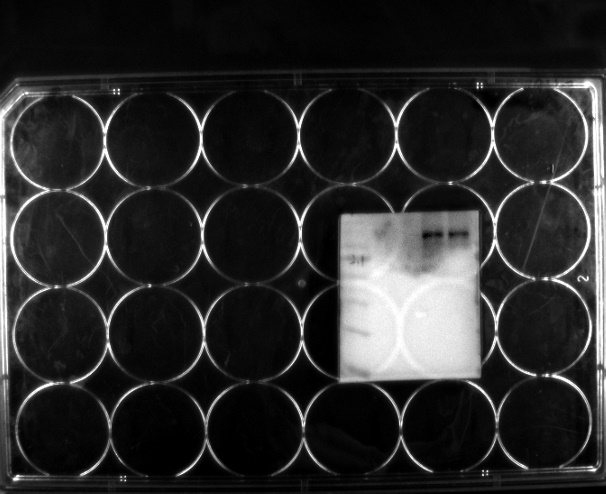




| lane | 1 | 2 | 3 | 4 | 5 |
| --- | --- | --- | --- | --- | --- |
| sample | marker | input | GPR37-Flag input | ATP1A1-HA input | GPR37-Flag+ATP1A1-HA input |

GAPDH:


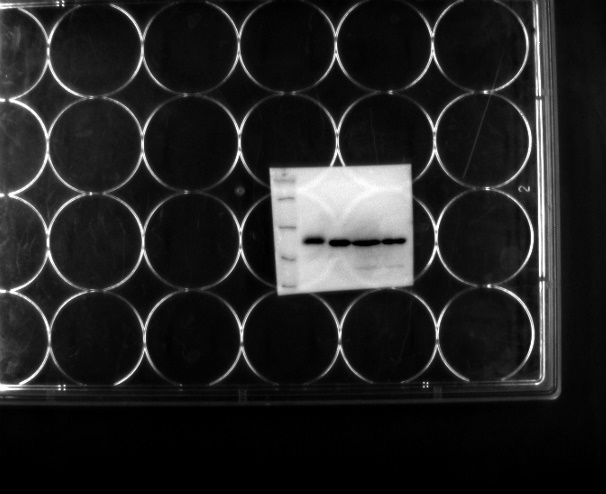




| lane | 1 | 2 | 3 | 4 | 5 |
| --- | --- | --- | --- | --- | --- |
| sample | marker | input | GPR37-Flag input | ATP1A1-HA input | GPR37-Flag+ATP1A1-HA input |

Flag:


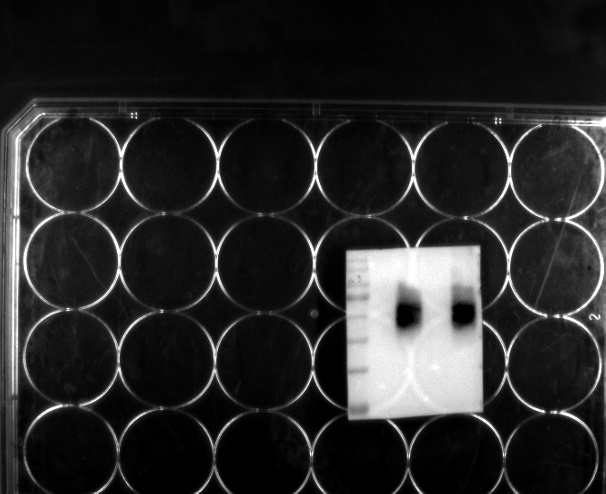


| lane | 1 | 2 | 3 | 4 | 5 |
| --- | --- | --- | --- | --- | --- |
| sample | marker | 293T IP-Flag | GPR37-Flag IP-Flag | ATP1A1-HA IP-Flag | GPR37-Flag+ATP1A1-HA IP-Flag |

HA:

| lane | 1 | 2 | 3 | 4 | 5 |
| --- | --- | --- | --- | --- | --- |
| sample | marker | 293T IP-Flag | GPR37-Flag IP-Flag | ATP1A1-HA IP-Flag | GPR37-Flag+ATP1A1-HA IP-Flag |

Flag:

| lane | 1 | 2 | 3 | 4 | 5 |
| --- | --- | --- | --- | --- | --- |
| sample | marker | 293T IP-HA | GPR37-Flag IP-HA | ATP1A1-HA IP-HA | GPR37-Flag+ATP1A1-HA IP-HA |

HA:

| lane | 1 | 2 | 3 | 4 | 5 |
| --- | --- | --- | --- | --- | --- |
| sample | marker | 293T IP-HA | GPR37-Flag IP-HA | ATP1A1-HA IP-HA | GPR37-Flag+ATP1A1-HA IP-HA |

Corresponds to Fig 7.C in the article:

ATP1A1:

| lane | 1 | 2 | 3 |
| --- | --- | --- | --- |
| sample | marker | KYSE150 Vector | KYSE150 GPR37 |

GAPDH:

| lane | 1 | 2 | 3 |
| --- | --- | --- | --- |
| sample | marker | KYSE150 Vector | KYSE150 GPR37 |

Corresponds to Fig 7.E in the article:

ATP1A1:

| lane | 1 | 2 | 3 | 4 | 5 | 6 | 7 |
| --- | --- | --- | --- | --- | --- | --- | --- |
| sample | marker | KYSE150 GPR37 0h | KYSE150 GPR37 24h | KYSE150 GPR37 36h | KYSE150 GPR37 48h | KYSE150 GPR37 60h | KYSE150 GPR37 72h |

GPR37:

| lane | 1 | 2 | 3 | 4 | 5 | 6 | 7 |
| --- | --- | --- | --- | --- | --- | --- | --- |
| sample | marker | KYSE150 GPR37 0h | KYSE150 GPR37 24h | KYSE150 GPR37 36h | KYSE150 GPR37 48h | KYSE150 GPR37 60h | KYSE150 GPR37 72h |

GAPDH:

| lane | 1 | 2 | 3 | 4 | 5 | 6 | 7 |
| --- | --- | --- | --- | --- | --- | --- | --- |
| sample | marker | KYSE150 GPR37 0h | KYSE150 GPR37 24h | KYSE150 GPR37 36h | KYSE150 GPR37 48h | KYSE150 GPR37 60h | KYSE150 GPR37 72h |

Corresponds to Fig 7.F in the article:

ATP1A1:

| lane | 1 | 2 | 3 | 4 | 5 | 6 | 7 |
| --- | --- | --- | --- | --- | --- | --- | --- |
| sample | marker | KYSE150 Vector+PBS | KYSE150 Vector+MG132 10 μM | KYSE150 Vector+MG132 20 μM | KYSE150 GPR37+PBS | KYSE150 GPR37+MG132 10 μM | KYSE150 GPR37+MG132 20 μM |

GAPDH:

| lane | 1 | 2 | 3 | 4 | 5 | 6 | 7 |
| --- | --- | --- | --- | --- | --- | --- | --- |
| sample | marker | KYSE150 Vector+PBS | KYSE150 Vector+MG132 10 μM | KYSE150 Vector+MG132 20 μM | KYSE150 GPR37+PBS | KYSE150 GPR37+MG132 10 μM | KYSE150 GPR37+MG132 20 μM |

Corresponds to Fig 7.G in the article:

UB:

| lane | 1 | 2 | 3 | 4 | 5 |
| --- | --- | --- | --- | --- | --- |
| sample | marker | 293T IP-HA | 293T+ATP1A1-HA IP-HA | 293T+ATP1A1-HA+GPR37-Flag IP-HA | marker |

HA:

| lane | 1 | 2 | 3 | 4 | 5 |
| --- | --- | --- | --- | --- | --- |
| sample | marker | 293T IP-HA | 293T+ATP1A1-HA IP-HA | 293T+ATP1A1-HA+GPR37-Flag IP-HA | marker |

ATP1A1:

| lane | 1 | 2 | 3 | 4 |
| --- | --- | --- | --- | --- |
| sample | marker | 293T input | 293T+ATP1A1-HA input | 293T+ATP1A1-HA+GPR37-Flag input |

GAPDH:

| lane | 1 | 2 | 3 | 4 |
| --- | --- | --- | --- | --- |
| sample | marker | 293T input | 293T+ATP1A1-HA input | 293T+ATP1A1-HA+GPR37-Flag input |

Corresponds to Fig 7.H in the article:

UB:

| lane | 1 | 2 | 3 | 4 | 5 |
| --- | --- | --- | --- | --- | --- |
| sample | marker | 293T IP-HA | 293T+ATP1A1-HA IP-HA | 293T+ATP1A1-HA+GPR37-Flag IP-HA | 293T+ATP1A1-HA+GPR37-Flag +Parkin-SI IP-HA |

HA:

| lane | 1 | 2 | 3 | 4 | 5 |
| --- | --- | --- | --- | --- | --- |
| sample | marker | 293T IP-HA | 293T+ATP1A1-HA IP-HA | 293T+ATP1A1-HA+GPR37-Flag IP-HA | 293T+ATP1A1-HA+GPR37-Flag +Parkin-SI IP-HA |

ATP1A1:

| lane | 1 | 2 | 3 | 4 | 5 |
| --- | --- | --- | --- | --- | --- |
| sample | marker | 293T Input | 293T+ATP1A1-HA Input | 293T+ATP1A1-HA+GPR37-Flag Input | 293T+ATP1A1-HA+GPR37-Flag +Parkin-SI Input |

GAPDH:

| lane | 1 | 2 | 3 | 4 | 5 |
| --- | --- | --- | --- | --- | --- |
| sample | marker | 293T Input | 293T+ATP1A1-HA Input | 293T+ATP1A1-HA+GPR37-Flag Input | 293T+ATP1A1-HA+GPR37-Flag +Parkin-SI Input |

Corresponds to Fig 7.J in the article:

ATP1A1:

| lane | 1 | 2 | 3 |
| --- | --- | --- | --- |
| sample | marker | KYSE150 GPR37+Vector | KYSE150 GPR37+ATP1A1 |

P-AKT:

| lane | 1 | 2 | 3 |
| --- | --- | --- | --- |
| sample | marker | KYSE150 GPR37+Vector | KYSE150 GPR37+ATP1A1 |

AKT:

| lane | 1 | 2 | 3 |
| --- | --- | --- | --- |
| sample | marker | KYSE150 GPR37+Vector | KYSE150 GPR37+ATP1A1 |

GAPDH:

| lane | 1 | 2 | 3 |
| --- | --- | --- | --- |
| sample | marker | KYSE150 GPR37+Vector | KYSE150 GPR37+ATP1A1 |

Corresponds to Fig 7.K in the article:

GPR37:

| lane | 1 | 2 | 3 | 4 |
| --- | --- | --- | --- | --- |
| sample | marker | WCL | Exo-vector | Exo-gpr37 |

TSG101:

| lane | 1 | 2 | 3 | 4 |
| --- | --- | --- | --- | --- |
| sample | marker | WCL | Exo-vector | Exo-gpr37 |

CD63:

| lane | 1 | 2 | 3 | 4 |
| --- | --- | --- | --- | --- |
| sample | WCL | Exo-vector | Exo-gpr37 | marker |

GAPDH:

| lane | 1 | 2 | 3 | 4 |
| --- | --- | --- | --- | --- |
| sample | marker | WCL | Exo-vector | Exo-gpr37 |

Corresponds to Fig 8.C in the article:

GPR37:

| lane | 1 | 2 | 3 | 4 |
| --- | --- | --- | --- | --- |
| sample | marker | KYSE150+PBS | KYSE150+Exo-vector | KYSE150+Exo-gpr37 |

GAPDH:

| lane | 1 | 2 | 3 | 4 | 5 |
| --- | --- | --- | --- | --- | --- |
| sample | marker | KYSE150+PBS | KYSE150+Exo-vector | KYSE150+Exo-gpr37 | marker |

Corresponds to Fig 8.F in the article:

mTOR:

| lane | 1 | 2 | 3 | 4 |
| --- | --- | --- | --- | --- |
| sample | marker | KYSE150+PBS | KYSE150+Exo-vector | KYSE150+Exo-gpr37 |

P-AKT:

| lane | 1 | 2 | 3 | 4 | 5 |
| --- | --- | --- | --- | --- | --- |
| sample | marker | KYSE150+PBS | KYSE150+Exo-vector | KYSE150+Exo-gpr37 | marker |

AKT:

| lane | 1 | 2 | 3 | 4 | 5 |
| --- | --- | --- | --- | --- | --- |
| sample | marker | KYSE150+PBS | KYSE150+Exo-vector | KYSE150+Exo-gpr37 | marker |

GAPDH:

| lane | 1 | 2 | 3 | 4 | 5 |
| --- | --- | --- | --- | --- | --- |
| sample | marker | KYSE150+PBS | KYSE150+Exo-vector | KYSE150+Exo-gpr37 | marker |

Corresponds to Fig 8.L in the article:

ZNF750:

| lane | 1 | 2 | 3 |
| --- | --- | --- | --- |
| sample | marker | KYSE510  Vector | KYSE510  ZNF750 |

GAPDH:

| lane | 1 | 2 | 3 |
| --- | --- | --- | --- |
| sample | marker | KYSE510  Vector | KYSE510  ZNF750 |

Corresponds to Supplemental Fig 3.A in the article:

GPR37:

| lane | 1 | 2 | 3 |
| --- | --- | --- | --- |
| sample | marker | KYSE510  Vector | KYSE510  ZNF750 |

GAPDH:

| lane | 1 | 2 | 3 |
| --- | --- | --- | --- |
| sample | marker | KYSE510  Vector | KYSE510  ZNF750 |

Corresponds to Supplemental Fig 3.D in the article:

GPR37:

| lane | 1 | 2 | 3 |
| --- | --- | --- | --- |
| sample | KYSE510Vector | KYSE510GPR37 | marker |

GAPDH:

| lane | 1 | 2 | 3 | 4 |
| --- | --- | --- | --- | --- |
| sample | marker | KYSE510Vector | KYSE510GPR37 | marker |

Corresponds to Supplemental Fig 4.D in the article:

N-cad:

| lane | 1 | 2 | 3 |
| --- | --- | --- | --- |
| sample | marker | KYSE150Vector | KYSE150 GPR37 |

E-cad:

| lane | 1 | 2 | 3 |
| --- | --- | --- | --- |
| sample | KYSE150 GPR37 | KYSE150Vector | marker |

Slug:

| lane | 1 | 2 | 3 |
| --- | --- | --- | --- |
| sample | marker | KYSE150Vector | KYSE150 GPR37 |

GAPDH:

| lane | 1 | 2 | 3 | 4 | 5 | 6 |
| --- | --- | --- | --- | --- | --- | --- |
| sample | marker | KYSE510 GPR37 | KYSE510Vector | KYSE150 GPR37 | KYSE150Vector | marker |

N-cad:

| lane | 1 | 2 | 3 |
| --- | --- | --- | --- |
| sample | marker | KYSE510Vector | KYSE510 GPR37 |

E-cad:

| lane | 1 | 2 | 3 |
| --- | --- | --- | --- |
| sample | marker | KYSE510Vector | KYSE510 GPR37 |

Slug:

| lane | 1 | 2 | 3 |
| --- | --- | --- | --- |
| sample | marker | KYSE510Vector | KYSE510 GPR37 |

GAPDH:

| lane | 1 | 2 | 3 |
| --- | --- | --- | --- |
| sample | marker | KYSE510Vector | KYSE510 GPR37 |

N-cad:

| lane | 1 | 2 | 3 | 4 |
| --- | --- | --- | --- | --- |
| sample | marker | KYSE450shchrl | KYSE450 shGPR37#1 | KYSE450 shGPR37#2 |

E-cad:

| lane | 1 | 2 | 3 | 4 |
| --- | --- | --- | --- | --- |
| sample | marker | KYSE450shchrl | KYSE450 shGPR37#1 | KYSE450 shGPR37#2 |

Slug:

| lane | 1 | 2 | 3 | 4 |
| --- | --- | --- | --- | --- |
| sample | marker | KYSE450shchrl | KYSE450 shGPR37#1 | KYSE450 shGPR37#2 |

GAPDH:

| lane | 1 | 2 | 3 | 4 | 5-12 |
| --- | --- | --- | --- | --- | --- |
| sample | marker | KYSE450shchrl | KYSE450 shGPR37#1 | KYSE450 shGPR37#2 | others |

Corresponds to Supplemental Fig 5.I in the article:

mTOR:

| lane | 1 | 2 | 3 |
| --- | --- | --- | --- |
| sample | marker | KYSE510Vector | KYSE510 GPR37 |

P-AKT:

| lane | 1 | 2 | 3 |
| --- | --- | --- | --- |
| sample | marker | KYSE510Vector | KYSE510 GPR37 |

AKT:

| lane | 1 | 2 | 3 |
| --- | --- | --- | --- |
| sample | marker | KYSE510Vector | KYSE510 GPR37 |

RAD51:

| lane | 1 | 2 | 3 |
| --- | --- | --- | --- |
| sample | marker | KYSE510Vector | KYSE510 GPR37 |

Bcl-2:

| lane | 1 | 2 | 3 |
| --- | --- | --- | --- |
| sample | marker | KYSE510Vector | KYSE510 GPR37 |

GAPDH:

| lane | 1 | 2 | 3 |
| --- | --- | --- | --- |
| sample | marker | KYSE510Vector | KYSE510 GPR37 |

Corresponds to Supplemental Fig 6.A in the article:

P-AKT:

| lane | 1 | 2-4 | 5 | 6 | 7-13 | 14 |
| --- | --- | --- | --- | --- | --- | --- |
| sample | marker | others | KYSE510GPR37+SC79 | KYSE510 GPR37+DMSO | others | marker |

AKT:

| lane | 1 | 2-4 | 5 | 6 | 7-13 | 14 |
| --- | --- | --- | --- | --- | --- | --- |
| sample | marker | others | KYSE510GPR37+SC79 | KYSE510 GPR37+DMSO | others | marker |

GAPDH:

| lane | 1 | 2-4 | 5 | 6 | 7-13 | 14 |
| --- | --- | --- | --- | --- | --- | --- |
| sample | marker | others | KYSE510GPR37+SC79 | KYSE510 GPR37+DMSO | others | marker |

mTOR:

| lane | 1 | 2 | 3 | 4 | 5 |
| --- | --- | --- | --- | --- | --- |
| sample | marker | KYSE510 | KYSE510 GPR37+DMSO | KYSE510 GPR37+SC79 | KYSE510 GPR37+MHY1485 |

GAPDH:

| lane | 1 | 2 | 3 | 4 | 5 | 6 |
| --- | --- | --- | --- | --- | --- | --- |
| sample | marker | KYSE510 | KYSE510 GPR37+DMSO | KYSE510 GPR37+SC79 | KYSE510 GPR37+MHY1485 | marker |

Corresponds to Supplemental Fig 6.B in the article:

P-AKT:

| lane | 1 | 2 | 3 |
| --- | --- | --- | --- |
| sample | marker | KYSE450shGPR37#1+DMSO | KYSE450shGPR37#1+MK2206 |

AKT:

| lane | 1 | 2 | 3 | 4 |
| --- | --- | --- | --- | --- |
| sample | marker | KYSE450shGPR37#1+DMSO | KYSE450shGPR37#1+MK2206 | marker |

GAPDH:

| lane | 1 | 2 | 3 | 4 |
| --- | --- | --- | --- | --- |
| sample | marker | KYSE450shGPR37#1+DMSO | KYSE450shGPR37#1+MK2206 | marker |

mTOR:

| lane | 1 | 2 | 3 | 4 |
| --- | --- | --- | --- | --- |
| sample | marker | KYSE450shGPR37#1+DMSO | KYSE450shGPR37#1+MK2206 | KYSE450shGPR37#1+TORIN1 |

GAPDH:

| lane | 1 | 2 | 3 | 4 |
| --- | --- | --- | --- | --- |
| sample | marker | KYSE450shGPR37#1+DMSO | KYSE450shGPR37#1+MK2206 | KYSE450shGPR37#1+TORIN1 |

P-AKT:

| lane | 1 | 2 | 3 | 4 |
| --- | --- | --- | --- | --- |
| sample | marker | KYSE450shGPR37#2+DMSO | KYSE450shGPR37#2+MK2206 | marker |

AKT:

| lane | 1 | 2 | 3 | 4 |
| --- | --- | --- | --- | --- |
| sample | marker | KYSE450shGPR37#2+DMSO | KYSE450shGPR37#2+MK2206 | marker |

GAPDH:

| lane | 1 | 2 | 3 | 4 |
| --- | --- | --- | --- | --- |
| sample | marker | KYSE450shGPR37#2+DMSO | KYSE450shGPR37#2+MK2206 | marker |

mTOR:

| lane | 1 | 2 | 3 | 4 |
| --- | --- | --- | --- | --- |
| sample | marker | KYSE450shGPR37#2+DMSO | KYSE450shGPR37#2+MK2206 | KYSE450shGPR37#2+TORIN1 |

GAPDH:

| lane | 1 | 2 | 3 | 4 |
| --- | --- | --- | --- | --- |
| sample | marker | KYSE450shGPR37#2+DMSO | KYSE450shGPR37#2+MK2206 | KYSE450shGPR37#2+TORIN1 |

Corresponds to Supplemental Fig 7.A in the article:

ATP1A1:

| lane | 1 | 2 | 3 | 4 |
| --- | --- | --- | --- | --- |
| sample | marker | KYSE510 input | KYSE510 Co-ip-IgG | KYSE510 Co-ip-GPR37 |

GPR37:

| lane | 1 | 2 | 3 | 4 |
| --- | --- | --- | --- | --- |
| sample | marker | KYSE510 input | KYSE510 Co-ip-IgG | KYSE510 Co-ip-GPR37 |

Corresponds to Supplemental Fig 8.A in the article:

ATP1A1:

| lane | 1 | 2 | 3 |
| --- | --- | --- | --- |
| sample | KYSE510 GPR37 | KYSE510 Vector | marker |

GAPDH:

| lane | 1 | 2 | 3 |
| --- | --- | --- | --- |
| sample | KYSE510 GPR37 | KYSE510 Vector | marker |

Corresponds to Supplemental Fig 8.C in the article:

ATP1A1:

| lane | 1 | | 2 | 3 | 4 | 5 | 6 | 7 |
| --- | --- | --- | --- | --- | --- | --- | --- | --- |
| sample | marker | KYSE510 Vector+PBS | | KYSE510 Vector+MG132 10 μM | KYSE510 Vector+MG132 20 μM | KYSE510 GPR37+ PBS | KYSE510 GPR37+MG132 10 μM | KYSE510 GPR37+MG132 20 μM |

GAPDH:

| lane | 1 | | 2 | 3 | 4 | 5 | 6 | 7 |
| --- | --- | --- | --- | --- | --- | --- | --- | --- |
| sample | marker | KYSE510 Vector+PBS | | KYSE510 Vector+MG132 10 μM | KYSE510 Vector+MG132 20 μM | KYSE510 GPR37+ PBS | KYSE510 GPR37+MG132 10 μM | KYSE510 GPR37+MG132 20 μM |

Corresponds to Supplemental Fig 8.D in the article:

ATP1A1:

| lane | 1 | 2 | 3 |
| --- | --- | --- | --- |
| sample | marker | KYSE510 GPR37+Vector | KYSE510 GPR37+ATP1A1 |

P-AKT:

| lane | 1 | 2 | 3 |
| --- | --- | --- | --- |
| sample | marker | KYSE510 GPR37+Vector | KYSE510 GPR37+ATP1A1 |

AKT:

| lane | 1 | 2 | 3 |
| --- | --- | --- | --- |
| sample | marker | KYSE510 GPR37+Vector | KYSE510 GPR37+ATP1A1 |

GAPDH:

| lane | 1 | 2 | 3 |
| --- | --- | --- | --- |
| sample | marker | KYSE510 GPR37+Vector | KYSE510 GPR37+ATP1A1 |

Corresponds to Supplemental Fig 8.E in the article:

GPR37:

| lane | 1 | 2 | 3 | 4 |
| --- | --- | --- | --- | --- |
| sample | marker | WCL | Exo-Vector | Exo-GPR37 |

TSG101:

| lane | 1 | 2 | 3 | 4 |
| --- | --- | --- | --- | --- |
| sample | marker | WCL | Exo-Vector | Exo-GPR37 |

CD63:

| lane | 1 | 2 | 3 | 4 |
| --- | --- | --- | --- | --- |
| sample | marker | WCL | Exo-Vector | Exo-GPR37 |

GAPDH:

| lane | 1 | 2 | 3 | 4 |
| --- | --- | --- | --- | --- |
| sample | marker | WCL | Exo-Vector | Exo-GPR37 |

Corresponds to Supplemental Fig 9.C in the article:

GPR37:

| lane | 1 | 2 | 3 | 4 |
| --- | --- | --- | --- | --- |
| sample | marker | KYSE510+Exo-GPR37 | KYSE510+Exo-Vector | KYSE510+PBS |

GAPDH:

| lane | 1 | 2 | 3 | 4 |
| --- | --- | --- | --- | --- |
| sample | marker | KYSE510+Exo-GPR37 | KYSE510+Exo-Vector | KYSE510+PBS |

Corresponds to Supplemental Fig 9.E in the article:

mTOR:

| lane | 1 | 2 | 3 | 4 |
| --- | --- | --- | --- | --- |
| sample | marker | KYSE510+PBS | KYSE510+Exo-Vector | KYSE510+Exo-GPR37 |

P-AKT:

| lane | 1 | 2 | 3 | 4 |
| --- | --- | --- | --- | --- |
| sample | marker | KYSE510+PBS | KYSE510+Exo-Vector | KYSE510+Exo-GPR37 |

AKT:

| lane | 1 | 2 | 3 | 4 |
| --- | --- | --- | --- | --- |
| sample | marker | KYSE510+PBS | KYSE510+Exo-Vector | KYSE510+Exo-GPR37 |

GAPDH:

| lane | 1 | 2 | 3 | 4 |
| --- | --- | --- | --- | --- |
| sample | marker | KYSE510+PBS | KYSE510+Exo-Vector | KYSE510+Exo-GPR37 |

Corresponds to Supplemental Fig 9.J in the article:
